# Preventing Cognitive Frailty: What People Know and What They Are Willing to Change

**DOI:** 10.1002/brb3.71307

**Published:** 2026-03-23

**Authors:** Shaimaa Mohammed Elhag, Tasmin Alanna Rookes, Malwina Agnieszka Niechcial, Mohaddeseh Ziyachi, Ruoyu Wang, Millennium Soibifaa Iyobuchiebomie, Alan John Gow

**Affiliations:** ^1^ Department of Psychology, School of Social Sciences Heriot‐Watt University Edinburgh UK; ^2^ Research Department of Primary Care and Population Health University College London London UK; ^3^ Department of Sociology Durham University Durham UK; ^4^ Institute of Public Health and Wellbeing University of Essex Essex UK; ^5^ Centre For Applied Health and Social Care Research Sheffield Hallam University Sheffield UK

**Keywords:** behavior change, cognitive frailty, cross‐sectional survey, health promotion, prevention, underserved groups

## Abstract

**Purpose:**

Cognitive frailty (CF), defined as the co‐existence of physical frailty and cognitive impairment in the absence of dementia, may be preventable. Interventions could target modifiable risk factors that promote healthy aging. However, awareness of public knowledge of CF and willingness to engage in preventative behaviors, particularly across demographic groups, remains limited.

**Method:**

To assess awareness of CF, current health behaviors, and attitudes toward the preventing it, we conducted a cross‐sectional survey, collecting responses from people aged 21 and older and living in the United Kingdom. The survey assessed participants’ beliefs, knowledge, and behaviors related to CF. Levels of knowledge and behaviors were compared across demographic groups, including age, gender, ethnicity, deprivation, education, and health status. Logistic regression analyses were used to assess whether familiarity with CF and beliefs about its preventability were associated with perceptions of how important different behaviors are and current and future behavior engagement.

**Finding:**

A total of 4520 adults (aged 21–97 years, 70% male, 97% white, 60% having an undergraduate or postgraduate qualification) participated. Most respondents reported good knowledge of CF (80%) and believed CF could be prevented (91%). Participants engaging in behaviors to prevent CF and willing to engage in these behaviors in the future were more likely to be older (60–79 years old), male, white, and highly educated. Those from underserved groups were less likely to be engaging in health behaviors associated with lower risk of CF but reported willingness to engage in the future. Self‐rated health was the biggest barrier to willingness to engage in future behaviors.

**Conclusion:**

The findings suggest that while some groups are less engaged in healthy aging behaviors, their willingness to adopt preventative strategies highlights the potential of targeted interventions. Tailoring approaches to specific demographic groups might enhance engagement and support equitable promotion of healthy aging to prevent CF.

## Graphical abstract

Cognitive frailty (CF) combines physical frailty and cognitive impairment and may be preventable through healthy aging behaviors. We surveyed 4520 adults across the United Kingdom to assess their knowledge of CF, current lifestyle behaviors, and willingness to engage in prevention. Most participants reported good knowledge of CF and believed it could be prevented. Engagement in preventative behaviors was highest among older, white, male, and highly educated respondents. Although underserved groups were less likely to be engaging in these behaviors currently, they expressed strong willingness to adopt them in the future. Poor self‑rated health was the main barrier. These findings highlight the potential for targeted interventions to support the equitable promotion of healthy aging.

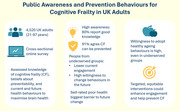

## 1 Introduction

The global demographic landscape is undergoing a significant transformation, with the number of people aged 60 years and older expected to double to 2.1 billion by 2050 (WHO 2024). The number of people aged 80 years or older is projected to triple, reaching 426 million during the same period (WHO 2024). These global patterns are visible at the national level, with the number of adults aged 85 years and older predicted to almost double from 1.7 million to 3.3 million between 2022 and 2047 in the United Kingdom (ONS 2025). Such demographic shifts contribute to a growing prevalence of frailty among older adults (Holland et al. 2024). Frailty is characterized by reduced physiological reserve and resilience to stressors, making older adults more vulnerable to adverse outcomes such as falls, disability, hospitalization, and mortality (Sternberg et al. 2011).

Research has identified a strong association between physical frailty and cognitive impairment (Halil et al. 2015), defined as difficulties with memory, thinking, problem‐solving, and language (Gauthier et al. 2006). When these co‐occur, there is an increased risk of disability and functional decline (Makizako et al. 2015; Rivan et al. 2021; Roppolo et al. 2017; Shimada et al. 2016). This co‐occurrence of physical frailty and cognitive impairment in the absence of dementia, was conceptualized as cognitive frailty (CF) (Ruan et al. 2015). CF is of particular concern as it increases the risk of neurodegenerative conditions such as dementia. Around 55 million people currently live with dementia globally, including around 1 million in the United Kingdom. CF also increases the likelihood of other adverse outcomes such as disability, hospital admissions, poor quality of life, and death (Sugimoto et al. 2022).

CF is multidimensional, encompassing physical impairments, cognitive decline, nutritional deficiencies, and socioeconomic disadvantages (Ruan et al. 2020). CF is potentially reversible, particularly when identified early. Research suggests that CF and dementia share similar life course risk factors, including low physical activity, poor nutrition, limited education, and metabolic disorders (Panza et al. 2018; Ruan et al. 2015). While attention has been given to physical frailty as a clinical condition, there is increasing interest in the possibility of mitigating CF through targeted interventions that enhance both physical and cognitive resilience (Clegg et al. 2013; Fowler Davis et al. 2024; Rockwood et al. 2005).

A key component of CF, however, is the sense of its reversibility. Preventative strategies such as regular physical activity, adequate protein and vitamin D intake, not smoking, and maintenance of a healthy body weight have been shown to reduce the risk or reverse early stages of CF (Zuliani et al. 2015). Psychological and social factors, such as social engagement and perceived quality of life, also play a significant role in the onset and progression of CF (Langlois et al. 2012). Evidence from a cross‐sectional study examining physical frailty in over 8,600 community‐dwelling adults demonstrated that physical frailty severity is associated with demographic and social differences, with frailer individuals more likely to be older, female, unmarried, and socially isolated (Op het Veld et al. 2015). However, similar data on CF is lacking in the literature.

Recent Delphi studies underscore the importance people with lived and professional experience place on physical and psychosocial components, such as social support, mental health, and community environment, in the early development of CF and pre‐frailty (Holland et al. 2024; Sezgin et al. 2022). This has prompted increased interest in designing interventions that address the multidimensional nature of CF. Preventative approaches targeting modifiable risk factors offer the potential to delay or reduce the onset of CF.

Despite the clinical significance and potential reversibility of CF, limited evidence exists on public awareness of CF. A recent systematic review highlighted disparities in knowledge of brain health and beliefs about the preventability of cognitive decline, with older adults, men, and individuals with lower education having less knowledge (Niechcial et al. 2025). However, most studies included in the review focused on middle‐aged and older adults, with information lacking for younger adults and comparing across different demographic characteristics. Understanding how people conceptualize CF, including their knowledge of risk and protective factors and their willingness to adopt preventative behaviors, is essential for developing effective interventions across the lifespan. This includes developing better and more targeted messaging that might promote proactive lifestyle behaviors earlier in the lifespan that are age‐ and culture‐appropriate, such as adequate nutrition, regular physical activity, and social connectivity.

This study aimed to explore public perceptions of CF, with a particular focus on differences across demographic characteristics. Specifically, it investigated awareness of CF, current health behaviors, and attitudes toward prevention. By examining beliefs about CF and readiness to engage in preventative practices, this research addresses a critical gap in the literature and offers a more inclusive understanding of CF prevention.

## 2 Methods

### 2.1 Study Design

The Preventing Cognitive Frailty cross‐sectional survey investigated individuals’ beliefs and knowledge regarding CF and behaviors for reducing or preventing CF. The survey was conducted across the United Kingdom between February and March 2025.

The survey was administered online via SurveyLab (https://www.surveylab.com) and took approximately 15–20 minutes to complete.

### 2.2 Recruitment

To ensure a diverse and representative sample, a variety of recruitment strategies were used. A total of 3,783 participants were recruited through Join Dementia Research (https://www.joindementiaresearch.nihr.ac.uk), a UK‐based platform supporting brain health and dementia‐related research. An additional 761 respondents were recruited through the researchers' networks and via online platforms, including social media.

In total, 4544 individuals initiated the survey. Eligibility criteria required participants to be aged 21 years or older and reside in the United Kingdom. There was no exclusion criterion based on cognitive or physical health status. Twenty‐four participants were excluded (younger than 21 or not living in the United Kingdom), resulting in a final sample of 4520 respondents. There were no missing responses to questions reported in the current analyses, given a 100% completion rate. However, there were errors with some demographic data, such as incomplete postcodes, which resulted in a smaller sample for some of the subgroup analyses.

Ethical approval for the study was obtained from the School of Social Sciences Ethics Committee at Heriot‐Watt University. Participants were provided with an information sheet at the start of the survey and gave informed consent before proceeding. A debrief sheet was presented upon survey completion. To support recruitment, participants had the option to enter a random draw for one of four £25 gift vouchers.

### 2.3 Survey

The survey assessed participants’ beliefs, knowledge, and behaviors related to CF (Supplementary Figure S1). First, participants were asked to complete their demographic characteristics, which included age in years, gender, ethnicity, postcode, education level, employment status, overall physical and mental health status, and current long‐term health conditions. Participants then completed questions about their familiarity with CF and whether CF could be reduced or prevented. Participants then rated on a scale from very important to not at all important how important 29 factors were for reducing or preventing CF. Across these same 29 factors, participants then rated how frequently they had engaged with them over the last month, on a scale from never to always. Finally, participants were told to imagine that 16 of these factors were proven to reduce or prevent CF and to rate how willing they were to increase participation in these activities on a scale from “does not encourage me” to “greatly encourages me.”

### 2.4 Data Analysis

Descriptive statistics were used to summarize responses. To calculate indices of multiple deprivation (IMD), participant postcodes were entered into the corresponding UK nation database to calculate an IMD score for each participant. Though most respondents provided their postcodes, some of those were only partial or were not included in the respective national deprivation databases, consequently not allowing us to calculate the IMD for those respondents.

To explore whether familiarity and preventability were influenced by demographic characteristics, *χ* ^2^ analyses were used. Post hoc tests were conducted by squaring the adjusted standardized residuals to produce individual *χ* ^2^ values for each comparison, with *p*‐values estimated using Microsoft Excel's *χ* ^2^ significance function. Excel Bonferroni‐adjusted significance thresholds were applied to account for multiple comparisons (Beasley and Schumacker 1995; MacDonald and Gardner 2000), following the method used in a previous survey (Niechcial et al. 2022; Vaportzis and Gow 2018).

To explore differences in the ranking of factors based on demographic characteristics, the number (percentage) of positive responses for each factor within each demographic subgroup (e.g., male and female; white and other ethnic group; IMD 1 through 5) were ranked from 1 to 29 for importance and currently doing and from 1 to 16 for willingness to do in the future. Spearman's rank correlation coefficients were then calculated to explore the similarity in the rankings between different demographic subgroups. Differences in factor ranks of 5 or more for importance and currently doing and 3 or more for willingness to do in the future were reported descriptively.

Logistic regression analyses were used to assess whether familiarity and preventability were associated with importance and current and future behaviors. Models included demographic covariates (age, gender, ethnicity, education, IMD, and health status) to examine potential moderating effects.

## 3 Results

### 3.1 Participants

Participants were 4520 adults aged 21–97 from all four countries of the United Kingdom. The sample had a mean (SD) age of 64.2 (12.1), was 70% male and 97% white, and 60% held an undergraduate or postgraduate qualification. Most respondents were retired (60%). Self‐rated health was generally favorable (physical health: 37% “very good” and 11% as “excellent”; mental health: 36% “very good” and 17% as “excellent”). The majority resided in England (91%), with most coming from the combined five least deprived areas of England, Wales, Scotland, and Northern Ireland based on postcodes. The demographic characteristics of the sample are displayed in Table 1. This spread of demographics represents the general skewed population of people interested in dementia research who are more likely to be enrolled in services like Join Dementia Research, such as being of white ethnicity, older adults, and caregivers of people with dementia (Marchant et al. 2025).

**TABLE 1:** Sample characteristics.

Variable	*N*	%
**Age group** 21–39 40–59 60–79 80–97	224 1044 2999 252	5% 23% 66% 6%
**Gender** Male Female Other Prefer not to say	3167 1341 9 3	70% 30% 0.2% 0.1%
**Ethnicity** White Asian Black Mixed Other Prefer not to say	4373 47 19 40 30 11	97% 1% 0.4% 1% 0.7% 0.2%
**Education** Postgraduate Undergraduate A‐level (or equivalent) GCSE/O Level (or equivalent) Apprenticeship Other No qualifications	1151 1571 898 510 59 250 79	26% 35% 20% 11% 1% 6% 2%
**Employment** Full‐time Part‐time Self‐employed Unable to work due to illness or disability Unemployed Stay‐at‐home spouse Student Volunteer Retired Other	777 484 262 64 34 48 31 103 2699 18	17% 11% 6% 1% 0.8% 1% 0.7% 2% 60% 0.4%
**Nation** England Wales Scotland Northern Ireland	3755 25 293 43	91% 0.6% 7% 1%
**Combined Indices of Multiple Deprivation deciles** 1 (most deprived) 2 3 4 5 6 7 8 9 10 (least deprived)	92 157 258 326 375 422 563 570 639 714	2% 4% 6% 8% 9% 10% 14% 14% 16% 17%
**Self‐rated physical health** Poor Fair Good Very good Excellent	140 686 1522 1679 493	3% 15% 34% 37% 11%
**Self‐rated mental health** Poor Fair Good Very good Excellent	111 646 1399 1603 760	3% 14% 31% 36% 17%

For group comparisons, demographic characteristics were consolidated where the original group sizes were too small to allow appropriate analyses. Information on the original and new groups can be seen in Supplementary Table S1.

### 3.2 Demographic Influence on Familiarity and Preventability

#### 3.2.1 Familiarity With Cognitive Frailty

Overall, 80% reported some degree of familiarity with CF (Table 2). Familiarity with CF differed by gender (*χ* ^2^ (4) = 57.94, *p* < 0.001) and self‐rated health (*χ* ^2^ (4) = 21.55, *p* < 0.001). *Post hoc* tests revealed that men were more likely to report being “very familiar” with CF than women (*p* < 0.0056). Participants with poor/fair health were less likely to report being “very familiar” with CF compared with those with very good/excellent health (*p* < 0.0056). The *χ* ^2^ tests of independence suggested no association between respondents’ age group, ethnicity, IMD, or education and their familiarity with CF (Table 3).

**TABLE 2:** Beliefs about cognitive frailty.

Beliefs about cognitive frailty	N	%
**Familiarity with cognitive frailty** Not familiar Slightly familiar Moderately familiar Very familiar Extremely familiar	915 1423 1499 547 136	20% 32% 33% 12% 3%
**Preventability of cognitive frailty** Yes, there are things people can do to prevent cognitive frailty No, there are no things people can do to prevent cognitive frailty Not sure	4093 69 359	91% 2% 8%

**TABLE 3:** *χ^2^* tests of independence for familiarity with cognitive frailty.

	Familiarity—*N* (%)			
Characteristics	Not familiar	Somewhat familiar	Very familiar	*χ^2^*
**Total**	915 (20%)	2922 (65%)	683 (15%)	
**Age group** Young adult 21–39 Middle‐aged 40–59 Young old 60–79 Old‐old 80+	44 (20%) 230 (22%) 584 (20%) 57 (23%)	144 (64%) 672 (64%) 1944 (65%) 161 (64%)	36 (16%) 142 (14%) 471 (16%) 34 (14%)	*χ^2^* (6) = 6.17 *p* = 0.404 *N* = 4,519
**Gender** Female Male Other	327 (24%) 585 (19%) 3 (25%)	885 (66%) 2032 (64%) 5 (42%)	129 (10%) 550 (17%) 4 (33%)	*χ^2^* (4) = 57.94 *p* < 0.001* *N* = 4,520
**Ethnicity** White Other ethnic group Prefer not to say	883 (20%) 28 (21%) 4 (36%)	2831 (65%) 86 (63%) 5 (46%)	659 (15%) 22 (16%) 2 (18%)	*χ^2^* (4) = 2.28 *p* = 0.685 *N* = 4,520
**Education** Lower Middle Higher Other	125 (21%) 182 (19%) 559 (21%) 49 (20%)	379 (64%) 641 (67%) 1735 (64%) 165 (66%)	85 (14%) 134 (14%) 428 (16%) 36 (14%)	*χ^2^* (6) = 4.10 *p* = 0.663 *N* = 4518
Deprivation (IMD**) 1 (highest) 2 3 4 5 6 7 8 9 10 (lowest)	21 (23%) 26 (17%) 56 (22%) 67 (21%) 108 (29%) 85 (20%) 105 (19%) 108 (19%) 130 (20%) 136 (19%)	56 (61%) 106 (68%) 170 (66%) 215 (66%) 216 (58%) 260 (62%) 373 (66%) 375 (66%) 412 (65%) 471 (66%)	15 (16%) 25 (16%) 32 (12%) 44 (14%) 51 (14%) 77 (18%) 85 (15%) 87 (15%) 97 (15%) 107 (15%)	*χ^2^* (18) = 26.50 *p* = 0.089 *N* = 4,116
**Health status** Poor/fair Good Very good/excellent	193 (23%) 312 (21%) 410 (20%)	534 (65%) 1003 (66%) 1385 (64%)	99 (12%) 207 (14%) 377 (17%)	*χ^2^* (4) = 21.55 *p* < 0.001* *N* = 4,520

*Note*: *denotes *χ* ^2^ tests significant at the *p* < 0.05 level. **Indices of Multiple Deprivation.

#### 3.2.2 Preventability of Cognitive Frailty

Overall, 91% of respondents believed that CF is preventable (Table 2). Beliefs about preventability differed by education (*χ* ^2^ (8) = 92.18, *p* < 0.001) and health status (*χ* ^2^ (4) = 89.28, *p* < 0.001). *Post hoc* tests revealed that those with lower education levels were less likely to believe CF was preventable than those with higher education (*p* < 0.0042). Participants reporting their health as poor/fair were less likely to believe CF is preventable and more likely to believe it is not preventable than those reporting their health as very good/excellent (*p* < 0.0056). The *χ^2^* tests of independence suggested no association between respondents’ age group, gender, ethnicity, and IMD and their beliefs about the preventability of CF (Table 4).

**TABLE 4:** *χ^2^* tests of independence for preventability of cognitive frailty.

	Preventability—*N* (%)			
Characteristics	Yes	No	Not sure	*χ^2^*
**Total**	4093 (90%)	69 (2%)	358 (8%)	
**Age group** Young adult 21–39 Middle‐aged 40–59 Young old 60–79 Old‐old 80+	208 (93%) 941 (90%) 2729 (91%) 214 (85%)	2 (1%) 18 (2%) 42 (1%) 7 (3%)	14 (6%) 85 (8%) 228 (8%) 31 (12%)	*χ^2^* (6) = 12.19 *p* = 0.058 *N* = 4,519
**Gender** Female Male Other	1192 (89%) 2890 (91%) 11 (92%)	28 (2%) 41 (1%) 0 (0%)	121 (9%) 236 (8%) 1 (8%)	*χ^2^* (4) = 7.59 *p* = 0.108 *N* = 4,520
**Ethnicity** White Other ethnic group Prefer not to say	3952 (90%) 130 (96%) 11 (100%)	68 (2%) 1 (0.7%) 0 (0%)	353 (8%) 5 (4%) 0 (0%)	*χ^2^* (4) = 5.35 *p* = 0.254 *N* = 4,520
**Education** Lower Middle Higher Other	483 (82%) 860 (90%) 2537 (93%) 211 (84%)	21 (4%) 17 (2%) 29 (1%) 2 (0.8%)	85 (14%) 80 (8%) 156 (6%) 37 (15%)	*χ^2^* (8) = 92.18 *p* < 0.001* *N* = 4,518
Deprivation (IMD**) 1 (highest) 2 3 4 5 6 7 8 9 10 (lowest)	77 (84%) 144 (92%) 229 (89%) 299 (92%) 339 (90%) 383 (91%) 509 (90%) 517 (91%) 568 (89%) 663 (93%)	3 (3%) 3 (2%) 4 (2%) 5 (2%) 5 (1%) 3 (0.7%) 11 (2%) 13 (2%) 14 (2%) 6 (0.8%)	12 (13%) 10 (6%) 25 (10%) 22 (7%) 31 (8%) 36 (9%) 43 (8%) 40 (7%) 57 (9%) 45 (6%)	*χ^2^* (18) = 20.32 *p* = 0.315 *N* = 4,116
**Health status** Poor/fair Good Very good/excellent	685 (83%) 1365 (90%) 2043 (94%)	25 (3%) 24 (2%) 20 (1%)	116 (14%) 133 (9%) 109 (5%)	*χ^2^* (4) = 89.28 *p* < 0.001* *N* = 4,520

*Note*: *denotes *χ* ^2^ tests significant at the *p* < 0.05 level. **Indices of Multiple Deprivation.

### 3.3 Importance of Prevention Factors

**FIGURE 1:**
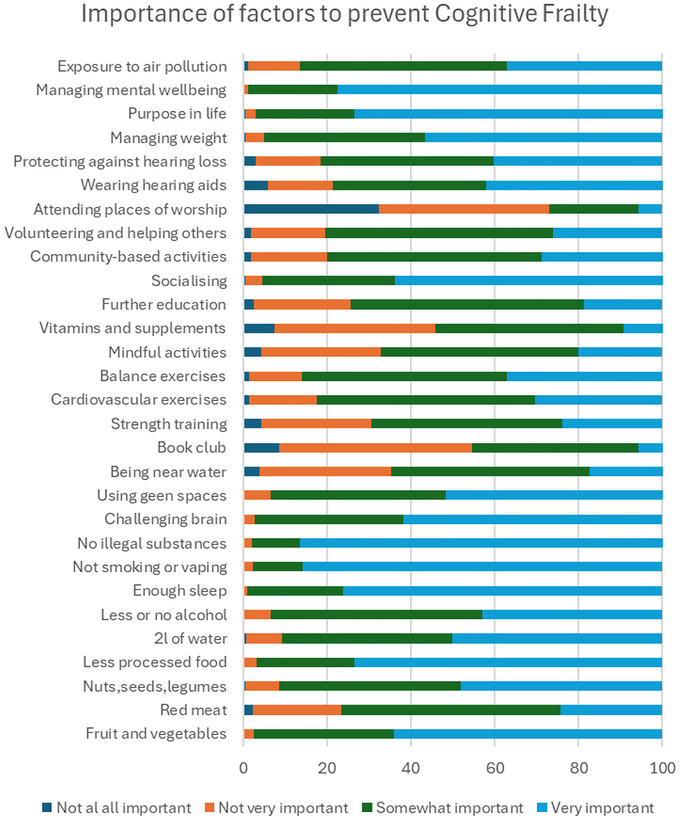
Percentage of responses for the importance of each preventable risk factor.

Of the 29 factors participants were presented with for reducing or preventing CF, those rated as very or somewhat important by respondents were not taking illegal substances, not smoking or vaping, managing mental well‐being, getting enough sleep, and eating less processed foods (Figure 1). These top five were most likely to be selected as important by men (*p <* 0.0042), older adults (*p* < 0.0031), those with better physical health (*p <* 0.0042), and those with higher education (*p <* 0.0031).

For age, the lowest correlation was observed between young adults and old‐old adults (Spearman's 𝜌 = 0.80). There were 12 (41%) factors that suggested being older or younger was associated with the factor being seen as important or not. Younger adults rated drinking the recommended water intake, drinking less or no alcohol, doing cardiovascular, strength, and balance exercises, and engaging in mindful activities as more important. Older adults thought that not taking illegal substances; having a purpose in life; volunteering and helping others, continuing with further education; wearing hearing aids; and eating less red meat were more important than for younger adults.

For ethnicity, there was a good correlation between ranks (Spearman's 𝜌 = 0.94), with 5 (17%) factors ranked more than five places differently between white and other ethnic groups. Managing weight and engaging in mindful activities were seen as more important by people from other ethnicities, whereas challenging your brain, protecting against hearing loss, and wearing hearing aids were ranked as more important by people from white ethnicities. For gender, there was excellent correlation between ranks (Spearman's 𝜌 = 0.98), with 2 (7%) factors ranked more than 5 places different between men and women. Doing cardiovascular exercise was seen as more important by women, and wearing hearing aids was seen as more important by men. For deprivation, the correlations between all groups were excellent (Spearman's 𝜌 > 0.97). For education level and physical health status, all correlations between groups were excellent (Spearman's 𝜌 > 0.97), with no differences between ranks of 5 or more places.

### 3.4 Behaviors Currently Engaging With

**FIGURE 2:**
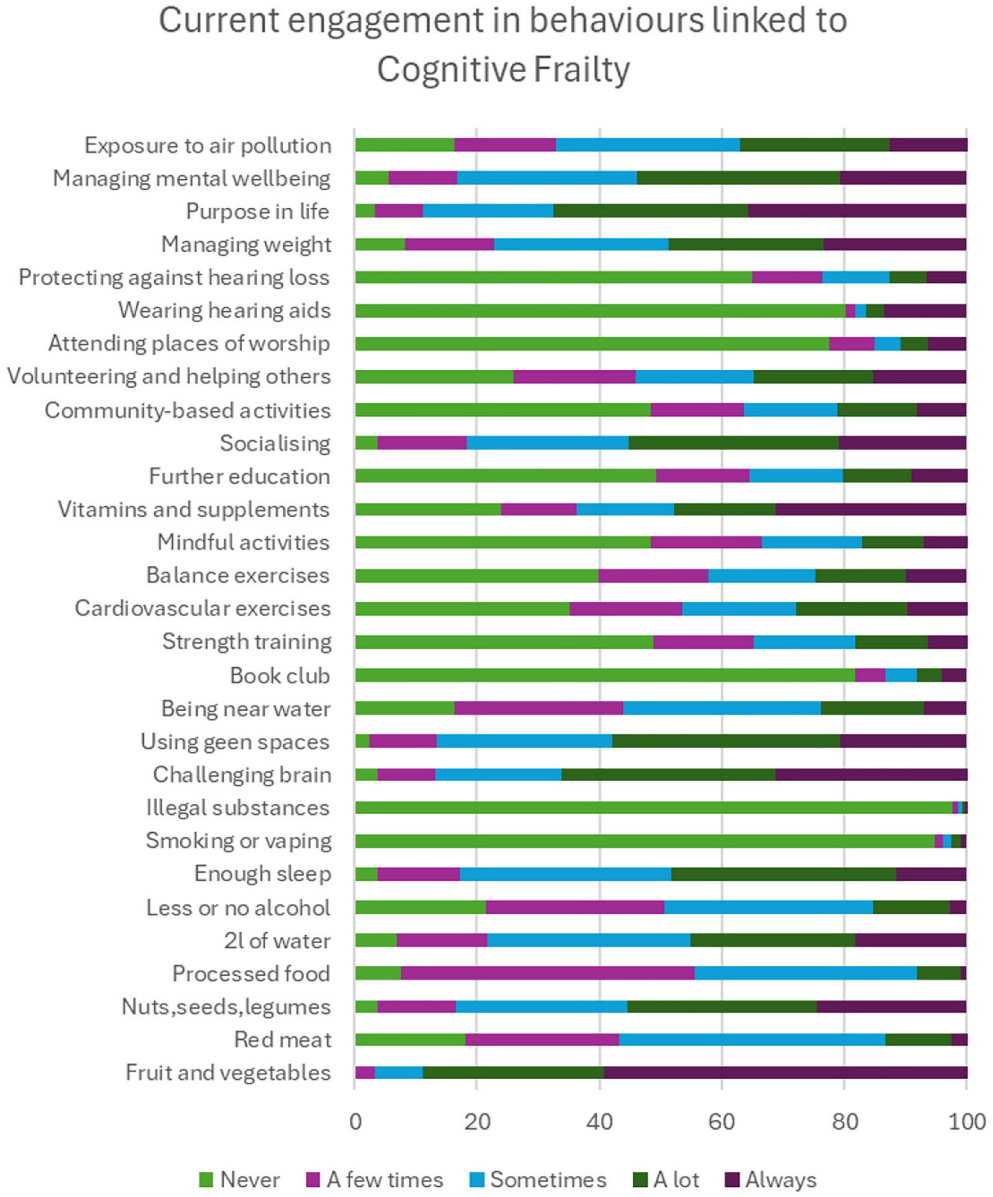
Percentage of responses for how frequently people were doing the behaviors.

The five behaviors respondents reported engaging in the most in at least a few times in the last month were: not taking illegal substances, not smoking or vaping, eating fruits and vegetables, having a purpose in life, and challenging the brain (Figure 2). These top five were most likely to be reported by men (*p <* 0.0033), older adults (*p* < 0.0025), white ethnicities (*p <* 0.0033), those living in less deprived areas (*p <* 0.0010), those with better physical health (*p <* 0.0033), and higher education (*p <* 0.0025).

For age, the lowest correlation was observed between young adults and old‐old adults (Spearman's 𝜌 = 0.79). There were 15 (52%) factors that suggested being older or younger was associated with being more likely to be currently engaging in the factor or behavior. Younger adults were more likely to be drinking the recommended water intake, socializing with family/friends, drinking less or no alcohol, continuing with further education, reducing red meat consumption, and doing cardiovascular and strength exercises. Older adults reported spending more time in green and blue spaces, eating nuts, seeds, and legumes, limiting exposure to air pollution, volunteering or helping others, eating less processed food, wearing hearing aids, and taking part in community‐based activities than younger adults.

For deprivation, the lowest correlation was observed between IMD1 and IMD5 (Spearman's 𝜌 = 0.94). There were 4 (14%) factors that suggested being more or less deprived were associated with being more likely to be currently engaging in the factor or behavior. People from more deprived areas (IMD1) were more likely to be managing mental well‐being and not drinking alcohol. People from less deprived areas (IMD5) were more likely to be spending time in green spaces and volunteering or helping others.

For ethnicity, there was a good correlation between ranks (Spearman's 𝜌 = 0.93), with 4 (14%) factors ranked more than 5 places differently between white and other ethnic groups. People from other ethnic groups were more likely to be drinking the recommended water intake and not drinking alcohol. Whereas people from white ethnicities were more likely to spend time in green and blue spaces. For gender, there was excellent correlation between ranks (Spearman's 𝜌 = 0.96), with 3 (10%) factors ranked more than 5 places differently between men and women. Women were more likely to be doing cardiovascular exercise, in line with their importance ranking, and men were more likely to be socializing with friends/family and doing balance exercises than women. For physical health, the lowest correlation was between poor/fair health and very good/excellent health (Spearman's 𝜌 = 0.93), 6 (21%) of the factors are associated with physical health status. People with poorer health were more likely to be drinking the recommended water intake, not drinking alcohol, and engaging in mindful activities and less likely to be getting enough sleep and doing cardiovascular and strength training than people with very good or excellent health. For education level, all correlations between groups were excellent (Spearman's 𝜌 > 0.98), with no differences between ranks of 5 or more places.

### 3.5 Willingness to Engage in Behaviors

**FIGURE 3:**
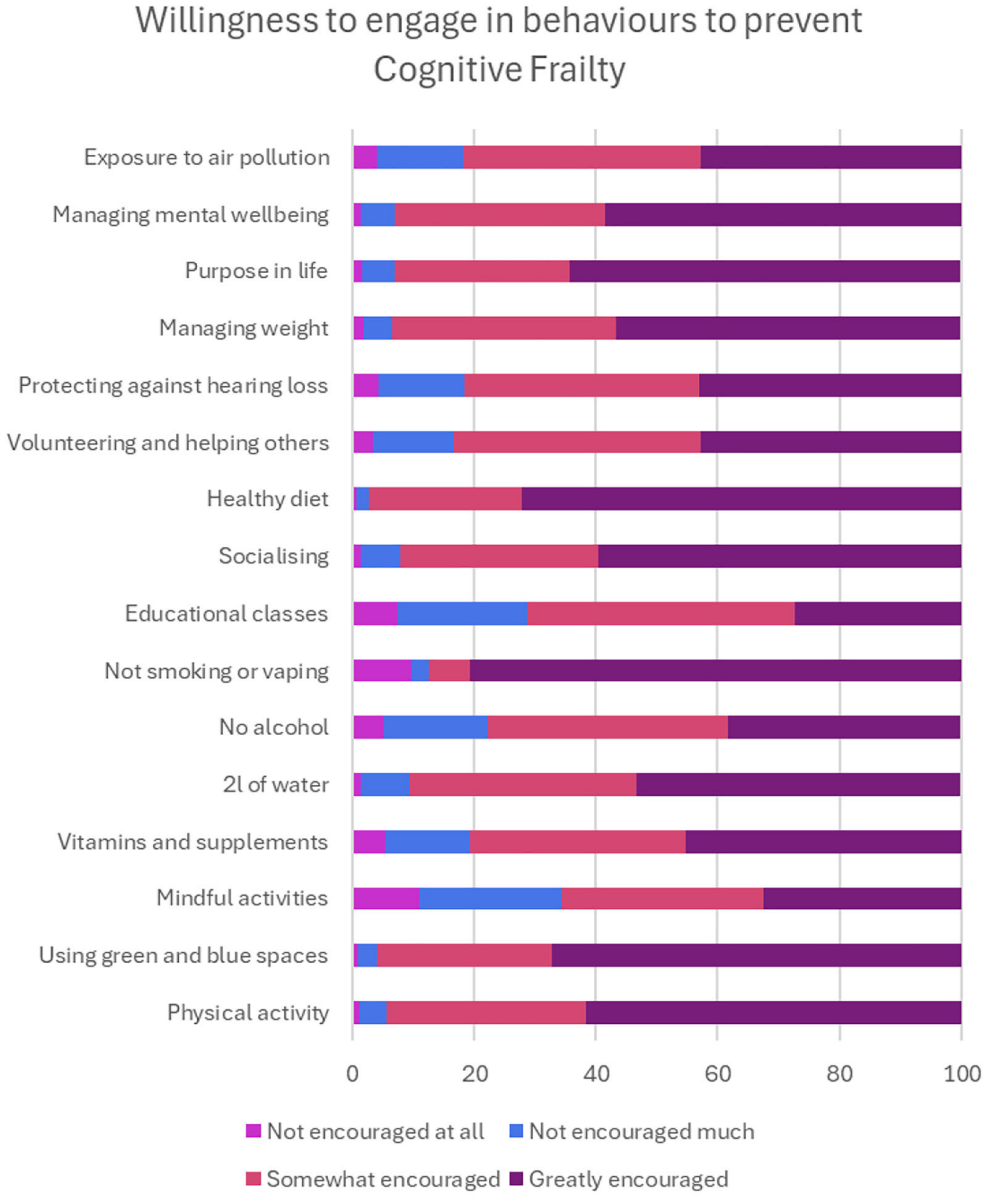
Percentage of responses for how willing people were to do the behaviors to prevent CF.

The five activities participants reported being somewhat or greatly encouraged to do the most in the future were not smoking, eating a healthy diet, using green and blue spaces, having a purpose in life, and physical activity (Figure 3). These behaviours were most likely to be reported as willing to do so in the future by men (*p <* 0.0042), older adults (*p* < 0.0031), white ethnicities (*p <* 0.0042), those living in less deprived areas (*p <* 0.0013), and those with better physical health (*p <* 0.0042).

For age, the lowest correlation was observed between young adults and old‐old adults (Spearman's 𝜌 = 0.60), with 3 (19%) factors associated with being more willing to engage in the factor or behavior. Younger adults were more willing to drink the recommended water intake, in line with what they were currently doing, and to take vitamins and supplements and less willing to prioritize having a purpose in life than older adults.

For deprivation, the lowest correlation was observed between IMD1 and IMD3, IMD4, and IMD5 (Spearman's 𝜌 = 0.89), suggesting IMD1 was most different from the other groups. There were 5 (31%) factors that suggested being more or less deprived were associated with being willing to engage in the factor or behavior in the future. People from more deprived areas (IMD1) were more willing to manage their mental well‐being and take vitamins or supplements but less willing to engage in physical activity, prioritize having a purpose in life, and limit exposure to air pollution.

For ethnicity, there was a lower correlation between ranks than for other comparisons (Spearman's 𝜌 = 0.84), with 6 (38%) factors ranked more than 3 places differently between white and other ethnic groups. People from other ethnic groups were more willing to drink the recommended water intake, take vitamins or supplements, and engage in mindful activities. Whereas people from white ethnicities were more willing to not smoke/vape, limit exposure to air pollution, and protect against hearing loss. For gender, there was excellent correlation between ranks (Spearman's 𝜌 = 0.96), with 2 (13%) factors ranked more than 3 places differently between men and women. Women were more willing to limit air pollution and were less willing to manage mental well‐being than men. For physical health, the lowest correlation was between poor/fair health and very good/excellent health (Spearman's 𝜌 = 0.86), with 4 (25%) factors ranked 3 or more places differently. People with poorer health were more willing to manage their mental well‐being and take vitamins and supplements and less willing to engage in physical activity and volunteer or help others than people with very good or excellent health.

For education level, all correlations between groups were good (Spearman's 𝜌 > 0.94), with 4 (25%) factors ranked 3 or more spaces different based on education level. People with lower education levels were more willing to identify a purpose in life and less likely to engage in physical activity, volunteer to help others, and protect against hearing loss than those with higher education levels.

### 3.6 Predictors of Behavioral Importance and Engagement

#### 3.6.1 Current Behaviors

Exploring the relationship between familiarity with CF and engagement in current behaviors, controlling for demographic characteristics, through logistic regression, it was found that increased familiarity with CF was associated with doing more: balance exercises, cardiovascular exercises, strength training, challenging the brain, doing community‐based activities, drinking more water, pursuing further education, spending more time in green and blue space, managing well‐being, managing weight, mindful activities, eating nuts, seeds, and legumes, having a purpose in life, volunteering, and increased socializing (Supplementary Table S2 for odds ratio and significance level).

Through logistic regression, the belief that CF could be prevented was associated with doing more: balance exercises, cardiovascular exercises, strength training, spending more time in blue space, managing well‐being, doing mindful activities, having a purpose in life, managing weight, eating nuts, seeds, and legumes; volunteering; increased socializing; getting enough sleep; and reducing air pollution (Supplementary Table S3 for odds ratio and significance level).

In almost all cases, when a participant thought a factor was important for reducing or preventing CF, they were more likely to report that they were currently doing that behavior. Exceptions were eating fruits and vegetables, eating processed foods, and taking illegal substances, highlighting these as particularly difficult behaviors to engage in and change (Supplementary Table S4 for odds ratio and significance level).

##### 3.6.1.1 Demographic Control Variables

There was a significant interaction for age across almost all current behaviors toward CF prevention, with people being more likely to do the following health behaviors as they got older: attend places of worship, do balance exercises, attend a book club, challenge the brain, participate in community activities, drink alcohol, get enough sleep, be exposed to air pollution, spend time in green and blue spaces, wear hearing aids, protect against hearing loss, manage weight, take part in mindful activities, eat nuts, seeds and legumes, and volunteer. The following behaviors were more likely to be done by younger participants: taking part in further education, managing wellbeing, strength training, cardiovascular exercise, eating processed foods, smoking/vaping, and taking illegal substances.

There were significant interactions for gender for many of the behaviors, with men being more likely to engage in well‐being and community‐based activities (attending places of worship, having a purpose in life, doing mindfulness exercises, attending book clubs, socializing, and volunteering), doing balance exercises; eating nuts and seeds and taking vitamins and supplements; and doing further education. Women were more likely to be drinking alcohol and taking illegal substances, eating red meat and processed food, doing cardiovascular exercise, protecting against hearing loss, and wearing hearing aids. There were no differences between other behaviors for gender.

For ethnicity, white respondents were more likely to be challenging the brain, drinking alcohol, being in green and blue spaces, and eating processed foods. Those from other ethnic groups were more likely to be attending places of worship, going to book clubs, taking part in further education, and doing mindfulness activities.

Those from more deprived areas were more likely to be taking illegal substances and smoking/vaping, whereas people living in less deprived areas were more likely to be participating in balance and strength exercises, attending book clubs, engaging with community activities, drinking alcohol, and eating red meat.

There was an interaction for education level, with higher education levels associated with doing balance, cardiovascular, and strength training; engaging in community activities (places of worship, book club, volunteering); doing further education; mindfulness activities; better sleep; spending time in green spaces; and eating more nuts and seeds. Lower education levels were associated with eating more red meat and smoking/vaping.

As participants self‐rated their own health as better, they were more likely to be doing balance, cardiovascular, and strength training; engaging in community activities (book club, social activities, and volunteering); having better sleep; managing their weight; eating more nuts, seeds and legumes; having a purpose in life; spending time in green and blue spaces; and drinking more alcohol. Those who self‐rated their health as poorer were more likely to be wearing hearing aids, be smoking/vaping, and be taking vitamins and supplements.

#### 3.6.2 Future Behaviors

When looking at participants' willingness to engage in these behaviors in the future, those who were more familiar with CF were more willing to engage with drinking less alcohol, taking educational classes, limiting air pollution exposure, spending time in green and blue spaces, eating a healthy diet, managing mental well‐being, managing weight, doing mindful activities, engaging in physical activity, protecting against hearing loss, having a purpose in life, not smoking or vaping, socializing with friends or family, and volunteering (Supplementary Table S5 for odds ratio and significance level).

Those who thought CF could be prevented were more willing to engage in all the suggested behaviors in the future (Supplementary Table S6 for odds ratio and significance level). Similarly, for every behavior, if a participant thought it was important for reducing or preventing CF, then they were statistically more likely to be willing to engage in doing the behavior in the future (Supplementary Table S7 for odds ratio and significance level).

##### 3.6.2.1 Demographic Control Variables

There was a significant interaction for age, with younger participants being more willing to engage with the following behaviors: drinking at least 2 liters of water, reducing alcohol consumption, reducing exposure to air pollution, engaging in mindful activities, protecting against hearing loss, and taking vitamins and supplements. Across all behaviors, women were less willing to engage with future behaviors than men. There were very few differences in willingness to engage in future behaviors based on ethnicity, except for people from other ethnic groups being more willing to engage in mindful activities. There were only a few interactions between living in less deprived areas and willingness to engage in behaviors, which were for reducing exposure to air pollution, engaging in physical activity, protecting against hearing loss, having a purpose in life, and not smoking or vaping. Similarly, participants with higher education reported a greater willingness to engage with future behaviors, which was only significantly different for engaging in further education, taking part in mindful activities, and volunteering.

Self‐rated current health had the most observed impact on participants' willingness to engage in future health behaviors, with associations between higher health status and reducing alcohol consumption, stopping smoking or vaping, taking part in education, reducing exposure to air pollution, accessing green and blue spaces, eating a healthy diet, managing weight, taking part in physical activity, preventing hearing loss, having a purpose in life, socializing with friends and family, and volunteering. However, drinking 2 liters of water, managing mental well‐being, taking part in mindful activities, and taking vitamins and supplements were not.

### 3.7 Discussion

Public perceptions of CF, particularly awareness, beliefs, current health behaviors, attitudes toward prevention, and perceived barriers or motivators, have not previously been adequately studied across demographic characteristics. This current UK‐wide survey, therefore, provided valuable insight to support evidence‐based development of health promotion strategies, interventions, and educational resources that effectively target appropriate groups regarding CF prevention.

Overall, most participants reported having at least some familiarity with CF (80%) and believed it could be prevented (91%). Familiarity with CF was higher among males and those in very good health, while belief in the preventability of CF was more common among those with higher education and very good health. These results are surprising given that the term “Cognitive Frailty” is relatively new and is not often used in the public domain. As the proportion of older adults in the population increases, physical frailty continues to be a common geriatric syndrome among older adults in the community, highlighting the greater awareness of its related risk factors in this demographic (Clegg et al. 2013; Ofori‐Asenso et al. 2019). Assessment of risk factors of CF in older adults are typically integrated into the comprehensive geriatric assessment, such as mobility, nutritional status, sleep behavior, and physical activity (Pilotto et al. 2020). Such exposure might have influenced the reported knowledge of CF in the older adult population. However, it is possible that people confused the term “CF” with similar but distinct terms, such as “subjective cognitive complaints,” “cognitive decline,” or “cognitive impairment,” and were conflating their understanding of “CF” with these terms. In addition, as many participants were recruited through Join Dementia Research, our sample could have more of an interest in this area, reflecting higher awareness of CF, which may not be seen in the wider population.

Not taking illegal substances, not smoking/vaping, and managing mental well‐being were rated as the most important factors for preventing CF, while attending book clubs and places of worship were the least important. These perceptions somewhat align with what we currently know about dementia prevention. The Lancet Commission highlighted that reducing smoking, managing depression, and reducing social isolation are associated with a reduced risk of dementia (Livingston et al. 2024). There is growing evidence for CF prevention through non‐pharmacological interventions, including the impact of physical exercise alongside cognitive training, eating a healthy diet, and sleep routine maintenance (Ruan et al. 2015; Bortone et al. 2021; Corral‐Pérez et al. 2023; Sugimoto et al. 2022). These interventions can lead to improvements in CF outcomes, as well as benefits for mental health, social engagement, and quality of life (Gajdosova et al. 2023; Szychowska and Drygas 2022; Li et al. 2022). The few preventative strategies for CF among participants indicate that, although these measures are demonstrated in research, they are not yet known among the wider population and highlight the need for public health campaigns and messaging.

When looking at current behaviors for CF, respondents most often reported eating a healthy diet, such as fruits, vegetables, nuts, seeds, and legumes, and taking vitamins and supplements. People were also focused on having a purpose in life and challenging their brains. The factors people were doing the least were attending book clubs places of worship and taking part in strength training. In terms of healthy diets, having a purpose in life, and challenging the brain, engagement to prevent CF was highest among the young‐old age group, males, white ethnicity, and more educated respondents. Other studies have also found that men, participants aged 60–69, and those with higher educational levels have significantly lower prevalence of reversible CF than women, other age groups, and lower education, respectively (Ruan et al. 2020; Qiu et al. 2022). Therefore, it is unsurprising that those at the lowest risk of CF may be engaging the most in activities related to healthy brain aging.

While there were large differences in the current behaviors participants were engaging in, there were fewer differences in what participants would be willing to engage with in the future if they had knowledge that it would reduce or prevent CF. Generally, younger adults were open to engaging with new behaviors in the future, though they were currently doing fewer of those behaviors than older adults, such as drinking less alcohol, reducing exposure to air pollution, protecting against hearing loss, and engaging in mindful activities. Exploring engagement with behavior change in the United Kingdom during COVID, Anyanwu et al. (2022) found that those aged 18–34 years made the most efforts to change their behavior and that there was a trend for behavior change efforts to become less common with increasing age. Differences based on deprivation seemed to be associated with factors that were influenced by access to supporting services, reducing exposure to air pollution, engaging in physical activity, and not smoking or vaping. These access barriers were reflected in an NHS Digital (2023) report, which found that being overweight, a current smoker, and physically inactive were more common and harder to change for people living in more deprived areas.

Self‐rated health influenced the willingness to engage in the most future health behaviors to prevent CF. Respondents with poorer health reported lower willingness to adopt preventative strategies. Evidence suggests that having long‐term conditions and poorer health makes behavior change significantly harder due to increased mental health challenges like anxiety and depression, reduced self‐esteem, social isolation, fatigue, and the physical and emotional burden of daily symptom management (Kelly and Barker, 2016). These factors, combined with environmental, social, and economic barriers, create a cycle where poor health makes it difficult to adopt healthier behaviors, which in turn can worsen health. This suggests that awareness campaigns should be paired with practical, accessible support to enable behavior change in those with poorer health. A key challenge is how to increase awareness of behaviors that reduce CF risk among those most at risk and how to support their engagement with health behavior change.

#### 3.7.1 Strengths and Limitations

This study benefited from exploring public perceptions of CF, still a relatively new concept and one with less penetration than physical or cognitive impairment alone, in a large sample, drawn from the four nations of the United Kingdom and across the adult lifespan. The survey focused not only on understanding of CF but also on behavior change and reported willingness to engage in specific preventative behaviors.

Over 4500 responses were received; however, the sample had more men, white, degree‐educated and retired people, and were older and less deprived than the wider UK population, suggesting the findings may not be representative of and generalizable to the wider target population. In addition, 84% of the sample was recruited via Join Dementia Research, which may explain the skewed sample. People who actively engage in dementia research may have different opinions and motivators, which may also reduce the generalizability of our findings to the wider population (Marchant et al. 2025). However, we did control for these demographic characteristics in our regression analyses.

For deprivation, we have used each UK nation's Decile scale and combined them into the same groupings. While deprivation indices are not a continuous outcome, it is unlikely that a score of 2 in one UK nation is the same as another, so this may not be a valid way of measuring and controlling for deprivation status across all four UK nations. Sample sizes for Wales and Northern Ireland were smaller than the proportion living there, limiting subgroup comparisons, though their inclusion was deemed important for representativeness.

We have conducted multiple post‐hoc analyses as part of this study. Though all analyses were p‐adjusted, the findings should be interpreted with caution. Additionally, some subgroups had small sample sizes, meaning those may not represent the population from which they were drawn and that any group differences should not be considered definitive.

Another limitation of this work is that the survey questions have not been validated. For example, participants reported high familiarity with CF, though given the term includes “cognitive,” it may mean people believed the term was referring to cognitive decline, mild cognitive impairment, or dementia rather than the co‐occurrence of cognitive and physical impairments. The finding was unexpected, given that CF is a relatively new term and is not frequently used. The current data did not allow a more in‐depth exploration of stated versus actual knowledge. However, after the early questions, CF was always clearly defined as “a condition where a person experiences both physical weakness (frailty) and cognitive difficulties with memory or thinking, but without having dementia. It increases the risk of further health issues and cognitive decline.” The results could be best interpreted in terms of cognitive impairment.

### 3.8 Future Research Recommendations

Future interventions to promote healthy aging should be tailored to the needs and capacities of specific populations. It is important to understand the target population the intervention aims to support and identify what they already know, what they are currently doing, and what they would be willing/able to do in the future. This ensures that the aims of the intervention align with the needs and capabilities of the target group.

These findings highlighted that those from underserved groups are currently less likely to engage with health behaviors that prevent CF but would be willing to engage in those in the future. Therefore, there is a gap where existing services are not meeting their needs or offering options they are willing to engage with. Public health campaigns raising awareness of preventative behaviors are a crucial first step, followed by tailored interventions that address barriers, access, and motivation.

## 4 Conclusion

Respondents reported relatively high awareness of CF and endorsed several preventative strategies. Adults aged 40–59, who were male, white, and highly educated, were more likely to engage in behaviors to prevent CF and be willing to engage in these behaviors in the future. Those from other ethnic groups, less educated, and who report poorer health outcomes were currently less likely to be engaging with healthy behaviors that prevent CF, but most expressed some willingness to engage in these in the future. These findings underscore the potential for targeted interventions and public health campaigns to increase awareness and support engagement with preventative behaviors across all groups, thus promoting healthy aging and reducing the risk of CF.

## Author Contributions

Conceptualization: Shaimaa Elhag, Tasmin Rookes, Malwina Niechcial, Mohaddeseh Ziyachi, Ruoyu Wang, Millennium Iyobuchiebomie, and Alan Gow. Methodology: Shaimaa Elhag, Tasmin Rookes, Malwina Niechcial, and Alan Gow. Investigation: Shaimaa Elhag, Tasmin Rookes, and Malwina Niechcial. Formal analysis: Shaimaa Elhag, Tasmin Rookes, and Malwina Niechcial. Supervision: Alan Gow. Funding acquisition: Shaimaa Elhag, Tasmin Rookes, Malwina Niechcial, Mohaddeseh Ziyachi, Ruoyu Wang, Millennium Iyobuchiebomie, and Alan Gow. Writing – original draft: Shaimaa Elhag, Tasmin Rookes, Malwina Niechcial, and Ruoyu Wang. Writing – review and editing: Mohaddeseh Ziyachi, Millennium Iyobuchiebomie, and Alan Gow

## Funding

This work was funded as a Pump Priming award from the Cognitive Frailty Interdisciplinary Network (CFIN), which was funded by the BBSRC/MRC award (BB/W018322/1).

## Ethics Statement

Ethical approval for the study was obtained from the School of Social Sciences Ethics Committee at Heriot‐Watt University.

Consent:

Participants were provided with an information sheet at the start of the survey and gave informed consent before proceeding. A debrief sheet was presented upon survey completion.

## Conflicts of Interest

The authors declare no conflicts of interest.

## Supporting information




**Supplementary Materials**: brb371307‐sup‐0001‐Figure1.docx

**Supplementary Tables**: brb371307‐sup‐0002‐Tables.docx

## Data Availability

Data is available upon reasonable request to the corresponding author.
