# Supplementary material for: Preventing Cognitive Frailty: What People Know and What They Are Willing to Change
Source: Brain Behav. 2026 Mar 23;16(3):e71307. doi: 10.1002/brb3.71307 (PMC13093823; doi:10.1002/brb3.71307)
Supplement: Supplementary file 1 — Supplementary Materials: brb371307‐sup‐0001‐Figure1.docx [file BRB3-16-e71307-s002.docx]

Screening Questionnaire

### Screening Question 1: Age Verification

**Question:**
"Are you 21 years of age or older?"

**Answer Options:**

1. Yes
2. No

**Logic:**

- If the participant selects "Yes," they proceed to the next question.
- If the participant selects "No," they are exited from the survey:
  "Thank you for your interest in this study. Unfortunately, you are not eligible to participate as the study is restricted to individuals aged 21 or older."

### Screening Question 2: Residency Verification

**Question:**
"Do you currently live in the United Kingdom?"

**Answer Options:**

1. Yes
2. No

**Logic:**

- If the participant selects "Yes," they proceed to the main survey.
- If the participant selects "No," they are exited from the survey:

# CFIN Questionnaire

We are asking people of all ages to complete this survey to understand what they currently know about links between the health of their body and brain, what activities they currently do to support their physical and brain health, and what they would be willing and able to do in the future to prevent or reduce changes in these.

The first group of questions will ask some background information about yourself.

## Demographics:

1. What is your age, in years?
2. What is your gender?

- Male (1)
- Female (2)
- Other (please specify) (3)
- Prefer not to say (4)

1. What is your ethnicity?

- White (1)
  - English/Welsh/Scottish/Northern Irish/British
  - Irish
  - Gypsy or Irish Traveller
  - Any other white background ………………
- Mixed/multiple ethnic group (2)
  - White and black Caribbean
  - White and black African
  - White and Asian
  - Any other mixed/multiple ethnic background ………….
- Asian/Asian British (3)
  - Indian
  - Pakistani
  - Bangladeshi
  - Chinese
  - Any other Asian background ………….
- Black/African/Black Caribbean/Black British (4)
  - African
  - Caribbean
  - Any other Black/African/Caribbean background ………….
- Other ethnic group (5)
  - Arab
  - Any other ethnic group ………….
- Prefer not to say (6)

1. What is your postcode?
2. What is your highest level of education?

- No qualifications (1)
- GCSE/O Level or equivalent (e.g., National 4/5, Standard Grades) (2)
- Apprenticeship (3)
- A Level, diploma, or NVQ level 3 (e.g., Scottish Highers, Advanced Highers) (4)
- Undergraduate degree (e.g., bachelor's degree or Honours degree) (5)
- Postgraduate degree (e.g., master's degree, PhD) (6)
- Other (please specify) ………………. (7)

1. What is your current employment status?

- Full-time employed (1)
- Part-time employed (2)
- Self-employed (3)
- Unable to work due to illness/exempt through disability (4)
- Unemployed (5)
- Stay-at-home spouse (6)
- Student (7)
- Volunteer (8)
- Retired (9)
- Other (10)

1. Thinking about the last two weeks how would you rate your overall physical health?

- Excellent (5)
- Very good (4)
- Good (3)
- Fair (2)
- Poor (1)

1. Thinking about the last two weeks how would you rate your overall mental health?

- Excellent (5)
- Very good (4)
- Good (3)
- Fair (2)
- Poor (1)

1. Do you currently have any diagnosed long-term health conditions? (such as hypertension, diabetes, high cholesterol, COPD, asthma, kidney disease, liver disease, anaemia, cancer, arthritis, epilepsy, dementia, depression, anxiety)

Yes (2) / No (1)

If yes which of these conditions, do you have.

Cognitive Frailty:

1. How familiar are you with the term cognitive frailty?

- Not at all familiar (1)
- Slightly familiar (2)
- Moderately familiar (3)
- Very Familiar (4)
- Extremely familiar (5)

Cognitive frailty is a condition where a person experiences both physical weakness (frailty) and cognitive difficulties with memory or thinking, but without having dementia. It increases the risk of further health issues and cognitive decline.

1. Our lifestyle choices can influence how healthy we remain as we age. Additionally, some health outcomes may be affected by our genes.

I think that the changes we experience in our physical health and memory or thinking skills as we age are (choose what you think might be most likely)

- Entirely determined by our genes (5)
- Mostly determined by our genes (4)
- Probably about half determined by our genes and half determined by our lifestyle (3)
- Mostly determined by our lifestyle (2)
- Entirely determined by our lifestyle (1)

1. Do you think cognitive frailty (that’s experiencing both physical weakness and memory/thinking problems) can be prevented or reduced through healthy lifestyle changes?

- Yes – there are things people can do to prevent and/or reduced cognitive frailty (1)
- No- I do not think there are things people can do to prevent and/or reduced cognitive frailty (2)
- Not sure (3)

## Current knowledge:

1. How important do you believe each of the following factors is for reducing or preventing cognitive frailty (that’s experiencing both physical weakness and memory/thinking problems)?

|  | Very important(4) | Somewhat important (3) | Not very important(2) | Not at all important(1) |
| --- | --- | --- | --- | --- |
| Eating fruit and vegetables |  |  |  |  |
| Eating less red meat |  |  |  |  |
| Eating nuts, seeds and legumes |  |  |  |  |
| Eating less processed foods |  |  |  |  |
| Drinking recommended water intake (2 litres) |  |  |  |  |
| Drinking less or no alcohol |  |  |  |  |
| Getting enough sleep |  |  |  |  |
| Not smoking/vaping |  |  |  |  |
| Not taking illegal substances |  |  |  |  |
| Challenging your brain (e.g., brain training games, puzzles) |  |  |  |  |
| Spending time in green spaces (e.g., parks, woodland) |  |  |  |  |
| Spending time near water |  |  |  |  |
| Participating in a book club |  |  |  |  |
| Doing strength training (e.g., lifting weights) |  |  |  |  |
| Doing cardiovascular exercise (e.g., running) |  |  |  |  |
| Doing balance exercises (e.g., Tai Chi, Yoga) |  |  |  |  |
| Engaging in mindful activities (e.g., meditation, prayer) |  |  |  |  |
| Taking vitamins or supplements |  |  |  |  |
| Continuing with further education (e.g., computer training, language classes) |  |  |  |  |
| Socialising with friends and/or family (e.g., coffee mornings) |  |  |  |  |
| Taking part in community-based activities (e.g., gardening, art classes) |  |  |  |  |
| Volunteering or helping others |  |  |  |  |
| Attending places of worship |  |  |  |  |
| Wearing hearing aids |  |  |  |  |
| Protecting against hearing loss (e.g., earplugs in noisy areas) |  |  |  |  |
| Managing weight |  |  |  |  |
| Having a purpose in life |  |  |  |  |
| Managing mental wellbeing (e.g., stress, depression) |  |  |  |  |
| Limiting exposure to air pollution |  |  |  |  |

Other (specify) ………………………….

## Current behaviours

1. Below is a list of activities that might be related to our health and wellbeing, whether protective or damaging. Over the last month, how often have you done the following behaviours?

|  | Never (1) | A few times (2) | Sometimes (3) | A lot (4) | Always (5) |
| --- | --- | --- | --- | --- | --- |
| Eating fruit and vegetables |  |  |  |  |  |
| Eating red meat |  |  |  |  |  |
| Eating nuts, seeds, and legumes |  |  |  |  |  |
| Eating processed foods |  |  |  |  |  |
| Drinking recommended water intake (2 litres) |  |  |  |  |  |
| Drinking alcohol |  |  |  |  |  |
| Getting enough sleep |  |  |  |  |  |
| Smoking/vaping |  |  |  |  |  |
| Taking illegal substances |  |  |  |  |  |
| Challenging your brain (e.g., brain training game, puzzles) or similar activities |  |  |  |  |  |
| Spending time in green spaces (e.g., parks, woodland) |  |  |  |  |  |
| Spending time near bodies of water |  |  |  |  |  |
| Participating in a book club |  |  |  |  |  |
| Doing strength training (e.g., lifting weights) |  |  |  |  |  |
| Doing cardiovascular (e.g., running) |  |  |  |  |  |
| Doing balance exercises (e.g., Tai Chi, Yoga) |  |  |  |  |  |
| Engaging in mindful activities (e.g., meditation, prayers) |  |  |  |  |  |
| Taking vitamins or supplements |  |  |  |  |  |
| Continuing with further education (e.g., computer training, language classes) |  |  |  |  |  |
| Socialising with friends and/or family (e.g., coffee mornings) |  |  |  |  |  |
| Taking part in community-based activities (e.g., gardening, art classes) |  |  |  |  |  |
| Volunteering or helping others |  |  |  |  |  |
| Attending places of worship |  |  |  |  |  |
| Wearing hearing aids |  |  |  |  |  |
| Protecting against hearing loss (e.g., earplugs in noisy areas) |  |  |  |  |  |
| Managing weight |  |  |  |  |  |
| Having a purpose in life |  |  |  |  |  |
| Managing mental wellbeing (e.g., stress, depression) |  |  |  |  |  |
| Exposed to air pollution |  |  |  |  |  |

Other (specify) …

## Future behaviours

1. Imagine you were told that each of the activities listed below has been proven to help prevent or reduce cognitive frailty. How would this information affect your willingness to start or increase your participation in these activities?

|  | Greatly encourages me (4) | Somewhat encourages me (3) | Does not encourage me much (2) | Does not encourage me at all (1) |
| --- | --- | --- | --- | --- |
| Engaging in physical activity |  |  |  |  |
| Spending time in or near green and blue spaces (e.g., parks, lakes) |  |  |  |  |
| Engaging in mindful activities (e.g., meditation, prayer) |  |  |  |  |
| Taking vitamins or supplements |  |  |  |  |
| Drinking recommended water intake (2 litres) |  |  |  |  |
| Drinking less or no alcohol |  |  |  |  |
| Not smoking /vaping |  |  |  |  |
| Taking educational classes |  |  |  |  |
| Socialising with friends and/or family |  |  |  |  |
| Eating a healthy diet |  |  |  |  |
| Volunteering or helping others |  |  |  |  |
| Protecting against hearing loss (e.g., listening to music in headphones at low level) |  |  |  |  |
| Managing weight |  |  |  |  |
| Having a purpose in life |  |  |  |  |
| Managing mental wellbeing (e.g., stress, depression) |  |  |  |  |
| Limiting exposure to air pollution |  |  |  |  |

1. If a safe and effective pharmaceutical intervention (such as a pill) were available to prevent or reduce cognitive frailty, how likely would you be to consider taking it?

- Very likely (5)
- Likely (4)
- Neutral/not sure (3)
- Unlikely (2)
- Very unlikely (1)

## What factors or concerns would influence your decision to take a pharmaceutical intervention (such as a pill) to prevent or reduce cognitive frailty? (e.g., safety, cost, effectiveness, or other factors—please specify)

## Barriers and Enablers

1. When considering activities that help prevent or reduce cognitive frailty, are there any factors that act as barriers to your participation in these activities?
   (e.g., lack of local services, services being expensive, cost of healthy food, doing what family members tell you to do, lack of social support etc.)
2. Are there any factors that make it easier or enable you to engage in these activities? (e.g., having family and friends you can rely on, being part of a social group, living in supported living, access to a local gym or swimming pool, having more local parks etc.)
3. How much would each of the following situations motivate you to participate in activities to reduce or reduced cognitive frailty (that’s experiencing both physical weakness and memory/thinking problems)?

|  | Motivates me a lot (3) | Motivates me a bit (2) | Does not motivate me (1) |
| --- | --- | --- | --- |
| Caring for, or observing someone with cognitive frailty |  |  |  |
| Having a friend to exercise with |  |  |  |
| Seeing or hearing about it in media |  |  |  |
| Receiving a recommendation from a doctor or other healthcare provider |  |  |  |
| Reading an article or blog about how certain activity helps |  |  |  |
| Feeling that your own physical health and cognitive health isn't the same as it used to be |  |  |  |

1. Which of the following sources would you or do you use to seek advice on reducing or reversing physical and cognitive health and preventing cognitive frailty (that’s experiencing both physical weakness and memory/thinking problems)?

|  | Would not use (1) | Currently use (2) | Would consider using (3) |
| --- | --- | --- | --- |
| Family/friends |  |  |  |
| Doctors or healthcare provider |  |  |  |
| Books/journals |  |  |  |
| Radio/media/tv |  |  |  |
| Charities or helplines |  |  |  |
| Pharmacist |  |  |  |
| Dietitian |  |  |  |
| Physiotherapist |  |  |  |
| Internet webpages about topic |  |  |  |
| Other (specify) |  |  |  |

Thank you for taking the time to complete this survey. We will analyse the responses to identify any differences in the types of behaviours people are currently engaging in or are willing to adopt in the future to prevent cognitive frailty, particularly in relation to different demographic characteristics, especially age group.
