# Supplementary material for: Preventing Cognitive Frailty: What People Know and What They Are Willing to Change
Source: Brain Behav. 2026 Mar 23;16(3):e71307. doi: 10.1002/brb3.71307 (PMC13093823; doi:10.1002/brb3.71307)
Supplement: Supplementary file 2 — Supplementary Tables: brb371307‐sup‐0002‐Tables.docx [file BRB3-16-e71307-s001.docx]

# Supplementary Table 1: Consolidation of categories for analysis.

| Demographic | Original Groups | New Groups |
| --- | --- | --- |
| Age | Continuous Variable | Young Adults (21-39 years)  Middle-Aged (40-59 years)  Young-Old (60-79 years)  Old-Old (80+ years) |
| Education | No qualifications | Lower Education |
|  | GCSE and O Level or equivalent |  |
|  | A-Level or equivalent | Middle Education |
|  | Apprenticeship |  |
|  | Undergraduate and Postgraduate qualifications | Higher Education |
|  | Other | Other |
| Ethnicity | White | White |
|  | Asian, Black, Mixed and Other | Other ethnic group |
|  | Prefer not to say | Prefer not to say |
| Gender | Male | Male |
|  | Female | Female |
|  | Other and Prefer not to say | Other |
| Physical Health | Poor | Poor/Fair |
|  | Fair |  |
|  | Good | Good |
|  | Very Good | Very Good/Excellent |
|  | Excellent |  |
| Prevention | Yes, preventable | Yes, preventable |
|  | No, not preventable | No, not preventable/Not Sure |
|  | Not Sure |  |
| Familiarity | Extremely Familiar | Familiar |
|  | Very Familiar |  |
|  | Moderately Familiar |  |
|  | Slightly Familiar |  |
|  | Not Familiar | Not Familiar |
| Importance of Factors | Not at all important | Not important |
|  | Not very important |  |
|  | Somewhat important | Important |
|  | Very important |  |
| Current behaviours engagement | Never | Do not do |
|  | A few times | Do |
|  | Sometimes |  |
|  | A lot |  |
|  | Always |  |
| Encouragement to engage in future behaviours | Does not encourage me at all | Not encouraged |
|  | Does not encourage me much |  |
|  | Somewhat encourages me | Encouraged |
|  | Greatly encourages me |  |

# Supplementary Table 2: Current behaviours – by familiarity

| Dependent variable | Predictor and covariates | Odds Ratio (95% CI) | P-value |
| --- | --- | --- | --- |
| Attending places of worship | Familiarity with cognitive frailty       Age (ref. Young adults)            Middle-aged            Young-old            Old-old       Gender (ref. Male)            Female            Other       Ethnicity (ref. White)            Non-White            Prefer not to say      Deprivation (ref. Quintile 1 - Most deprived)            Quintile 2            Quintile 3            Quintile 4            Quintile 5 – Least deprived       Education (ref. Lower)            Middle            Higher            Other      Physical health (ref. Poor/fair)            Good            Very good/excellent | 0.864 (0.714, 1.047)  1.231 (0.790, 1.917)  1.980 (1.298, 3.021)  3.393 (2.057, 5.597)    0.775 (0.655, 0.918)  0.000 (0.000, -)    2.685 (1.816, 3.972)  2.693 (0.751, 9.653)    0.867 (0.598, 1.259)  0.964 (0.676, 1.376)  0.983 (0.698, 1.385)  0.997 (0.710, 1.398)    0.800 (0.609, 1.049)  1.130 (0.899, 1.422)  1.798 (1.269, 2.548)    1.056 (0.850, 1.311)  0.979 (0.794, 1.207) | p>0.05  **p<0.001***  p>0.05  **p<0.01**  **p<0.001***  **p<0.05**  **p<0.01**  p>0.05  **p<0.001***  **p<0.001***  p>0.05  p>0.05  p>0.05  p>0.05  p>0.05  p>0.05  **p<0.001***  p>0.05  p>0.05  **p<0.001***  p>0.05  p>0.05  p>0.05 |
| Balance exercises | Familiarity with cognitive frailty       Age (ref. Young adults)            Middle-aged            Young-old            Old-old       Gender (ref. Male)            Female            Other       Ethnicity (ref. White)            Non-White            Prefer not to say       Deprivation (ref. Quintile 1 - Most deprived)            Quintile 2            Quintile 3            Quintile 4            Quintile 5 – Least deprived       Education (ref. Lower)            Middle            Higher            Other      Physical health (ref. Poor/fair)            Good            Very good/excellent | 0.757 (0.644, 0.889)  1.527 (1.096, 2.126)  2.585 (1.880, 3.555)  2.564 (1.691, 3.887)    0.361 (0.312, 0.417)  2.014 (0.462, 8.775)    1.284 (0.863, 1.910)  1.608 (0.428, 6.041)    1.273 (0.929, 1.745)  1.486 (1.095, 2.017)  1.410 (1.051, 1.891)  1.573 (1.176, 2.104)    1.327 (1.059, 1.664)  2.047 (1.676, 2.499)  1.379 (0.992, 1.916)    1.548 (1.285, 1.864)  2.243 (1.874, 2.686) | **p<0.001***  **p<0.001***  **p<0.05**  **p<0.001***  **p<0.001***  **p<0.001***  **p<0.001***  p>0.05  p>0.05  p>0.05  p>0.05  **p<0.05**  p>0.05  **p<0.05**  **p<0.05**  **p<0.01**  **p<0.001***  **p<0.05**  **p<0.001***  p>0.05  **p<0.001***  **p<0.001***  **p<0.001*** |
| Book club | Familiarity with cognitive frailty       Age (ref. Young adults)            Middle-aged            Young-old            Old-old       Gender (ref. Male)            Female            Other       Ethnicity (ref. White)            Non-White            Prefer not to say       Deprivation (ref. Quintile 1 - Most deprived)            Quintile 2            Quintile 3            Quintile 4            Quintile 5 – Least deprived       Education (ref. Lower)            Middle            Higher            Other      Physical health (ref. Poor/fair)            Good            Very good/excellent | 0.839 (0.679, 1.037)  1.196 (0.740, 1.933)  2.178 (1.382, 3.431)  2.946 (1.698, 5.113)    0.434 (0.354, 0.532)  0.467 (0.054, 4.042)    2.072 (1.336, 3.215)  7.034 (1.955, 25.309)    1.213 (0.766, 1.920)  1.549 (1.000, 2.397)  1.384 (0.903, 2.123)  1.694 (1.113, 2.579)    0.989 (0.721, 1.355)  1.642 (1.259, 2.142)  1.303 (0.854, 1.989)    1.361 (1.053, 1.758)  1.555 (1.218, 1.985) | p>0.05  **p<0.001***  p>0.05  **p<0.001***  **p<0.001***  **p<0.001***  **p<0.001***  p>0.05  **p<0.001***  **p<0.01***  **p<0.01**  **p<0.05**  p>0.05  p>0.05  p>0.05  **p<0.05**  **p<0.001***  p>0.05  **p<0.001***  p>0.05  **p<0.01**  **p<0.05**  **p<0.001*** |
| Cardiovascular exercises | Familiarity with cognitive frailty       Age (ref. Young adults)            Middle-aged            Young-old            Old-old       Gender (ref. Male)            Female            Other       Ethnicity (ref. White)            Non-White            Prefer not to say       Deprivation (ref. Quintile 1 - Most deprived)            Quintile 2            Quintile 3            Quintile 4            Quintile 5 – Least deprived       Education (ref. Lower)            Middle            Higher            Other      Physical health (ref. Poor/fair)            Good            Very good/excellent | 0.743 (0.629, 0.876)  0.830 (0.575, 1.199)  0.668 (0.470, 0.950)  0.341 (0.220, 0.530)  1.767 (1.513, 2.065)  1.471 (0.354, 6.107)  0.959 (0.638, 1.440)  0.420 (0.112, 1.569)  1.044 (0.757, 1.441)  1.346 (0.983, 1.842)  1.221 (0.903, 1.650)  1.337 (0.992, 1.803)  1.106 (0.882, 1.388)  1.936 (1.584, 2.366)  1.420 (1.016, 1.983)  1.914 (1.592, 2.302)  3.776 (3.145, 4.533) | **p<0.001***  **p<0.001***  p>0.05  **p<0.05**  **p<0.001***  **p<0.001***  **p<0.001***  p>0.05  p>0.05  p>0.05  p>0.05  p>0.05  p>0.05  p<0.05  p>0.05  p>0.05  **p<0.001***  p>0.05  **p<0.001***  **p<0.05**  **p<0.001***  **p<0.001***  **p<0.001*** |
| Challenging brain | Familiarity with cognitive frailty       Age (ref. Young adults)            Middle-aged            Young-old            Old-old       Gender (ref. Male)            Female            Other       Ethnicity (ref. White)            Non-White            Prefer not to say       Deprivation (ref. Quintile 1 - Most deprived)            Quintile 2            Quintile 3            Quintile 4            Quintile 5 – Least deprived       Education (ref. Lower)            Middle            Higher            Other      Physical health (ref. Poor/fair)            Good            Very good/excellent | 0.592 (0.413, 0.848)  1.415 (0.758, 2.640)  2.357 (1.282, 4.335)  3.674 (1.269, 10.634)  0.922 (0.643, 1.322)  107325505.28 (0.000, -)  0.303 (0.168, 0.549)  66858634.991 (0.000, -)  1.086 (0.564, 2.093)  1.334 (0.688, 2.583)  1.535 (0.809, 2.914)  1.375 (0.736, 2.570)  1.426 (0.856, 2.376)  1.780 (1.140, 2.779)  1.973 (0.802, 4.854)  1.189 (0.760, 1.860)  1.208 (0.785, 1.857) | **p<0.01**  **p<0.01**  p>0.05  **p<0.01**  **p<0.05**  p>0.05  p>0.05  p>0.05  **p<0.001***  **p<0.001***  p>0.05  p>0.05  p>0.05  p>0.05  p>0.05  p>0.05  p>0.05  p>0.05  **p<0.05**  p>0.05  p>0.05  p>0.05  p>0.05 |
| Community based activities | Familiarity with cognitive frailty       Age (ref. Young adults)            Middle-aged            Young-old            Old-old       Gender (ref. Male)            Female            Other       Ethnicity (ref. White)            Non-White            Prefer not to say       Deprivation (ref. Quintile 1 - Most deprived)            Quintile 2            Quintile 3            Quintile 4            Quintile 5 – Least deprived       Education (ref. Lower)            Middle            Higher            Other      Physical health (ref. Poor/fair)            Good            Very good/excellent | 0.737 (0.629, 0.864)  1.642 (1.161, 2.322)  3.620 (2.594, 5.052)  5.041 (3.293, 7.714)  0.569 (0.493, 0.655)  1.390 (0.358, 5.397)  0.952 (0.645, 1.403)  1.299 (0.368, 4.583)  0.862 (0.630, 1.180)  1.183 (0.875, 1.599)  1.243 (0.929, 1.663)  1.035 (0.776, 1.380)  1.328 (1.061, 1.662)  1.902 (1.561, 2.317)  1.402 (1.015, 1.937)  1.358 (1.129, 1.634)  1.721 (1.441, 2.056) | **p<0.001***  **p<0.001***  **p<0.01**  **p<0.001***  **p<0.001***  **p<0.001***  **p<0.001***  p>0.05  p>0.05  p>0.05  p>0.05  **p<0.01**  p>0.05  p>0.05  p>0.05  p>0.05  **p<0.001***  **p<0.05**  **p<0.001***  **p<0.05**  **p<0.001***  **p<0.01***  **p<0.001*** |
| Drinking 2 litres of water | Familiarity with cognitive frailty       Age (ref. Young adults)            Middle-aged            Young-old            Old-old       Gender (ref. Male)            Female            Other       Ethnicity (ref. White)            Non-White            Prefer not to say       Deprivation (ref. Quintile 1 - Most deprived)            Quintile 2            Quintile 3            Quintile 4            Quintile 5 – Least deprived       Education (ref. Lower)            Middle            Higher            Other      Physical health (ref. Poor/fair)            Good            Very good/excellent | 0.652 (0.496, 0.857)  0.726 (0.349, 1.511)  0.675 (0.333, 1.367)  0.558 (0.244, 1.277)  0.778 (0.602, 1.007)  0.616 (0.074, 5.098)  1.457 (0.629, 3.375)  0.745 (0.093, 5.963)  0.798 (0.461, 1.381)  1.247 (0.716, 2.173)  1.299 (0.760, 2.220)  1.124 (0.666, 1.897)  0.945 (0.636, 1.404)  1.283 (0.897, 1.835)  1.027 (0.571, 1.849)  1.008 (0.721, 1.408)  1.158 (0.834, 1.608) | **p<0.01**  p>0.05  p>0.05  p>0.05  p>0.05  p>0.05  p>0.05  p>0.05  p>0.05  p>0.05  p>0.05  p>0.05  p>0.05  p>0.05  p>0.05  p>0.05  p>0.05  p>0.05  p>0.05  p>0.05  p>0.05  p>0.05  p>0.05 |
| Drinking alcohol | Familiarity with cognitive frailty       Age (ref. Young adults)            Middle-aged            Young-old            Old-old       Gender (ref. Male)            Female            Other       Ethnicity (ref. White)            Non-White            Prefer not to say       Deprivation (ref. Quintile 1 - Most deprived)            Quintile 2            Quintile 3            Quintile 4            Quintile 5 – Least deprived       Education (ref. Lower)            Middle            Higher            Other      Physical health (ref. Poor/fair)            Good            Very good/excellent | 0.838 (0.696, 1.009)  1.264 (0.887, 1.803)  1.557 (1.109, 2.186)  1.604 (1.004, 2.562)  1.722 (1.434, 2.066)  1.873 (0.380, 9.244)  0.399 (0.271, 0.588)  0.391 (0.106, 1.439)  1.125 (0.800, 1.583)  1.540 (1.097, 2.162)  1.306 (0.947, 1.802)  1.440 (1.047, 1.981)  1.031 (0.792, 1.342)  1.097 (0.871, 1.383)  0.914 (0.628, 1.330)  1.759 (1.433, 2.160)  2.066 (1.694, 2.518) | p>0.05  **p<0.05**  p>0.05  **p<0.05**  **p<0.05**  **p<0.001***  **p<0.001***  p>0.05  **p<0.001***  **p<0.001***  p>0.05  **p<0.05**  p>0.05  **p<0.05**  p>0.05  **p<0.05**  p>0.05  p>0.05  p>0.05  p>0.05  **p<0.001***  **p<0.001***  **p<0.001*** |
| Getting enough sleep | Familiarity with cognitive frailty       Age (ref. Young adults)            Middle-aged            Young-old            Old-old       Gender (ref. Male)            Female            Other       Ethnicity (ref. White)            Non-White            Prefer not to say       Deprivation (ref. Quintile 1 - Most deprived)            Quintile 2            Quintile 3            Quintile 4            Quintile 5 – Least deprived       Education (ref. Lower)            Middle            Higher            Other      Physical health (ref. Poor/fair)            Good            Very good/excellent | 0.798 (0.550, 1.159)  1.525 (0.762, 3.053)  2.312 (1.181, 4.527)  2.289 (0.920, 5.695)  1.067 (0.737, 1.544)  0.440 (0.047, 4.120)  1.378 (0.491, 3.872)  98967430.553 (0.000, -)  0.630 (0.276, 1.440)  0.608 (0.268, 1.382)  0.593 (0.268, 1.312)  0.572 (0.260, 1.257)  1.719 (1.042, 2.836)  1.840 (1.201, 2.820)  1.523 (0.708, 3.275)  2.170 (1.522, 3.093)  8.167 (5.073, 13.150) | p>0.05  **p<0.05**  p>0.05  **p<0.05**  p>0.05  p>0.05  p>0.05  p>0.05  p>0.05  p>0.05  p>0.05  p>0.05  p>0.05  p>0.05  p>0.05  p>0.05  **p<0.05**  **p<0.05**  **p<0.01**  p>0.05  **p<0.001***  **p<0.001***  **p<0.001*** |
| Exposure to air pollution | Familiarity with cognitive frailty       Age (ref. Young adults)            Middle-aged            Young-old            Old-old       Gender (ref. Male)            Female            Other       Ethnicity (ref. White)            Non-White            Prefer not to say       Deprivation (ref. Quintile 1 - Most deprived)            Quintile 2            Quintile 3            Quintile 4            Quintile 5 – Least deprived       Education (ref. Lower)            Middle            Higher            Other      Physical health (ref. Poor/fair)            Good            Very good/excellent | 0.512 (0.424, 0.617)  1.382 (0.948, 2.015)  1.990 (1.385, 2.860)  1.912 (1.162, 3.147)  0.930 (0.773, 1.119)  2.863 (0.349, 23.508)  1.070 (0.659, 1.738)  0.364 (0.101, 1.319)  0.991 (0.683, 1.439)  1.490 (1.025, 2.165)  1.271 (0.893, 1.811)  1.273 (0.897, 1.805)  1.452 (1.098, 1.919)  1.458 (1.147, 1.851)  1.971 (1.240, 3.132)  0.990 (0.786, 1.246)  1.343 (1.070, 1.687) | **p<0.001***  **p<0.001***  p>0.05  **p<0.001***  **p<0.05**  p>0.05  p>0.05  p>0.05  p>0.05  p>0.05  p>0.05  **p<0.05**  p>0.05  **p<0.05**  p>0.05  p>0.05  **p<0.01**  **p<0.01**  **p<0.01**  **p<0.01**  **p<0.01**  p>0.05  **p<0.05** |
| Eating fruits and vegetables | Familiarity with cognitive frailty       Age (ref. Young adults)            Middle-aged            Young-old            Old-old       Gender (ref. Male)            Female            Other       Ethnicity (ref. White)            Non-White            Prefer not to say       Deprivation (ref. Quintile 1 - Most deprived)            Quintile 2            Quintile 3            Quintile 4            Quintile 5 – Least deprived       Education (ref. Lower)            Middle            Higher            Other      Physical health (ref. Poor/fair)            Good            Very good/excellent | 0.449 (0.105, 1.912)  0.000 (0.000, -)  0.000 (0.000, -)  0.000 (0.000, -)    0.470 (0.114, 1.944)  510235.794 (0.000, -)  2216861.734 (0.000, -)  2600167.776 (0.000, -)  6829491.797 (0.000, -)  2.019 (0.177, 23.083)  1.862 (0.189, 18.373)  3.328 (0.290, 38.159)  0.000 (0.000, -)  0.000 (0.000, -)  0.000 (0.000, -)  1.244 (0.206, 7.530)  1.701 (0.277, 10.425) | p>0.05  p>0.05  p>0.05  p>0.05  p>0.05  p>0.05  p>0.05  p>0.05  p>0.05  p>0.05  p>0.05  p>0.05  p>0.05  p>0.05  p>0.05  p>0.05  p>0.05  p>0.05  p>0.05  p>0.05  p>0.05  p>0.05  p>0.05 |
| Further education | Familiarity with cognitive frailty       Age (ref. Young adults)            Middle-aged            Young-old            Old-old       Gender (ref. Male)            Female            Other       Ethnicity (ref. White)            Non-White            Prefer not to say       Deprivation (ref. Quintile 1 - Most deprived)            Quintile 2            Quintile 3            Quintile 4            Quintile 5 – Least deprived       Education (ref. Lower)            Middle            Higher            Other      Physical health (ref. Poor/fair)            Good            Very good/excellent | 0.713 (0.610, 0.834)  0.678 (0.485, 0.948)  0.631 (0.457, 0.869)  0.759 (0.503, 1.144)  0.766 (0.667, 0.880)  1.078 (0.291, 3.998)  1.752 (1.182, 2.595)  1.252 (0.350, 4.483)  0.829 (0.610, 1.126)  0.863 (0.642, 1.160)  1.017 (0.764, 1.352)  0.858 (0.647, 1.138)  1.475 (1.178, 1.848)  2.450 (2.009, 2.988)  1.528 (1.107, 2.108)  1.168 (0.973, 1.401)  1.251 (1.051, 1.489) | **p<0.001***  **p<0.05**  **p<0.05**  **p<0.01**  p>0.05  **p<0.001***  **p<0.001***  p>0.05  **p<0.05**  **p<0.01**  p>0.05  p>0.05  p>0.05  p>0.05  p>0.05  p>0.05  **p<0.001***  **p<0.001***  **p<0.001***  **p<0.05**  **p<0.05**  p>0.05  **p<0.05** |
| Using green spaces | Familiarity with cognitive frailty       Age (ref. Young adults)            Middle-aged            Young-old            Old-old       Gender (ref. Male)            Female            Other       Ethnicity (ref. White)            Non-White            Prefer not to say       Deprivation (ref. Quintile 1 - Most deprived)            Quintile 2            Quintile 3            Quintile 4            Quintile 5 – Least deprived       Education (ref. Lower)            Middle            Higher            Other      Physical health (ref. Poor/fair)            Good            Very good/excellent | 0393 (0.258, 0.598)  1.649 (0.755, 3.601)  2.280 (1.068, 4.869)  2.207 (0.744, 6.545)  0.906 (0.579, 1.416)  0.151 (0.024, 0.946)  0.196 (0.102, 0.380)  46974570.636 (0.000, -)  1.238 (0.573, 2.678)  1.374 (0.625, 3.020)  1.435 (0.674, 3.053)  1.543 (0.723, 3.290)  0.958 (0.528, 1.739)  1.948 (1.103, 3.442)  1.845 (0.610, 5.575)  2.234 (1.405, 3.554)  4.906 (2.858, 8.419) | **p<0.001***  p>0.05  p>0.05  **p<0.05**  p>0.05  p>0.05  p>0.05  **p<0.05**  **p<0.001***  **p<0.001***  p>0.05  p>0.05  p>0.05  p>0.05  p>0.05  p>0.05  **p<0.05**  p>0.05  **p<0.05**  p>0.05  **p<0.001***  **p<0.001***  **p<0.001*** |
| Wearing hearing aids | Familiarity with cognitive frailty       Age (ref. Young adults)            Middle-aged            Young-old            Old-old       Gender (ref. Male)            Female            Other       Ethnicity (ref. White)            Non-White            Prefer not to say       Deprivation (ref. Quintile 1 - Most deprived)            Quintile 2            Quintile 3            Quintile 4            Quintile 5 – Least deprived       Education (ref. Lower)            Middle            Higher            Other      Physical health (ref. Poor/fair)            Good            Very good/excellent | 0.983 (0.805, 1.201)  2.245 (0.879, 5.736)  10.193 (4.123, 25.200)  34.844 (13.630, 89.072)  1.609 (1.361, 1.904)  2.276 (0.387, 13.369)  0.559 (0.298, 1.050)  2.779 (0.718, 10.750)  0.919 (0.613, 1.379)  0.822 (0.556, 1.214)  0.880 (0.604, 1.281)  0.863 (0.595, 1.251)  1.173 (0.890, 1.544)  1.016 (0.796, 1.297)  1.009 (0.678, 1.500)  0.839 (0.671, 1.050)  0.721 (0.580, 0.896) | p>0.05  **p<0.001***  p>0.05  **p<0.001***  **p<0.001***  **p<0.001***  **p<0.001***  p>0.05  p>0.05  p>0.05  p>0.05  p>0.05  p>0.05  p>0.05  p>0.05  p>0.05  p>0.05  p>0.05  p>0.05  p>0.05  **p<0.05**  p>0.05  **p<0.01** |
| Taking illegal substances | Familiarity with cognitive frailty       Age (ref. Young adults)            Middle-aged            Young-old            Old-old       Gender (ref. Male)            Female            Other       Ethnicity (ref. White)            Non-White            Prefer not to say       Deprivation (ref. Quintile 1 - Most deprived)            Quintile 2            Quintile 3            Quintile 4            Quintile 5 – Least deprived       Education (ref. Lower)            Middle            Higher            Other      Physical health (ref. Poor/fair)            Good            Very good/excellent | 1.236 (0.770, 1.984)  0.673 (0.327, 1.386)  0.328 (0.160, 0.673)  0.359 (0.117, 1.101)  1.758 (1.149, 2.688)  0.000 (0.000, -)  1.675 (0.735, 3.819)  0.000 (0.000, -)  0.657 (0.325, 1.328)  0.354 (0.163, 0.767)  0.372 (0.183, 0.756)  0.415 (0.209, 0.823)  1.351 (0.609, 2.997)  1.529 (0.746, 3.135)  1.223 (0.370, 4.048)  0.793 (0.465, 1.351)  0.626 (0.368, 1.063) | p>0.05  **p<0.01**  p>0.05  **p<0.01**  p>0.05  **p<0.05**  **p<0.01**  p>0.05  p>0.05  p>0.05  p>0.05  **p<0.05**  p>0.05  **p<0.01**  **p<0.01**  **p<0.05**  p>0.05  p>0.05  p>0.05  p>0.05  p>0.05  p>0.05  p>0.05 |
| Managing mental wellbeing | Familiarity with cognitive frailty       Age (ref. Young adults)            Middle-aged            Young-old            Old-old       Gender (ref. Male)            Female            Other       Ethnicity (ref. White)            Non-White            Prefer not to say       Deprivation (ref. Quintile 1 - Most deprived)            Quintile 2            Quintile 3            Quintile 4            Quintile 5 – Least deprived       Education (ref. Lower)            Middle            Higher            Other      Physical health (ref. Poor/fair)            Good            Very good/excellent | 0.536 (0.400, 0.718)  0.576 (0.222, 1.494)  0.525 (0.208, 1.327)  0.509 (0.178, 1.452)  0.570 (0.432, 0.753)  0.313 (0.037, 2.613)  1.294 (0.517, 3.243)  0.311 (0.063, 1.524)  0.593 (0.279, 1.262)  0.745 (0.353, 1.574)  0.614 (0.299, 1.261)  0.605 (0.296, 1.237)  1.183 (0.766, 1.829)  1.417 (0.966, 2.079)  1.570 (0.764, 3.225)  0.988 (0.685, 1.427)  1.314 (0.911, 1.897) | **p<0.001***  p>0.05  p>0.05  p>0.05  p>0.05  **p<0.001***  **p<0.001***  p>0.05  p>0.05  p>0.05  p>0.05  p>0.05  p>0.05  p>0.05  p>0.05  p>0.05  p>0.05  p>0.05  p>0.05  p>0.05  p>0.05  p>0.05  p>0.05 |
| Managing weight | Familiarity with cognitive frailty       Age (ref. Young adults)            Middle-aged            Young-old            Old-old       Gender (ref. Male)            Female            Other       Ethnicity (ref. White)            Non-White            Prefer not to say       Deprivation (ref. Quintile 1 - Most deprived)            Quintile 2            Quintile 3            Quintile 4            Quintile 5 – Least deprived       Education (ref. Lower)            Middle            Higher            Other      Physical health (ref. Poor/fair)            Good            Very good/excellent | 0.651 (0.506, 0.838)  1.490 (0.921, 2.412)  2.063 (1.299, 3.277)  1.493 (0.813, 2.741)  0.894 (0.700, 1.143)  1.185 (0.142, 9.878)  0.697 (0.400, 1.215)  181789247.49 (0.000, -)  0.708 (0.418, 1.196)  1.021 (0.598, 1.742)  0.866 (0.521, 1.439)  0.849 (0.514, 1.404)  1.229 (0.846, 1.787)  1.283 (0.927, 1.774)  1.149 (0.660, 1.999)  1.384 (1.045, 1.833)  2.252 (1.686, 3.010) | **p<0.001***  **p<0.01**  p>0.05  **p<0.01**  p>0.05  p>0.05  p>0.05  p>0.05  p>0.05  p>0.05  p>0.05  p>0.05  p>0.05  p>0.05  p>0.05  p>0.05  p>0.05  p>0.05  p>0.05  p>0.05  **p<0.001***  **p<0.05**  **p<0.001*** |
| Mindful Activities | Familiarity with cognitive frailty       Age (ref. Young adults)            Middle-aged            Young-old            Old-old       Gender (ref. Male)            Female            Other       Ethnicity (ref. White)            Non-White            Prefer not to say       Deprivation (ref. Quintile 1 - Most deprived)            Quintile 2            Quintile 3            Quintile 4            Quintile 5 – Least deprived       Education (ref. Lower)            Middle            Higher            Other      Physical health (ref. Poor/fair)            Good            Very good/excellent | 0.705 (0.602, 0.824)  1.104 (0.798, 1.528)  1.298 (0.951, 1.770)  1.487 (0.992, 2.228)  0.460 (0.400, 0.529)  1.990 (0.498, 7.944)  2.168 (1.454, 3.233)  1.174 (0.329, 4.197)  0.794 (0.585, 1.077)  0.952 (0.709, 1.279)  1.001 (0.753, 1.331)  0.925 (0.698, 1.225)  1.197 (0.960, 1.493)  1.622 (1.336, 1.968)  1.504 (1.092, 2.073)  0.941 (0.784, 1.128)  0.870 (0.731, 1.036) | **p<0.001***  p>0.05  p>0.05  p>0.05  p>0.05  **p<0.001***  **p<0.001***  p>0.05  **p<0.001***  **p<0.001***  p>0.05  p>0.05  p>0.05  p>0.05  p>0.05  p>0.05  **p<0.001***  p>0.05  **p<0.001***  **p<0.05**  p>0.05  p>0.05  p>0.05 |
| Being near water | Familiarity with cognitive frailty       Age (ref. Young adults)            Middle-aged            Young-old            Old-old       Gender (ref. Male)            Female            Other       Ethnicity (ref. White)            Non-White            Prefer not to say       Deprivation (ref. Quintile 1 - Most deprived)            Quintile 2            Quintile 3            Quintile 4            Quintile 5 – Least deprived       Education (ref. Lower)            Middle            Higher            Other      Physical health (ref. Poor/fair)            Good            Very good/excellent | 0.584 (0.482, 0.707)  1.113 (0.753, 1.646)  1.624 (1.113, 2.368)  1.104 (0.679, 1.795)  1.030 (0.853, 1.244)  0.661 (0.161, 2.720)  0.478 (0.316, 0.723)  2.188 (0.273, 17.539)  1.307 (0.903, 1.892)  1.619 (1.122, 2.337)  1.649 (1.161, 2.343)  1.267 (0.901, 1.781)  1.304 (0.980, 1.734)  1.293 (1.011, 1.654)  1.420 (0.916, 2.201)  1.593 (1.280, 1.981)  2.344 (1.885, 2.915) | **p<0.001***  **p<0.001***  p>0.05  **p<0.05**  p>0.05  p>0.05  p>0.05  p>0.05  **p<0.01**  **p<0.001***  p>0.05  **p<0.05**  p>0.05  **p<0.05**  **p<0.01**  p>0.05  p>0.05  p>0.05  **p<0.05**  p>0.05  **p<0.001***  **p<0.001***  **p<0.001*** |
| Eating nuts, seeds and legumes | Familiarity with cognitive frailty       Age (ref. Young adults)            Middle-aged            Young-old            Old-old       Gender (ref. Male)            Female            Other       Ethnicity (ref. White)            Non-White            Prefer not to say       Deprivation (ref. Quintile 1 - Most deprived)            Quintile 2            Quintile 3            Quintile 4            Quintile 5 – Least deprived       Education (ref. Lower)            Middle            Higher            Other      Physical health (ref. Poor/fair)            Good            Very good/excellent | 0.601 (0.419, 0.862)  2.985 (1.660, 5.366)  4.787 (2.729, 8.398)  14.924 (4.213, 52.869)  0.700 (0.492, 0.997)  215355034.38 (0.000, -)  1.602 (0.563, 4.555)  82126474.244 (0.000, -)  1.103 (0.602, 2.021)  1.758 (0.924, 3.346)  1.925 (1.043, 3.551)  1.448 (0.807, 2.600)  1.244 (0.801, 1.932)  3.368 (2.174, 5.216)  2.489 (1.025, 6.046)  1.791 (1.216, 2.639)  3.335 (2.193, 5.070) | **p<0.01**  **p<0.001***  **p<0.001***  **p<0.001***  **p<0.001***  p>0.05  **p<0.05**  p>0.05  p>0.05  p>0.05  p>0.05  p>0.05  p>0.05  p>0.05  **p<0.05**  p>0.05  **p<0.001***  p>0.05  **p<0.001***  **p<0.05**  **p<0.001***  **p<0.01**  **p<0.001*** |
| Eating processed foods | Familiarity with cognitive frailty       Age (ref. Young adults)            Middle-aged            Young-old            Old-old       Gender (ref. Male)            Female            Other       Ethnicity (ref. White)            Non-White            Prefer not to say       Deprivation (ref. Quintile 1 - Most deprived)            Quintile 2            Quintile 3            Quintile 4            Quintile 5 – Least deprived       Education (ref. Lower)            Middle            Higher            Other      Physical health (ref. Poor/fair)            Good            Very good/excellent | 1.055 (0.783, 1.421)  0.147 (0.035, 0.610)  0.100 (0.024, 0.410)  0.065 (0.015, 0.280)  1.882 (1.411, 2.511)  101196832.26 (0.000, -)  0.379 (0.216, 0.665)  0.601 (0.075, 4.839)  0.956 (0.532, 1.719)  0.936 (0.533, 1.645)  0.993 (0.574, 1.718)  1.030 (0.598, 1.773)  1.017 (0.688, 1.502)  1.196 (0.849, 1.685)  0.835 (0.495, 1.409)  1.067 (0.762, 1.494)  0.992 (0.720, 1.366) | p>0.05  **p<0.001***  **p<0.01**  **p<0.01**  **p<0.001***  **p<0.001***  **p<0.001***  p>0.05  **p<0.01**  **p<0.001***  p>0.05  p>0.05  p>0.05  p>0.05  p>0.05  p>0.05  p>0.05  p>0.05  p>0.05  p>0.05  p>0.05  p>0.05  p>0.05 |
| Protecting against hearing loss | Familiarity with cognitive frailty       Age (ref. Young adults)            Middle-aged            Young-old            Old-old       Gender (ref. Male)            Female            Other       Ethnicity (ref. White)            Non-White            Prefer not to say       Deprivation (ref. Quintile 1 - Most deprived)            Quintile 2            Quintile 3            Quintile 4            Quintile 5 – Least deprived       Education (ref. Lower)            Middle            Higher            Other      Physical health (ref. Poor/fair)            Good            Very good/excellent | 0.732 (0.620, 0.865)  0.972 (0.686, 1.376)  1.238 (0.889, 1.724)  1.283 (0.842, 1.957)  1.389 (1.206, 1.599)  1.627 (0.450, 5.877)  0.995 (0.671, 1.474)  1.921 (0.545, 6.769)  0.934 (0.678, 1.288)  1.148 (0.844, 1.562)  1.213 (0.902, 1.632)  1.020 (0.760, 1.369)  1.086 (0.865, 1.364)  1.013 (0.829, 1.237)  0.983 (0.705, 1.370)  1.075 (0.888, 1.300)  1.176 (0.980, 1.410) | **p<0.001***  **p<0.05**  p>0.05  p>0.05  p>0.05  **p<0.001***  **p<0.001***  p>0.05  p>0.05  p>0.05  p>0.05  p>0.05  p>0.05  p>0.05  p>0.05  p>0.05  p>0.05  p>0.05  p>0.05  p>0.05  p>0.05  p>0.05  p>0.05 |
| Purpose in life | Familiarity with cognitive frailty       Age (ref. Young adults)            Middle-aged            Young-old            Old-old       Gender (ref. Male)            Female            Other       Ethnicity (ref. White)            Non-White            Prefer not to say       Deprivation (ref. Quintile 1 - Most deprived)            Quintile 2            Quintile 3            Quintile 4            Quintile 5 – Least deprived       Education (ref. Lower)            Middle            Higher            Other      Physical health (ref. Poor/fair)            Good            Very good/excellent | 0.466 (0.325, 0.667)  1.157 (0.520, 2.577)  1.427 (0.658, 3.096)  1.948 (0.668, 5.682)  0.610 (0.427, 0.871)  78585362.949 (0.000, -)  0.985 (0.385, 2.518)  0.479 (0.059, 3.924)  1.131 (0.565, 2.265)  1.869 (0.903, 3.869)  1.521 (0.776, 2.978)  1.406 (0.727, 2.716)  1.053 (0.602, 1.843)  1.114 (0.679, 1.830)  2.836 (0.832, 9.667)  2.245 (1.505, 3.347)  4.271 (2.758, 6.615) | **p<0.001***  p>0.05  p>0.05  p>0.05  p>0.05  **p<0.05**  **p<0.01**  p>0.05  p>0.05  p>0.05  p>0.05  p>0.05  p>0.05  p>0.05  p>0.05  p>0.05  p>0.05  p>0.05  p>0.05  p>0.05  **p<0.001***  **p<0.001***  **p<0.001*** |
| Eating red meat | Familiarity with cognitive frailty       Age (ref. Young adults)            Middle-aged            Young-old            Old-old       Gender (ref. Male)            Female            Other       Ethnicity (ref. White)            Non-White            Prefer not to say       Deprivation (ref. Quintile 1 - Most deprived)            Quintile 2            Quintile 3            Quintile 4            Quintile 5 – Least deprived       Education (ref. Lower)            Middle            Higher            Other      Physical health (ref. Poor/fair)            Good            Very good/excellent | 1.115 (0.907, 1.371)  0.796 (0.534, 1.188)  0.888 (0.605, 1.304)  1.054 (0.617, 1.802)  1.793 (1.474, 2.180)  1.167 (0.243, 5.610)  0.733 (0.471, 1.142)  0.712 (0.146, 3.461)  0.989 (0.682, 1.435)  1.065 (0.742, 1.528)  1.517 (1.063, 2.165)  1.406 (0.992, 1.993)  0.691 (0.502, 0.952)  0.519 (0.391, 0.689)  0.906 (0.564, 1.453)  0.925 (0.724, 1.181)  0.793 (0.629, 1.000) | p>0.05  p>0.05  p>0.05  p>0.05  p>0.05  **p<0.001***  **p<0.001***  p>0.05  p>0.05  p>0.05  p>0.05  **p<0.01***  p>0.05  p>0.05  **p<0.05**  p>0.05  **p<0.001***  **p<0.05**  **p<0.001***  p>0.05  p>0.05  p>0.05  p>0.05 |
| Smoking or vaping | Familiarity with cognitive frailty       Age (ref. Young adults)            Middle-aged            Young-old            Old-old       Gender (ref. Male)            Female            Other       Ethnicity (ref. White)            Non-White            Prefer not to say       Deprivation (ref. Quintile 1 - Most deprived)            Quintile 2            Quintile 3            Quintile 4            Quintile 5 – Least deprived       Education (ref. Lower)            Middle            Higher            Other      Physical health (ref. Poor/fair)            Good            Very good/excellent | 0.814 (0.567, 1.170)  0.407 (0.260, 0.639)  0.131 (0.083, 0.208)  0.111 (0.046, 0.264)  1.195 (0.870, 1.641)  0.000 (0.000, -)  1.036 (0.524, 2.050)  1.950 (0.241, 15.814)  0.591 (0.363, 0.961)  0.418 (0.251, 0.696)  0.366 (0.227, 0.592)  0.364 (0.227, 0.586)  0.680 (0.441, 1.050)  0.479 (0.325, 0.706)  0.636 (0.309, 1.309)  0.551 (0.386, 0.786)  0.425 (0.299, 0.606) | p>0.05  **p<0.001***  **p<0.001***  **p<0.001***  **p<0.001***  p>0.05  p>0.05  p>0.05  p>0.05  p>0.05  p>0.05  **p<0.001***  **p<0.05**  **p<0.001***  **p<0.001***  **p<0.001***  **p<0.01**  p>0.05  **p<0.001***  p>0.05  **p<0.001***  **p<0.001***  **p<0.001*** |
| Socialising | Familiarity with cognitive frailty       Age (ref. Young adults)            Middle-aged            Young-old            Old-old       Gender (ref. Male)            Female            Other       Ethnicity (ref. White)            Non-White            Prefer not to say       Deprivation (ref. Quintile 1 - Most deprived)            Quintile 2            Quintile 3            Quintile 4            Quintile 5 – Least deprived       Education (ref. Lower)            Middle            Higher            Other      Physical health (ref. Poor/fair)            Good            Very good/excellent | 0.615 (0.428, 0.883)  0.367 (0.106, 1.270)  0.338 (0.100, 1.145)  0.433 (0.109, 1.719)  0.479 (0.341, 0.671)  0.200 (0.023, 1.732)  0.478 (0.231, 0.987)  93601263.112 (0.000, -)  1.258 (0.641, 2.468)  1.728 (0.875, 3.414)  1.871 (0.970, 3.606)  1.641 (0.869, 3.102)  1.124 (0.651, 1.942)  1.099 (0.680, 1.777)  3.146 (0.929, 10.654)  1.667 (1.122, 2.475)  3.123 (2.038, 4.786) | **p<0.01**  p>0.05  p>0.05  p>0.05  p>0.05  **p<0.001***  **p<0.001***  p>0.05  p>0.05  **p<0.05**  p>0.05  p>0.05  p>0.05  p>0.05  p>0.05  p>0.05  p>0.05  p>0.05  p>0.05  p>0.05  **p<0.001***  **p<0.05**  **p<0.001*** |
| Strength training | Familiarity with cognitive frailty       Age (ref. Young adults)            Middle-aged            Young-old            Old-old       Gender (ref. Male)            Female            Other       Ethnicity (ref. White)            Non-White            Prefer not to say       Deprivation (ref. Quintile 1 - Most deprived)            Quintile 2            Quintile 3            Quintile 4            Quintile 5 – Least deprived       Education (ref. Lower)            Middle            Higher            Other      Physical health (ref. Poor/fair)            Good            Very good/excellent | 0.784 (0.670, 0.918)  0.980 (0.706, 1.360)  0.935 (0.683, 1.279)  0.480 (0.317, 0.728)  0.975 (0.832, 1.101)  4.632 (0.939, 22.844)  1.382 (0.943, 2.024)  1.328 (0.376, 4.690)  1.304 (0.956, 1.778)  1.357 (1.005, 1.831)  1.466 (1.097, 1.958)  1.659 (1.246, 2.209)  1.350 (1.079, 1.688)  1.607 (1.321, 1.956)  1.510 (1.092, 2.088)  1.512 (1.257, 1.820)  2.611 (2.186, 3.119) | **p<0.01**  **p<0.001***  p>0.05  p>0.05  **p<0.001***  p>0.05  p>0.05  p>0.05  p>0.05  p>0.05  p>0.05  **p<0.01**  p>0.05  **p<0.05**  **p<0.05**  **p<0.001***  **p<0.001***  **p<0.01**  **p<0.001***  **p<0.05**  **p<0.001***  **p<0.001***  **p<0.001*** |
| Taking vitamins and supplements | Familiarity with cognitive frailty       Age (ref. Young adults)            Middle-aged            Young-old            Old-old       Gender (ref. Male)            Female            Other       Ethnicity (ref. White)            Non-White            Prefer not to say       Deprivation (ref. Quintile 1 - Most deprived)            Quintile 2            Quintile 3            Quintile 4            Quintile 5 – Least deprived       Education (ref. Lower)            Middle            Higher            Other      Physical health (ref. Poor/fair)            Good            Very good/excellent | 0.794 (0.668, 0.943)  1.074 (0.738, 1.561)  1.138 (0.796, 1.628)  1.122 (0.708, 1.779)  0.642 (0.550, 0.749)  1.123 (0.234, 5.399)  1.256 (0.796, 1.980)  1.401 (0.292, 6.712)  0.663(0.457, 0.962)  0.632 (0.441, 0.906)  0.721 (0.508, 1.025)  0.735 (0.519, 1.041)  1.289 (1.007, 1.649)  1.314 (1.061, 1.628)  1.457 (1.001, 2.122)  1.062 (0.860, 1.313)  0.863 (0.706, 1.054) | **p<0.01**  p>0.05  p>0.05  p>0.05  p>0.05  **p<0.001***  **p<0.001***  p>0.05  p>0.05  p>0.05  p>0.05  p>0.05  **p<0.05**  **p<0.05**  p>0.05  p>0.05  p>0.05  **p<0.05**  **p<0.05**  p>0.05  **p<0.05**  p>0.05  p>0.05 |
| Volunteering and helping others | Familiarity with cognitive frailty       Age (ref. Young adults)            Middle-aged            Young-old            Old-old       Gender (ref. Male)            Female            Other       Ethnicity (ref. White)            Non-White            Prefer not to say       Deprivation (ref. Quintile 1 - Most deprived)            Quintile 2            Quintile 3            Quintile 4            Quintile 5 – Least deprived       Education (ref. Lower)            Middle            Higher            Other      Physical health (ref. Poor/fair)            Good            Very good/excellent | 0.649 (0.548, 0.768)  1.367 (0.976, 1.915)  2.376 (1.715, 3.289)  2.896 (1.851, 4.531)  0.562 (0.482, 0.656)  0.435 (0.115, 1.644)  1.076 (0.708, 1.635)  1.055 (0.268, 4.157)  1.043 (0.752, 1.446)  1.360 (0.986, 1.876)  1.262 (0.928, 1.716)  1.362 (1.004, 1.847)  0.989 (0.777, 1.259)  1.407 (1.134, 1.746)  1.469 (1.005, 2.148)  1.540 (1.265, 1.874)  1.816 (1.502, 2.196) | **p<0.001***  **p<0.001***  p>0.05  **p<0.001***  **p<0.001***  **p<0.001***  **p<0.001***  p>0.05  p>0.05  p>0.05  p>0.05  p>0.05  p>0.05  p>0.05  p>0.05  **p<0.05**  **p<0.001***  p>0.05  **p<0.01**  **p<0.05**  **p<0.001***  **p<0.001***  **p<0.001*** |

*Note.* Bolded comparisons are significant at p<0.05. * denotes comparisons remaining significant after Bonferroni correction at p<0.002.

# Supplementary Table 3: Current behaviours – by preventability

| Dependent variable | Predictor and covariates | Odds Ratio (95% CI) | P-value |
| --- | --- | --- | --- |
| Attending places of worship | Preventability of cognitive frailty       Age (ref. Young adults)            Middle-aged            Young-old            Old-old       Gender (ref. Male)            Female            Other       Ethnicity (ref. White)            Non-White            Prefer not to say      Deprivation (ref. Quintile 1 - Most deprived)            Quintile 2            Quintile 3            Quintile 4            Quintile 5 – Least deprived       Education (ref. Lower)            Middle            Higher            Other      Physical health (ref. Poor/fair)            Good            Very good/excellent | 0.894 (0.692, 1.154)  1.232 (0.791, 1.918)  1.990 (1.304, 3.037)  3.382 (2.050, 5.579)    0.765 (0.646, 0.906)  0.000 (0.000, -)    2.706 (1.829, 4.003)  2.704 (0.754, 9.702)    0.865 (0.596, 1.255)  0.956 (0.670, 1.363)  0.982 (0.697, 1.383)  0.995 (0.709, 1.395)    0.810 (0.616, 1.063)  1.144 (0.908, 1.442)  1.807 (1.275, 2.561)    1.068 (0.859, 1.327)  0.998 (0.809, 1.232) | p>0.05  **p<0.001***  p>0.05  **p<0.01***  **p<0.001***  **p<0.01**  **p<0.01**  p>0.05  **p<0.001***  **p<0.001***  p>0.05  p>0.05  p>0.05  p>0.05  p>0.05  p>0.05  **p<0.001***  p>0.05  p>0.05  **p<0.001***  p>0.05  p>0.05  p>0.05 |
| Balance exercises | Preventability of cognitive frailty       Age (ref. Young adults)            Middle-aged            Young-old            Old-old       Gender (ref. Male)            Female            Other       Ethnicity (ref. White)            Non-White            Prefer not to say       Deprivation (ref. Quintile 1 - Most deprived)            Quintile 2            Quintile 3            Quintile 4            Quintile 5 – Least deprived       Education (ref. Lower)            Middle            Higher            Other      Physical health (ref. Poor/fair)            Good            Very good/excellent | 1.726 (1.378, 2.163)  1.528 (1.097, 2.127)  2.587 (1.881, 3.558)  2.605 (1.716, 3.954)    0.359 (0.310, 0.414)  1.971 (0.460, 8.444)    1.257 (0.846, 1.869)  1.473 (0.392, 5.532)    1.262 (0.921, 1.730)  1.461 (1.076, 1.984)  1.407 (1.049, 1.889)  1.569 (1.173, 2.099)    1.279 (1.019, 1.606)  1.934 (1.581, 2.365)  1.364 (0.980, 1.899)    1.515 (1.257, 1.827)  2.162 (1.804, 2.593) | **p<0.001***  **p<0.001***  **p<0.05**  **p<0.001***  **p<0.001***  **p<0.001***  **p<0.001***  p>0.05  p>0.05  p>0.05  p>0.05  **p<0.05**  p>0.05  **p<0.05**  **p<0.05**  **p<0.01**  **p<.001***  **p<0.05**  **p>0.001***  p>0.05  **p<0.001***  **p<0.001***  **p<0.001*** |
| Book club | Preventability of cognitive frailty       Age (ref. Young adults)            Middle-aged            Young-old            Old-old       Gender (ref. Male)            Female            Other       Ethnicity (ref. White)            Non-White            Prefer not to say       Deprivation (ref. Quintile 1 - Most deprived)            Quintile 2            Quintile 3            Quintile 4            Quintile 5 – Least deprived       Education (ref. Lower)            Middle            Higher            Other      Physical health (ref. Poor/fair)            Good            Very good/excellent | 0.933 (0.695, 1.252)  1.193 (0.738, 1.928)  2.183 (1.385, 3.439)  2.931 (1.690, 5.085)    0.428 (0.350, 0.524)  2.931 (0.053, 3.929)    2.083 (1.343, 3.233)  7.004 (1.940, 25.286)    1.206 (0.762, 1.909)  1.531 (0.989, 2.369)  1.381 (0.901, 2.117)  1.687 (1.108, 2.568)    0.996 (0.726, 1.366)  1.651 (1.264, 2.158)  1.306 (0.856, 1.993)    1.372 (1.062, 1.774)  1.581 (1.237, 2.020) | p>0.05  **p<0.001***  p>0.05  **p<0.001***  **p<0.001***  **p<0.001***  **p<0.001***  p>0.05  **p<0.001***  **p<0.01***  **p<0.01**  **p<0.05**  p>0.05  p>0.05  p>0.05  **p<0.05**  **p<0.001***  p>0.05  **p<0.001***  p>0.05  **p<0.01***  **p<0.05**  **p<0.001*** |
| Cardiovascular exercises | Preventability of cognitive frailty       Age (ref. Young adults)            Middle-aged            Young-old            Old-old       Gender (ref. Male)            Female            Other       Ethnicity (ref. White)            Non-White            Prefer not to say       Deprivation (ref. Quintile 1 - Most deprived)            Quintile 2            Quintile 3            Quintile 4            Quintile 5 – Least deprived       Education (ref. Lower)            Middle            Higher            Other      Physical health (ref. Poor/fair)            Good            Very good/excellent | 2.014 (1.606, 2.527)  0.830 (0.574, 1.199)  0.665 (0.468, 0.947)  0.343 (0.221, 0.534)  1.780 (1.523, 2.081)  1.411 (0.348, 5.723)  0.924 (0.616, 1.385)  0.375 (0.101, 1.389)  1.032 (0.747, 1.425)  1.323 (0.966, 1.811)  1.220 (0.902, 1.650)  1.331 (0.987, 1.796)  1.051 (0.836, 1.321)  1.797 (1.467, 2.201)  1.394 (0.996, 1.952)  1.862 (1.547, 2.241)  3.603 (2.998, 4.330) | **p<0.001***  **p<0.001***  p>0.05  **p<0.05**  **p<0.001***  **p<0.001***  **p<0.001***  p>0.05  p>0.05  p>0.05  p>0.05  p>0.05  p>0.05  p>0.05  p>0.05  p>0.05  **p<0.001***  p>0.05  **p<0.001***  p>0.05  **p<0.001***  **p<0.001***  **p<0.001*** |
| Challenging brain | Preventability of cognitive frailty       Age (ref. Young adults)            Middle-aged            Young-old            Old-old       Gender (ref. Male)            Female            Other       Ethnicity (ref. White)            Non-White            Prefer not to say       Deprivation (ref. Quintile 1 - Most deprived)            Quintile 2            Quintile 3            Quintile 4            Quintile 5 – Least deprived       Education (ref. Lower)            Middle            Higher            Other      Physical health (ref. Poor/fair)            Good            Very good/excellent | 0.949 (0.543, 1.661)  1.407 (0.754, 2.625)  2.377 (1.293, 4.372)  3.602 (1.245, 10.424)  0.891 (0.622, 1.275)  102777005.37 (0.000, -)  0.310 (0.171, 0.561)  65510880.614 (0.000, -)  1.071 (0.557, 2.061)  1.294 (0.669, 2.502)  1.535 (0.810, 2.910)  1.369 (0.734, 2.554)  1.461 (0.875, 2.441)  1.809 (1.154, 2.836)  2.008 (0.817, 4.938)  1.217 (0.778, 1.903)  1.253 (0.813, 1.930) | p>0.05  **p<0.01**  p>0.05  **p<0.01**  **p<0.05**  p>0.05  p>0.05  p>0.05  **p<0.001***  **p<0.001***  p>0.05  p>0.05  p>0.05  p>0.05  p>0.05  p>0.05  p>0.05  p>0.05  **p<0.05**  p>0.05  p>0.05  p>0.05  p>0.05 |
| Community based activities | Preventability of cognitive frailty       Age (ref. Young adults)            Middle-aged            Young-old            Old-old       Gender (ref. Male)            Female            Other       Ethnicity (ref. White)            Non-White            Prefer not to say       Deprivation (ref. Quintile 1 - Most deprived)            Quintile 2            Quintile 3            Quintile 4            Quintile 5 – Least deprived       Education (ref. Lower)            Middle            Higher            Other      Physical health (ref. Poor/fair)            Good            Very good/excellent | 1.245 (0.996, 1.556)  1.634 (1.156, 2.310)  3.613 (2.590, 5.039)  5.032 (3.289, 7.699)  0.561 (0.487, 0.647)  1.358 (0.350, 5.267)  0.947 (0.643, 1.396)  1.235 (0.350, 4.358)  0.855 (0.625, 1.168)  1.160 (0.858, 1.567)  1.239 (0.927, 1.657)  1.029 (0.772, 1.371)  1.312 (1.048, 1.643)  1.856 (1.522, 2.263)  1.399 (1.013, 1.932)  1.352 (1.124, 1.627)  1.708 (1.429, 2.042) | p>0.05  **p<0.001***  **p<0.01**  **p<0.001***  **p<0.001***  **p<0.001***  **p<0.001***  p>0.05  p>0.05  p>0.05  p>0.05  **p<0.01**  p>0.05  p>0.05  p>0.05  p>0.05  **p<0.001***  **p<0.05**  **p<0.001***  **p<0.05**  **p<0.001***  **p<0.01***  **p<0.001*** |
| Drinking 2 litres of water | Preventability of cognitive frailty       Age (ref. Young adults)            Middle-aged            Young-old            Old-old       Gender (ref. Male)            Female            Other       Ethnicity (ref. White)            Non-White            Prefer not to say       Deprivation (ref. Quintile 1 - Most deprived)            Quintile 2            Quintile 3            Quintile 4            Quintile 5 – Least deprived       Education (ref. Lower)            Middle            Higher            Other      Physical health (ref. Poor/fair)            Good            Very good/excellent | 1.436 (0.996, 2.070)  0.726 (0.349, 1.511)  0.677 (0.334, 1.371)  0.564 (0.246, 1.293)  0.764 (0.591, 0.987)  0.579 (0.071, 4.752)  1.415 (0.611, 3.275)  0.691 (0.086, 5.519)  0.786 (0.454, 1.360)  1.207 (0.693, 2.100)  1.295 (0.758, 2.211)  1.118 (0.663, 1.884)  0.930 (0.625, 1.384)  1.234 (0.861, 1.770)  1.026 (0.570, 1.845)  0.995 (0.711, 1.392)  1.131 (0.812, 1.575) | p>0.05  p>0.05  p>0.05  p>0.05  p>0.05  p>0.05  **p<0.05**  p>0.05  p>0.05  p>0.05  p>0.05  p>0.05  p>0.05  p>0.05  p>0.05  p>0.05  p>0.05  p>0.05  p>0.05  p>0.05  p>0.05  p>0.05  p>0.05 |
| Drinking alcohol | Preventability of cognitive frailty       Age (ref. Young adults)            Middle-aged            Young-old            Old-old       Gender (ref. Male)            Female            Other       Ethnicity (ref. White)            Non-White            Prefer not to say       Deprivation (ref. Quintile 1 - Most deprived)            Quintile 2            Quintile 3            Quintile 4            Quintile 5 – Least deprived       Education (ref. Lower)            Middle            Higher            Other      Physical health (ref. Poor/fair)            Good            Very good/excellent | 1.003 (0.772, 1.302)  1.263 (0.886, 1.801)  1.563 (1.113, 2.194)  1.601 (1.003, 2.557)  1.704 (1.420, 2.044)  1.843 (0.374, 9.077)  0.401 (0.272, 0.590)  0.386 (0.105, 1.417)  1.123 (0.799, 1.580)  1.527 (1.088, 2.142)  1.306 (0.947, 1.800)  1.439 (1.046, 1.978)  1.036 (0.796, 1.350)  1.098 (0.870, 1.387)  0.916 (0.629, 1.333)  1.769 (1.441, 2.172)  2.085 (1.709, 2.545) | p>0.05  **p<0.05**  p>0.05  **p<0.05**  **p<0.05**  **p<0.001***  **p<0.001***  p>0.05  **p<0.001***  **p<0.001***  p>0.05  **p<0.05**  p>0.05  **p<0.05**  p>0.05  **p<0.05**  p>0.05  p>0.05  p>0.05  p>0.05  **p<0.001***  **p<0.001***  **p<0.001*** |
| Getting enough sleep | Preventability of cognitive frailty       Age (ref. Young adults)            Middle-aged            Young-old            Old-old       Gender (ref. Male)            Female            Other       Ethnicity (ref. White)            Non-White            Prefer not to say       Deprivation (ref. Quintile 1 - Most deprived)            Quintile 2            Quintile 3            Quintile 4            Quintile 5 – Least deprived       Education (ref. Lower)            Middle            Higher            Other      Physical health (ref. Poor/fair)            Good            Very good/excellent | 1.616 (1.049, 2.489)  1.525 (0.761, 3.055)  2.305 (1.177, 4.515)  2.330 (0.936, 5.802)  1.079 (0.745, 1.563)  0.420 (0.045, 3.878)  1.326 (0.471, 3.727)  89153308.364 (0.000, -)  0.641 (0.281, 1.462)  0.609 (0.268, 1.380)  0.601 (0.272, 1.327)  0.581 (0.265, 1.274)  1.647 (0.995, 2.726)  1.721 (1.117, 2.653)  1.513 (0.702, 3.260)  2.119 (1.484, 3.025)  7.857 (4.867, 12.682) | **p<0.05**  **p<0.05**  p>0.05  **p<0.05**  p>0.05  p>0.05  p>0.05  p>0.05  p>0.05  p>0.05  p>0.05  p>0.05  p>0.05  p>0.05  p>0.05  p>0.05  p>0.05  p>0.05  **p<0.05**  p>0.05  **p<0.001***  **p<0.001***  **p<0.001*** |
| Exposure to air pollution | Preventability of cognitive frailty       Age (ref. Young adults)            Middle-aged            Young-old            Old-old       Gender (ref. Male)            Female            Other       Ethnicity (ref. White)            Non-White            Prefer not to say       Deprivation (ref. Quintile 1 - Most deprived)            Quintile 2            Quintile 3            Quintile 4            Quintile 5 – Least deprived       Education (ref. Lower)            Middle            Higher            Other      Physical health (ref. Poor/fair)            Good            Very good/excellent | 1.874 (1.458, 2.408)  1.375 (0.945, 2.000)  1.986 (1.385, 2.849)  1.935 (1.177, 3.181)  0.908 (0.756, 1.092)  2.636 (0.325, 21.402)  1.045 (0.644, 1.696)  0.322 (0.090, 1.150)  0.974 (0.672, 1.410)  1.423 (0.981, 2.063)  1.263 (0.889, 1.796)  1.260 (0.890, 1.784)  1.398 (1.057, 1.849)  1.349 (1.060, 1.716)  1.948 (1.227, 3.093)  0.971 (0.771, 1.223)  1.299 (1.033, 1.634) | **p<0.001***  **p>0.001***  p>0.05  **p<0.001***  **p<0.01**  p>0.05  p>0.05  p>0.05  p>0.05  p>0.05  p>0.05  p>0.05  p>0.05  p>0.05  p>0.05  p>0.05  **p<0.05**  **p<0.05**  **p<0.05**  **p<0.01**  **p<0.01**  p>0.05  **p<0.05** |
| Eating fruits and vegetables | Preventability of cognitive frailty       Age (ref. Young adults)            Middle-aged            Young-old            Old-old       Gender (ref. Male)            Female            Other       Ethnicity (ref. White)            Non-White            Prefer not to say       Deprivation (ref. Quintile 1 - Most deprived)            Quintile 2            Quintile 3            Quintile 4            Quintile 5 – Least deprived       Education (ref. Lower)            Middle            Higher            Other      Physical health (ref. Poor/fair)            Good            Very good/excellent | 0.000 (0.000, -)  0.000 (0.000, -)  0.000 (0.000, -)  0.000 (0.000, -)    0.438 (0.107, 1.799)  222925.360 (0.000, -)  2409106.375 (0.000, -)  2883435.897 (0.000, -)  6400609.286 (0.000, -)  1.896 (0.167, 21.497)  1.830 (0.185, 18.106)  3.253 (0.283, 37.430)  0.000 (0.000, -)  0.000 (0.000, -)  0.000 (0.000, -)  1.381 (0.227, 8.390)  2.018 (0.328, 12.427) | p>0.05  p>0.05  p>0.05  p>0.05  p>0.05  p>0.05  p>0.05  p>0.05  p>0.05  p>0.05  p>0.05  p>0.05  p>0.05  p>0.05  p>0.05  p>0.05  p>0.05  p>0.05  p>0.05  p>0.05  p>0.05  p>0.05  p>0.05 |
| Further education | Preventability of cognitive frailty       Age (ref. Young adults)            Middle-aged            Young-old            Old-old       Gender (ref. Male)            Female            Other       Ethnicity (ref. White)            Non-White            Prefer not to say       Deprivation (ref. Quintile 1 - Most deprived)            Quintile 2            Quintile 3            Quintile 4            Quintile 5 – Least deprived       Education (ref. Lower)            Middle            Higher            Other      Physical health (ref. Poor/fair)            Good            Very good/excellent | 1.161 (0.932, 1.447)  0.678 (0.485, 0.947)  0.634 (0.460, 0.873)  0.759 (0.504, 1.144)  0.753 (0.656, 0.865)  1.036 (0.281, 3.818)  1.741 (1.177, 2.577)  1.202 (0.336, 4.298)  0.822 (0.606, 1.116)  0.846 (0.630, 1.137)  1.014 (0.763, 1.348)  0.854 (0.645, 1.132)  1.468 (1.172, 1.839)  2.406 (1.971, 2.937)  1.527 (1.107, 2.105)  1.169 (0.974, 1.402)  1.253 (1.052, 1.493) | p>0.05  **p<0.05**  **p<0.05**  **p<0.01**  p>0.05  **p<0.001***  **p<0.001***  p>0.05  **p<0.05**  **p<0.01**  p>0.05  p>0.05  p>0.05  p>0.05  p>0.05  p>0.05  **p<0.001***  **p<0.001***  **p<0.001***  **p<0.05**  **p<0.05**  p>0.05  **p<0.05** |
| Using green spaces | Preventability of cognitive frailty       Age (ref. Young adults)            Middle-aged            Young-old            Old-old       Gender (ref. Male)            Female            Other       Ethnicity (ref. White)            Non-White            Prefer not to say       Deprivation (ref. Quintile 1 - Most deprived)            Quintile 2            Quintile 3            Quintile 4            Quintile 5 – Least deprived       Education (ref. Lower)            Middle            Higher            Other      Physical health (ref. Poor/fair)            Good            Very good/excellent | 1.432 (0.811, 2.530)  1.601 (0.734, 3.493)  2.288 (1.074, 4.876)  2.160 (0.727, 6.417)  0.879 (0.563, 1.370)  0.147 (0.025, 0.857)  0.197 (0.102, 0.380)  42241714.096 (0.000, -)  1.229 (0.573, 2.638)  1.299 (0.596, 2.831)  1.463 (0.693, 3.091)  1.571 (0.743, 3.324)  0.975 (0.536, 1.771)  1.892 (1.068, 3.350)  1.841 (0.613, 5.533)  2.250 (1.417, 3.572)  4.961 (2.885, 8.531) | p>0.05  p>0.05  p>0.05  **p<0.05**  p>0.05  p>0.05  p>0.05  **p<0.05**  **p<0.001***  **p<0.001***  p>0.05  p>0.05  p>0.05  p>0.05  p>0.05  p>0.05  **p<0.05**  p>0.05  **p<0.05**  p>0.05  **p<0.001***  **p<0.001***  **p<0.001*** |
| Wearing hearing aids | Preventability of cognitive frailty       Age (ref. Young adults)            Middle-aged            Young-old            Old-old       Gender (ref. Male)            Female            Other       Ethnicity (ref. White)            Non-White            Prefer not to say       Deprivation (ref. Quintile 1 - Most deprived)            Quintile 2            Quintile 3            Quintile 4            Quintile 5 – Least deprived       Education (ref. Lower)            Middle            Higher            Other      Physical health (ref. Poor/fair)            Good            Very good/excellent | 0.996 (0.758, 1.309)  2.245 (0.879, 5.736)  10.196 (4.124, 25.207)  34.834 (13.626, 89.048)  1.607 (1.359, 1.900)  2.271 (0.386, 13.349)  0.559 (0.298, 1.051)  2.777 (0.718, 10.748)  0.919 (0.613, 1.377)  0.821 (0.556, 1.212)  0.880 (0.604, 1.280)  0.863 (0.595, 1.250)  1.173 (0.890, 1.546)  1.016 (0.795, 1.299)  1.009 (0.679, 1.500)  0.840 (0.671, 1.052)  0.721 (0.579, 0.898) | p>0.05  **p<0.001***  p>0.05  **p<0.001***  **p<0.001***  **p<0.001***  **p<0.001***  p>0.05  p>0.05  p>0.05  p>0.05  p>0.05  p>0.05  p>0.05  p>0.05  p>0.05  p>0.05  p>0.05  p>0.05  p>0.05  **p<0.05**  p>0.05  **p<0.01** |
| Taking illegal substances | Preventability of cognitive frailty       Age (ref. Young adults)            Middle-aged            Young-old            Old-old       Gender (ref. Male)            Female            Other       Ethnicity (ref. White)            Non-White            Prefer not to say       Deprivation (ref. Quintile 1 - Most deprived)            Quintile 2            Quintile 3            Quintile 4            Quintile 5 – Least deprived       Education (ref. Lower)            Middle            Higher            Other      Physical health (ref. Poor/fair)            Good            Very good/excellent | 1.450 (0.656, 3.206)  0.669 (0.325, 1.379)  0.325 (0.159, 0.667)  0.366 (0.119, 1.124)  1.789 (1.170, 2.735)  0.000 (0.000, -)  1.646 (0.722, 3.750)  0.000 (0.000, -)  0.660 (0.326, 1.335)  0.358 (0.165, 0.775)  0.374 (0.184, 0.761)  0.417 (0.210, 0.828)  1.282 (0.576, 2.850)  1.449 (0.704, 2.982)  1.188 (0.359, 3.930)  0.771 (0.451, 1.316)  0.600 (0.352, 1.022) | p>0.05  **p<0.01**  p>0.05  **p<0.01**  p>0.05  **p<0.05**  **p<0.01**  p>0.05  p>0.05  p>0.05  p>0.05  **p<0.05**  p>0.05  **p<0.01**  **p<0.01**  **p<0.05**  p>0.05  p>0.05  p>0.05  p>0.05  p>0.05  p>0.05  p>0.05 |
| Managing mental wellbeing | Preventability of cognitive frailty       Age (ref. Young adults)            Middle-aged            Young-old            Old-old       Gender (ref. Male)            Female            Other       Ethnicity (ref. White)            Non-White            Prefer not to say       Deprivation (ref. Quintile 1 - Most deprived)            Quintile 2            Quintile 3            Quintile 4            Quintile 5 – Least deprived       Education (ref. Lower)            Middle            Higher            Other      Physical health (ref. Poor/fair)            Good            Very good/excellent | 1.695 (1.153, 2.491)  0.573 (0.221, 1.487)  0.525 (0.208, 1.325)  0.515 (0.181, 1.471)  0.555 (0.420, 0.732)  0.296 (0.036, 2.464)  1.246 (0.498, 3.117)  0.277 (0.057, 1.354)  0.574 (0.270, 1.221)  0.705 (0.334, 1.486)  0.608 (0.296, 1.249)  0.598 (0.293, 1.221)  1.158 (0.748, 1.792)  1.329 (0.903, 1.954)  1.574 (0.767, 3.232)  0.968 (0.670, 1.398)  1.265 (0.873, 1.832) | **p<0.01**  p>0.05  p>0.05  p>0.05  p>0.05  **p<0.001***  **p<0.001***  p>0.05  p>0.05  p>0.05  p>0.05  p>0.05  p>0.05  p>0.05  p>0.05  p>0.05  p>0.05  p>0.05  p>0.05  p>0.05  p>0.05  p>0.05  p>0.05 |
| Managing weight | Preventability of cognitive frailty       Age (ref. Young adults)            Middle-aged            Young-old            Old-old       Gender (ref. Male)            Female            Other       Ethnicity (ref. White)            Non-White            Prefer not to say       Deprivation (ref. Quintile 1 - Most deprived)            Quintile 2            Quintile 3            Quintile 4            Quintile 5 – Least deprived       Education (ref. Lower)            Middle            Higher            Other      Physical health (ref. Poor/fair)            Good            Very good/excellent | 1.587 (1.147, 2.197)  1.485 (0.918, 2.402)  2.061 (1.298, 3.272)  1.507 (0.821, 2.768)  0.886 (0.693, 1.132)  1.119 (0.135, 9.260)  0.681 (0.391, 1.186)  164117778.81 (0.000, -)  0.704 (0.417, 1.189)  0.996 (0.584, 1.697)  0.867 (0.523, 1.439)  0.850 (0.515, 1.403)  1.194 (0.820, 1.739)  1.209 (0.871, 1.679)  1.139 (0.655, 1.983)  1.359 (1.025, 1.801)  2.189 (1.635, 2.933) | **p<0.01**  **p<0.01**  p>0.05  **p<0.01**  p>0.05  p>0.05  p>0.05  p>0.05  p>0.05  p>0.05  p>0.05  p>0.05  p>0.05  p>0.05  p>0.05  p>0.05  p>0.05  p>0.05  p>0.05  p>0.05  **p<0.001***  **p<0.05**  **p<0.001*** |
| Mindful Activities | Preventability of cognitive frailty       Age (ref. Young adults)            Middle-aged            Young-old            Old-old       Gender (ref. Male)            Female            Other       Ethnicity (ref. White)            Non-White            Prefer not to say       Deprivation (ref. Quintile 1 - Most deprived)            Quintile 2            Quintile 3            Quintile 4            Quintile 5 – Least deprived       Education (ref. Lower)            Middle            Higher            Other      Physical health (ref. Poor/fair)            Good            Very good/excellent | 1.360 (1.091, 1.694)  1.101 (0.797, 1.523)  1.300 (0.953, 1.773)  1.493 (0.996, 2.236)  0.455 (0.396, 0.524)  1.913 (0.480, 7.617)  2.141 (1.437, 3.190)  1.104 (0.308, 3.954)  0.787 (0.580, 1.068)  0.932 (0.694, 1.252)  0.999 (0.752, 1.327)  0.919 (0.694, 1.218)  1.177 (0.943, 1.468)  1.568 (1.291, 1.906)  1.496 (1.086, 2.061)  0.934 (0.778, 1.121)  0.861 (0.722, 1.026) | **p<0.01**  **p<0.05**  p>0.05  p>0.05  p>0.05  **p<0.001***  **p<0.001***  p>0.05  **p<0.001***  **p<0.001***  p>0.05  p>0.05  p>0.05  p>0.05  p>0.05  p>0.05  **p<0.001***  p>0.05  **p<0.001***  **p<0.05**  p>0.05  p>0.05  p>0.05 |
| Being near water | Preventability of cognitive frailty       Age (ref. Young adults)            Middle-aged            Young-old            Old-old       Gender (ref. Male)            Female            Other       Ethnicity (ref. White)            Non-White            Prefer not to say       Deprivation (ref. Quintile 1 - Most deprived)            Quintile 2            Quintile 3            Quintile 4            Quintile 5 – Least deprived       Education (ref. Lower)            Middle            Higher            Other      Physical health (ref. Poor/fair)            Good            Very good/excellent | 1.498 (1.156, 1.940)  1.108 (0.750, 1.635)  1.624 (1.115, 2.365)  1.109 (0.683, 1.801)  1.011 (0.838, 1.220)  0.628 (0.155, 2.540)  0.473 (0.314, 0.714)  1.963 (0.247, 15.628)  1.292 (0.894, 1.868)  1.564 (1.086, 2.253)  1.641 (1.158, 2.327)  1.260 (0.898, 1.768)  1.280 (0.962, 1.703)  1.235 (0.964, 1.581)  1.413 (0.913, 2.188)  1.577 (1.268, 1.961)  2.306 (1.852, 2.870) | **p<0.01**  **p<0.001***  p>0.05  **p<0.05**  p>0.05  p>0.05  p>0.05  p>0.05  **p<0.01***  **p<0.001***  p>0.05  **p<0.05**  p>0.05  **p<0.05**  **p<0.01**  p>0.05  p>0.05  p>0.05  p>0.05  p>0.05  **p<0.001***  **p<0.001***  **p<0.001*** |
| Eating nuts, seeds and legumes | Preventability of cognitive frailty       Age (ref. Young adults)            Middle-aged            Young-old            Old-old       Gender (ref. Male)            Female            Other       Ethnicity (ref. White)            Non-White            Prefer not to say       Deprivation (ref. Quintile 1 - Most deprived)            Quintile 2            Quintile 3            Quintile 4            Quintile 5 – Least deprived       Education (ref. Lower)            Middle            Higher            Other      Physical health (ref. Poor/fair)            Good            Very good/excellent | 1.834 (1.194, 2.817)  2.976 (1.655, 5.353)  4.778 (2.722, 8.386)  15.619 (4.397, 55.483)  0.701 (0.492, 0.998)  181368204.37 (0.000, -)  1.561 (0.547, 4.451)  71947806.544 (0.000, -)  1.106 (0.604, 2.023)  1.712 (0.902, 3.251)  1.964 (1.066, 3.616)  1.485 (0.829, 2.660)  1.201 (0.771, 1.872)  3.114 (2.000, 4.849)  2.486 (1.022, 6.044)  1.745 (1.183, 2.574)  3.187 (2.088, 4.865) | **p<0.01**  **p<0.001***  **p<0.001***  **p<0.001***  **p<0.001***  p>0.05  **p<0.05**  p>0.05  p>0.05  p>0.05  p>0.05  p>0.05  p>0.05  p>0.05  **p<0.05**  p>0.05  **p<0.001***  p<0.05  **p<0.001***  **p<0.05**  **p<0.001***  **p<0.01**  **p<0.001*** |
| Eating processed foods | Preventability of cognitive frailty       Age (ref. Young adults)            Middle-aged            Young-old            Old-old       Gender (ref. Male)            Female            Other       Ethnicity (ref. White)            Non-White            Prefer not to say       Deprivation (ref. Quintile 1 - Most deprived)            Quintile 2            Quintile 3            Quintile 4            Quintile 5 – Least deprived       Education (ref. Lower)            Middle            Higher            Other      Physical health (ref. Poor/fair)            Good            Very good/excellent | 1.120 (0.756, 1.660)  0.146 (0.035, 0.609)  0.100 (0.024, 0.408)  0.065 (0.015, 0.280)  1.895 (1.421, 2.528)  102574133.59 (0.000, -)  0.376 (0.215, 0.660)  0.595 (0.074, 4.790)  0.957 (0.532, 1.721)  0.939 (0.534, 1.649)  0.993 (0.574, 1.718)  1.030 (0.598, 1.773)  1.007 (0.681, 1.490)  1.182 (0.837, 1.670)  0.832 (0.493, 1.405)  1.059 (0.756, 1.483)  0.978 (0.708, 1.350) | p>0.05  **p<0.001***  **p<0.01**  **p<0.01***  **p<0.001***  **p<0.001***  **p<0.001***  p>0.05  **p<0.01**  **p<0.001***  p>0.05  p>0.05  p>0.05  p>0.05  p>0.05  p>0.05  p>0.05  p>0.05  p>0.05  p>0.05  p>0.05  p>0.05  p>0.05 |
| Protecting against hearing loss | Preventability of cognitive frailty       Age (ref. Young adults)            Middle-aged            Young-old            Old-old       Gender (ref. Male)            Female            Other       Ethnicity (ref. White)            Non-White            Prefer not to say       Deprivation (ref. Quintile 1 - Most deprived)            Quintile 2            Quintile 3            Quintile 4            Quintile 5 – Least deprived       Education (ref. Lower)            Middle            Higher            Other      Physical health (ref. Poor/fair)            Good            Very good/excellent | 1.063 (0.847, 1.334)  0.969 (0.685, 1.371)  1.240 (0.891, 1.726)  1.278 (0.839, 1.948)  1.362 (1.183, 1.567)  1.569 (0.435, 5.651)  0.998 (0.674, 1.477)  1.861 (0.531, 6.524)  0.925 (0.671, 1.274)  1.125 (0.827, 1.528)  1.209 (0.899, 1.625)  1.014 (0.756, 1.360)  1.090 (0.868, 1.369)  1.008 (0.824, 1.232)  0.987 (0.708, 1.375)  1.080 (0.893, 1.307)  1.186 (0.988, 1.423) | p>0.05  **p<0.05**  p>0.05  p>0.05  p>0.05  **p<0.001***  **p<0.001***  p>0.05  p>0.05  p>0.05  p>0.05  p>0.05  p>0.05  p>0.05  p>0.05  p>0.05  p>0.05  p>0.05  p>0.05  p>0.05  p>0.05  p>0.05  p>0.05 |
| Purpose in life | Preventability of cognitive frailty       Age (ref. Young adults)            Middle-aged            Young-old            Old-old       Gender (ref. Male)            Female            Other       Ethnicity (ref. White)            Non-White            Prefer not to say       Deprivation (ref. Quintile 1 - Most deprived)            Quintile 2            Quintile 3            Quintile 4            Quintile 5 – Least deprived       Education (ref. Lower)            Middle            Higher            Other      Physical health (ref. Poor/fair)            Good            Very good/excellent | 3.202 (2.128, 4.819)  1.152 (0.515, 2.574)  1.419 (0.652, 3.086)  2.086 (0.711, 6.122)  0.619 (0.433, 0.885)  641195910.733 (0.000, -)  0.881 (0.344, 2.254)  0.364 (0.045, 2.962)  1.104 (0.550, 2.215)  1.753 (0.846, 3.630)  1.514 (0.773, 2.967)  1.402 (0.726, 2.710)  0.968 (0.550, 1.704)  0.940 (0.568, 1.556)  2.885 (0.842, 9.882)  2.099 (1.404, 3.138)  3.800 (2.439, 5.921) | **p<0.001***  p>0.05  p>0.05  p>0.05  p>0.05  **p<0.05**  **p<0.01**  p>0.05  p>0.05  p>0.05  p>0.05  p>0.05  p>0.05  p>0.05  p>0.05  p>0.05  p>0.05  p>0.05  p>0.05  p>0.05  **p<0.001***  **p<0.001***  **p<0.001*** |
| Eating red meat | Preventability of cognitive frailty       Age (ref. Young adults)            Middle-aged            Young-old            Old-old       Gender (ref. Male)            Female            Other       Ethnicity (ref. White)            Non-White            Prefer not to say       Deprivation (ref. Quintile 1 - Most deprived)            Quintile 2            Quintile 3            Quintile 4            Quintile 5 – Least deprived       Education (ref. Lower)            Middle            Higher            Other      Physical health (ref. Poor/fair)            Good            Very good/excellent | 0.797 (0.582, 1.091)  0.796 (0.534, 1.188)  0.888 (0.605, 1.304)  1.049 (0.614, 1.792)  1.796 (1.477, 2.184)  1.172 (0.244, 5.622)  0.740 (0.475, 1.152)  0.737 (0.151, 3.581)  0.994 (0.685, 1.442)  1.072 (0.747, 1.538)  1.520 (1.065, 2.170)  1.411 (0.996, 2.001)  0.703 (0.510, 0.969)  0.531 (0.400, 0.706)  0.912 (0.568, 1.465)  0.933 (0.730, 1.191)  0.804 (0.637, 1.015) | p>0.05  p>0.05  p>0.05  p>0.05  p>0.05  **p<0.001***  **p<0.001***  p>0.05  p>0.05  p>0.05  p>0.05  **p<0.01***  p>0.05  p>0.05  **p<0.05**  p>0.05  **p<0.001***  **p<0.05**  **p<0.001***  p>0.05  p>0.05  p>0.05  p>0.05 |
| Smoking or vaping | Preventability of cognitive frailty       Age (ref. Young adults)            Middle-aged            Young-old            Old-old       Gender (ref. Male)            Female            Other       Ethnicity (ref. White)            Non-White            Prefer not to say       Deprivation (ref. Quintile 1 - Most deprived)            Quintile 2            Quintile 3            Quintile 4            Quintile 5 – Least deprived       Education (ref. Lower)            Middle            Higher            Other      Physical health (ref. Poor/fair)            Good            Very good/excellent | 0.984 (0.626, 1.546)  0.408 (0.261, 0.640)  0.132 (0.084, 0.209)  0.111 (0.046, 0.264)  1.187 (0.865, 1.630)  0.000 (0.000, -)  1.045 (0.529, 2.065)  1.941 (0.240, 15.704)  0.592 (0.364, 0.963)  0.416 (0.250, 0.693)  0.367 (0.227, 0.594)  0.366 (0.228, 0.588)  0.691 (0.446, 1.069)  0.484 (0.327, 0.718)  0.641 (0.311, 1.320)  0.556 (0.389, 0.794)  0.432 (0.302, 0.616) | p>0.05  **p<0.001***  **p<0.001***  **p<0.001***  **p<0.001***  p>0.05  p>0.05  p>0.05  p>0.05  p>0.05  p>0.05  **p<0.001***  **p<0.05**  **p<0.001***  **p<0.001***  **p<0.001***  **p<0.01**  p>0.05  **p<0.001***  p>0.05  **p<0.001***  **p<0.01***  **p<0.001*** |
| Socialising | Preventability of cognitive frailty       Age (ref. Young adults)            Middle-aged            Young-old            Old-old       Gender (ref. Male)            Female            Other       Ethnicity (ref. White)            Non-White            Prefer not to say       Deprivation (ref. Quintile 1 - Most deprived)            Quintile 2            Quintile 3            Quintile 4            Quintile 5 – Least deprived       Education (ref. Lower)            Middle            Higher            Other      Physical health (ref. Poor/fair)            Good            Very good/excellent | 1.758 (1.116, 2.770)  0.366 (0.106, 1.262)  0.339 (0.101, 1.141)  0.442 (0.111, 1.750)  0.474 (0.338, 0.664)  0.191 (0.022, 1.630)  0.455 (0.220, 0.939)  83446193.710 (0.000, -)  1.217 (0.620, 2.386)  1.642 (0.832, 3.241)  1.844 (0.957, 3.553)  1.614 (0.854, 3.048)  1.094 (0.632, 1.893)  1.033 (0.636, 1.678)  3.182 (0.939, 10.787)  1.625 (1.093, 2.417)  2.975 (1.933, 4.579) | **p<0.05**  p>0.05  p>0.05  p>0.05  p>0.05  **p<0.001***  **p<0.001***  p>0.05  p>0.05  **p<0.05**  p>0.05  p>0.05  p>0.05  p>0.05  p>0.05  p>0.05  p>0.05  p>0.05  p>0.05  p>0.05  **p<0.001***  **p<0.05**  **p<0.001*** |
| Strength training | Preventability of cognitive frailty       Age (ref. Young adults)            Middle-aged            Young-old            Old-old       Gender (ref. Male)            Female            Other       Ethnicity (ref. White)            Non-White            Prefer not to say       Deprivation (ref. Quintile 1 - Most deprived)            Quintile 2            Quintile 3            Quintile 4            Quintile 5 – Least deprived       Education (ref. Lower)            Middle            Higher            Other      Physical health (ref. Poor/fair)            Good            Very good/excellent | 1.755 (1.397, 2.206)  0.977 (0.704, 1.356)  0.931 (0.681, 1.274)  0.483 (0.318, 0.732)  0.957 (0.832, 1.100)  4.453 (0.916, 21.649)  1.351 (0.923, 1.978)  1.216 (0.346, 4.276)  1.289 (0.944, 1.759)  1.336 (0.989, 1.803)  1.463 (1.095, 1.955)  1.652 (1.240, 2.201)  1.300 (1.038, 1.627)  1.519 (1.246, 1.851)  1.492 (1.077, 2.065)  1.477 (1.227, 1.779)  2.515 (2.103, 3.007) | **p<0.001***  **p<0.001***  p>0.05  p>0.05  **p<0.001***  p>0.05  p>0.05  p>0.05  p>0.05  p>0.05  p>0.05  **p<0.01**  p>0.05  p>0.05  **p<0.05**  **p<0.001***  **p<0.001***  **p<0.05**  **p<0.001***  **p<0.05**  **p<0.001***  **p<0.001***  **p<0.001*** |
| Taking vitamins and supplements | Preventability of cognitive frailty       Age (ref. Young adults)            Middle-aged            Young-old            Old-old       Gender (ref. Male)            Female            Other       Ethnicity (ref. White)            Non-White            Prefer not to say       Deprivation (ref. Quintile 1 - Most deprived)            Quintile 2            Quintile 3            Quintile 4            Quintile 5 – Least deprived       Education (ref. Lower)            Middle            Higher            Other      Physical health (ref. Poor/fair)            Good            Very good/excellent | 1.174 (0.921, 1.495)  1.070 (0.736, 1.556)  1.138 (0.795, 1.628)  1.123 (0.708, 1.781)  0.635 (0.544, 0.740)  1.096 (0.228, 5.265)  1.250 (0.793, 1.971)  1.350 (0.282, 6.464)  0.658 (0.453, 0.954)  0.622 (0.434, 0.892)  0.720 (0.507, 1.023)  0.732 (0.517, 1.037)  1.280 (1.000, 1.639)  1.292 (1.042, 1.603)  1.456 (1.000, 2.120)  1.058 (0.856, 1.308)  0.858 (0.701, 1.049) | p>0.05  p>0.05  p>0.05  p>0.05  p>0.05  **p<0.001***  **p<0.001***  p>0.05  p>0.05  p>0.05  p>0.05  p>0.05  **p<0.05**  **p<0.05**  p>0.05  p>0.05  p>0.05  p>0.05  **p<0.05**  p>0.05  **p<0.05**  p>0.05  p>0.05 |
| Volunteering and helping others | Preventability of cognitive frailty       Age (ref. Young adults)            Middle-aged            Young-old            Old-old       Gender (ref. Male)            Female            Other       Ethnicity (ref. White)            Non-White            Prefer not to say       Deprivation (ref. Quintile 1 - Most deprived)            Quintile 2            Quintile 3            Quintile 4            Quintile 5 – Least deprived       Education (ref. Lower)            Middle            Higher            Other      Physical health (ref. Poor/fair)            Good            Very good/excellent | 1.543 (1.226, 1.942)  1.361 (0.973, 1.905)  2.369 (1.712, 3.278)  2.917 (1.864, 4.566)  0.555 (0.476, 0.647)  0.424 (0.113, 1.596)  1.059 (0.698, 1.608)  0.967 (0.245, 3.810)  1.028 (0.742, 1.425)  1.319 (0.957, 1.818)  1.257 (0.925, 1.709)  1.353 (0.998, 1.835)  0.965 (0.757, 1.230)  1.340 (1.079, 1.666)  1.465 (1.002, 2.141)  1.517 (1.246, 1.847)  1.770 (1.462, 2.143) | **p<0.001***  **p<0.001***  p>0.05  **p<0.001***  **p<0.001***  **p<0.001***  **p<0.001***  p>0.05  p>0.05  p>0.05  p>0.05  p>0.05  p>0.05  p>0.05  p>0.05  p>0.05  **p<0.001***  p>0.05  **p<0.01**  **p<0.05**  **p<0.001***  **p<0.001***  **p<0.001*** |

*Note.* Bolded comparisons are significant at p<0.05. * denotes comparisons remaining significant after Bonferroni correction at p<0.002.

# Supplementary Table 4: Current behaviours – by importance of factors

| Dependent variable | Predictor and covariates | Odds Ratio (95% CI) | P-value |
| --- | --- | --- | --- |
| Attending places of worship | Importance of attending places of worship       Age (ref. Young adults)            Middle-aged            Young-old            Old-old       Gender (ref. Male)            Female            Other       Ethnicity (ref. White)            Non-White            Prefer not to say      Deprivation (ref. Quintile 1 - Most deprived)            Quintile 2            Quintile 3            Quintile 4            Quintile 5 – Least deprived       Education (ref. Lower)            Middle            Higher            Other      Physical health (ref. Poor/fair)            Good            Very good/excellent | 9.188 (7.752, 10.890)  1.350 (0.833, 2.187)  2.536 (1.598, 4.024)  3.971 (2.284, 6.902)    0.989 (0.819, 1.194)  0.000 (0.000, -)    1.878 (1.208, 2.920)  2.828 (0.673, 11.889)    0.898 (0.594, 1.357)  0.976 (0.657, 1.451)  0.952 (0.650, 1.395)  0.944 (0.647, 1.376)    0.818 (0.605, 1.107)  1.115 (0.863, 1.441)  1.603 (1.079, 2.382)    1.138 (0.893, 1.449)  1.014 (0.803, 1.281) | **p<0.001***  **p<0.001***  p>0.05  **p<0.001***  **p<0.001***  p>0.05  p>0.05  p>0.05  **p<0.01**  **p<0.01**  p>0.05  p>0.05  p>0.05  p>0.05  p>0.05  p>0.05  **p<0.01**  p>0.05  p>0.05  **p<0.05**  p>0.05  p>0.05  p>0.05 |
| Balance exercises | Importance of balance exercises       Age (ref. Young adults)            Middle-aged            Young-old            Old-old       Gender (ref. Male)            Female            Other       Ethnicity (ref. White)            Non-White            Prefer not to say       Deprivation (ref. Quintile 1 - Most deprived)            Quintile 2            Quintile 3            Quintile 4            Quintile 5 – Least deprived       Education (ref. Lower)            Middle            Higher            Other      Physical health (ref. Poor/fair)            Good            Very good/excellent | 8.184 (6.486, 10.327)  1.424 (1.014, 2.000)  2.704 (1.949, 3.752)  3.358 (2.156, 5.232)    0.440 (0.378, 0.513)  1.628 (0.373, 7.105)    1.271 (0.840, 1.925)  2.249 (0.509, 9.938)    1.263 (0.910, 1.754)  1.462 (1.063, 2.011)  1.436 (1.057, 1.951)  1.608 (1.187, 2.179)    1.243 (0.979, 1.580)  1.894 (1.533, 2.341)  1.356 (0.957, 1.922)    1.511 (1.242, 1.837)  2.140 (1.771, 2.586) | **p<0.001***  **p<0.001***  **p<0.05**  **p<0.001***  **p<0.001***  **p<0.001***  **p<0.001***  p>0.05  p>0.05  p>0.05  p>0.05  **p<0.05**  p>0.05  **p<0.05**  **p<0.05**  **p<0.01**  **p<0.001***  p>0.05  **p<0.001***  p>0.05  **p<0.001***  **p<0.001***  **p<0.001*** |
| Book club | Importance of book club       Age (ref. Young adults)            Middle-aged            Young-old            Old-old       Gender (ref. Male)            Female            Other       Ethnicity (ref. White)            Non-White            Prefer not to say       Deprivation (ref. Quintile 1 - Most deprived)            Quintile 2            Quintile 3            Quintile 4            Quintile 5 – Least deprived       Education (ref. Lower)            Middle            Higher            Other      Physical health (ref. Poor/fair)            Good            Very good/excellent | 4.321 (3.602, 5.184)  1.213 (0.742, 1.983)  2.275 (1.429, 3.623)  3.072 (1.740, 5.426)    0.526 (0.426, 0.649)  0.404 (0.043, 3.778)    1.981 (1.251, 3.137)  6.203 (1.628, 23.629)    1.280 (0.797, 2.055)  1.491 (0.951, 2.339)  1.292 (0.832, 2.006)  1.591 (1.032, 2.452)    1.029 (0.743, 1.425)  1.686 (1.280, 2.221)  1.371 (0.884, 2.126)    1.345 (1.032, 1.753)  1.493 (1.161, 1.921) | **p<0.001***  **p<0.001***  p>0.05  **p<0.001***  **p<0.001***  **p<0.001***  **p<0.001***  p>0.05  **p<0.001***  **p<0.01**  **p<0.01**  p>0.05  p>0.05  p>0.05  p>0.05  **p<0.05**  **p<0.001***  p>0.05  **p<0.001***  p>0.05  **p<0.01**  **p<0.05**  **p<0.01** |
| Cardiovascular exercises | Importance of cardiovascular exercises       Age (ref. Young adults)            Middle-aged            Young-old            Old-old       Gender (ref. Male)            Female            Other       Ethnicity (ref. White)            Non-White            Prefer not to say       Deprivation (ref. Quintile 1 - Most deprived)            Quintile 2            Quintile 3            Quintile 4            Quintile 5 – Least deprived       Education (ref. Lower)            Middle            Higher            Other      Physical health (ref. Poor/fair)            Good            Very good/excellent | 6.965 (5.746, 8.441)  0.901 (0.615, 1.318)  0.810 (0.562, 1.167)  0.489 (0.308, 0.777)  1.829 (1.549, 2.158)  1.087 (0.264, 4.480)  0.900 (0.586, 1.381)  0.397 (0.102, 1.551)  1.039 (0.738, 1.462)  1.267 (0.908, 1.769)  1.273 (0.923, 1.756)  1.349 (0.981, 1.855)  1.114 (0.875, 1.418)  1.820 (1.471, 2.253)  1.516 (1.059, 2.168)  1.863 (1.532, 2.265)  3.665 (3.019, 4.449) | **p<0.001***  **p<0.01**  p>0.05  p>0.05  **p<0.01**  **p<0.001***  **p<0.001***  p>0.05  p>0.05  p>0.05  p>0.05  p>0.05  p>0.05  p<0.05  p>0.05  p>0.05  **p<0.001***  p>0.05  **p<0.001***  **p<0.05**  **p<0.001***  **p<0.001***  **p<0.001*** |
| Challenging brain | Importance of challenging brain       Age (ref. Young adults)            Middle-aged            Young-old            Old-old       Gender (ref. Male)            Female            Other       Ethnicity (ref. White)            Non-White            Prefer not to say       Deprivation (ref. Quintile 1 - Most deprived)            Quintile 2            Quintile 3            Quintile 4            Quintile 5 – Least deprived       Education (ref. Lower)            Middle            Higher            Other      Physical health (ref. Poor/fair)            Good            Very good/excellent | 6.583 (3.959, 10.946)  1.311 (0.694, 2.479)  2.148 (1.154, 4.000)  3.219 (1.103, 9.401)  1.027 (0.710, 1.486)  140711720.99 (0.000, -)  0.319 (0.174, 0.586)  55370255.068 (0.000, -)  0.988 (0.508, 1.923)  1.226 (0.628, 2.395)  1.497 (0.783, 2.864)  1.364 (0.724, 2.571)  1.458 (0.871, 2.442)  1.879 (1.1199, 2.946)  1.974 (0.798, 4.883)  1.152 (0.733, 1.810)  1.187 (0.769, 1.833) | **p<0.001***  **p<0.01**  p>0.05  **p<0.05**  **p<0.05**  p>0.05  p>0.05  p>0.05  **p<0.01***  **p<0.001***  p>0.05  p>0.05  p>0.05  p>0.05  p>0.05  p>0.05  **p<0.05**  p>0.05  **p<0.01**  p>0.05  p>0.05  p>0.05  p>0.05 |
| Community based activities | Importance of community based activities       Age (ref. Young adults)            Middle-aged            Young-old            Old-old       Gender (ref. Male)            Female            Other       Ethnicity (ref. White)            Non-White            Prefer not to say       Deprivation (ref. Quintile 1 - Most deprived)            Quintile 2            Quintile 3            Quintile 4            Quintile 5 – Least deprived       Education (ref. Lower)            Middle            Higher            Other      Physical health (ref. Poor/fair)            Good            Very good/excellent | 4.452 (3.707, 5.347)  1.662 (1.166, 2.368)  3.735 (2.657, 5.249)  5.100 (3.294, 7.896)  0.623 (0.537, 0.721)  1.245 (0.328, 4.721)  0.911 (0.612, 1.358)  1.976 (0.534, 7.317)  0.903 (0.653, 1.248)  1.197 (0.877, 1.634)  1.221 (0.905, 1.648)  1.032 (0.767, 1.388)  1.291 (1.023, 1.629)  1.798 (1.465, 2.207)  1.320 (0.944, 1.846)  1.313 (1.084, 1.591)  1.653 (1.375, 1.986) | **p<0.001***  **p<0.001***  **p<0.01**  **p<0.001***  **p<0.001***  **p<0.001***  **p<0.001***  p>0.05  p>0.05  p>0.05  p>0.05  **p<0.05**  p>0.05  p>0.05  p>0.05  p>0.05  **p<0.001***  **p<0.05**  **p<0.001***  p>0.05  **p<0.001***  **p<0.01**  **p<0.001*** |
| Drinking 2 litres of water | Importance of drinking 2 litres of water       Age (ref. Young adults)            Middle-aged            Young-old            Old-old       Gender (ref. Male)            Female            Other       Ethnicity (ref. White)            Non-White            Prefer not to say       Deprivation (ref. Quintile 1 - Most deprived)            Quintile 2            Quintile 3            Quintile 4            Quintile 5 – Least deprived       Education (ref. Lower)            Middle            Higher            Other      Physical health (ref. Poor/fair)            Good            Very good/excellent | 8.686 (6.552, 11.516)  0.797 (0.377, 1.683)  0.829 (0.403, 1.706)  0.886 (0.375, 2.091)  0.969 (0.738, 1.272)  0.655 (0.071, 6.002)  1.259 (0.533, 2.975)  0.737 (0.079, 6.849)  0.793 (0.448, 1.406)  1.184 (0.665, 2.108)  1.219 (0.700, 2.126)  1.092 (0.634, 1.880)  0.968 (0.642, 1.460)  1.569 (1.080, 2.280)  1.065 (0.578, 1.963)  0.966 (0.681, 1.371)  1.121 (0.795, 1.580) | **p<0.001***  p>0.05  p>0.05  p>0.05  p>0.05  p>0.05  p>0.05  p>0.05  p>0.05  p>0.05  p>0.05  p>0.05  p>0.05  p>0.05  p>0.05  p>0.05  **p<0.01**  p>0.05  **p<0.05**  p>0.05  p>0.05  p>0.05  p>0.05 |
| Drinking alcohol | Importance of drinking less or no alcohol       Age (ref. Young adults)            Middle-aged            Young-old            Old-old       Gender (ref. Male)            Female            Other       Ethnicity (ref. White)            Non-White            Prefer not to say       Deprivation (ref. Quintile 1 - Most deprived)            Quintile 2            Quintile 3            Quintile 4            Quintile 5 – Least deprived       Education (ref. Lower)            Middle            Higher            Other      Physical health (ref. Poor/fair)            Good            Very good/excellent | 0.386 (0.251, 0.595)  1.273 (0.892, 1.816)  1.552 (1.105, 2.181)  1.527 (0.954, 2.444)  1.628 (1.356, 1.955)  1.813 (0.364, 9.025)  0.409 (0.278, 0.603)  0.408 (0.111, 1.496)  1.120 (0.796, 1.577)  1.521 (1.083, 2.137)  1.302 (0.944, 1.797)  1.434 (1.041, 1.973)  1.043 (0.801, 1.358)  1.102 (0.874, 1.389)  0.936 (0.643, 1.364)  1.778 (1.448, 2.184)  2.103 (1.725, 2.564) | **p<0.001***  **p<0.05**  p>0.05  **p<0.05**  p>0.05  **p<0.001***  **p<0.001***  p>0.05  **p<0.001***  **p<0.001***  p>0.05  **p<0.05**  p>0.05  **p<0.05**  p>0.05  **p<0.05**  p>0.05  p>0.05  p>0.05  p>0.05  **p<0.001***  **p<0.001***  **p<0.001*** |
| Getting enough sleep | Importance of getting enough sleep       Age (ref. Young adults)            Middle-aged            Young-old            Old-old       Gender (ref. Male)            Female            Other       Ethnicity (ref. White)            Non-White            Prefer not to say       Deprivation (ref. Quintile 1 - Most deprived)            Quintile 2            Quintile 3            Quintile 4            Quintile 5 – Least deprived       Education (ref. Lower)            Middle            Higher            Other      Physical health (ref. Poor/fair)            Good            Very good/excellent | 1.942 (0.571, 6.598)  1.542 (0.771, 3.087)  2.345 (1.198, 4.588)  2.317 (0.932, 5.763)  1.057 (0.730, 1.529)  0.435 (0.047, 4.069)  1.367 (0.487, 3.840)  95881932.534 (0.000, -)  0.648 (0.284, 1.479)  0.615 (0.271, 1.395)  0.604 (0.274, 1.334)  0.587 (0.268, 1.288)  1.738 (1.054, 2.867)  1.848 (1.206, 2.832)  1.516 (0.705, 3.258)  2.192 (1.538, 3.124)  8.229 (5.113, 13.244) | p>0.05  **p<0.05**  p>0.05  **p<0.05**  p>0.05  p>0.05  p>0.05  p>0.05  p>0.05  p>0.05  p>0.05  p>0.05  p>0.05  p>0.05  p>0.05  p>0.05  **p<0.05**  **p<0.05**  **p<0.01**  p>0.05  **p<0.001***  **p<0.001***  **p<0.001*** |
| Exposure to air pollution | Importance of limiting exposure to air pollution       Age (ref. Young adults)            Middle-aged            Young-old            Old-old       Gender (ref. Male)            Female            Other       Ethnicity (ref. White)            Non-White            Prefer not to say       Deprivation (ref. Quintile 1 - Most deprived)            Quintile 2            Quintile 3            Quintile 4            Quintile 5 – Least deprived       Education (ref. Lower)            Middle            Higher            Other      Physical health (ref. Poor/fair)            Good            Very good/excellent | 4.067 (3.325, 4.975)  1.424 (0.967, 2.097)  1.978 (1.364, 2.868)  1.920 (1.153, 3.198)  0.999 (0.827, 1.208)  2.845 (0.325, 24.895)  1.042 (0.634, 1.713)  0.302 (0.082, 1.116)  0.986 (0.675, 1.441)  1.441 (0.985, 2.107)  1.324 (0.923, 1.897)  1.292 (0.906, 1.843)  1.464 (1.101, 1.946)  1.492 (1.169, 1.905)  1.983 (1.237, 3.179)  0.942 (0.743, 1.192)  1.303 (1.032, 1.645) | **p<0.001***  **p<0.001***  p>0.05  **p<0.001***  **p<0.05**  p>0.05  p>0.05  p>0.05  p>0.05  p>0.05  p>0.05  p>0.05  p>0.05  p>0.05  p>0.05  p>0.05  **p<0.01**  **p<0.01**  **p<0.01***  **p<0.01**  **p<0.01**  p>0.05  **p<0.05** |
| Eating fruits and vegetables | Importance of eating fruits and vegetables       Age (ref. Young adults)            Middle-aged            Young-old            Old-old       Gender (ref. Male)            Female            Other       Ethnicity (ref. White)            Non-White            Prefer not to say       Deprivation (ref. Quintile 1 - Most deprived)            Quintile 2            Quintile 3            Quintile 4            Quintile 5 – Least deprived       Education (ref. Lower)            Middle            Higher            Other      Physical health (ref. Poor/fair)            Good            Very good/excellent | 4.934 (0.549, 44.297)  0.000 (0.000, -)  0.000 (0.000, -)  0.000 (0.000, -)    0.484 (0.116, 2.023)  413397.649 (0.000, -)  2114499.564 (0.000, -)  2360316.095 (0.000, -)  5779051.321 (0.000, -)  1.543 (0.132, 18.078)  1.500 (0.147, 15.312)  2.825 (0.244, 32.704)  0.000 (0.000, -)  0.000 (0.000, -)  0.000 (0.000, -)  1.160 (0.188, 7.143)  1.607 (0.256, 10.072) | p>0.05  p>0.05  p>0.05  p>0.05  p>0.05  p>0.05  p>0.05  p>0.05  p>0.05  p>0.05  p>0.05  p>0.05  p>0.05  p>0.05  p>0.05  p>0.05  p>0.05  p>0.05  p>0.05  p>0.05  p>0.05  p>0.05  p>0.05 |
| Further education | Importance of further education       Age (ref. Young adults)            Middle-aged            Young-old            Old-old       Gender (ref. Male)            Female            Other       Ethnicity (ref. White)            Non-White            Prefer not to say       Deprivation (ref. Quintile 1 - Most deprived)            Quintile 2            Quintile 3            Quintile 4            Quintile 5 – Least deprived       Education (ref. Lower)            Middle            Higher            Other      Physical health (ref. Poor/fair)            Good            Very good/excellent | 3.497 (2.992, 4.087)  0.693 (0.491, 0.978)  0.612 (0.440, 0.852)  0.706 (0.463, 1.077)  0.808 (0.700, 0.933)  0.782 (0.212, 2.879)  1.692 (1.128, 2.539)  1.242 (0.334, 4.619)  0.854 (0.623, 1.171)  0.891 (0.657, 1.207)  1.052 (0.785, 1.411)  0.881 (0.659, 1.177)  1.399 (1.109, 1.764)  2.277 (1.856, 2.794)  1.530 (1.097, 2.134)  1.128 (0.935, 1.362)  1.170 (0.977, 1.401) | **p<0.001***  **p<0.05**  **p<0.05**  **p<0.01**  p>0.05  **p<0.05**  **p<0.01**  p>0.05  **p<0.05**  **p<0.05**  p>0.05  p>0.05  p>0.05  p>0.05  p>0.05  p>0.05  **p<0.001***  **p<0.01**  **p<0.001***  **p<0.05**  p>0.05  p>0.05  p>0.05 |
| Using green spaces | Importance of using green spaces       Age (ref. Young adults)            Middle-aged            Young-old            Old-old       Gender (ref. Male)            Female            Other       Ethnicity (ref. White)            Non-White            Prefer not to say       Deprivation (ref. Quintile 1 - Most deprived)            Quintile 2            Quintile 3            Quintile 4            Quintile 5 – Least deprived       Education (ref. Lower)            Middle            Higher            Other      Physical health (ref. Poor/fair)            Good            Very good/excellent | 5.297 (3.226, 8.699)  1.397 (0.631, 3.091)  2.063 (0.956, 4.453)  1.834 (0.608, 5.530)  1.059 (0.668, 1.678)  0.114 (0.019, 0.661)  0.190 (0.098, 0.371)  47232132.338 (0.000, -)  1.301 (0.600, 2.824)  1.257 (0.572, 2.763)  1.423 (0.667, 3.037)  1.580 (0.740, 3.372)  0.985 (0.540, 1.798)  2.008 (1.130, 3.568)  1.755 (0.577, 5.335)  2.133 (1.338, 3.401)  4.771 (2.767, 8.228) | **p<0.001***  p>0.05  p>0.05  p<0.05  p>0.05  p>0.05  p>0.05  **p<0.05**  **p<0.001***  **p<0.001***  p>0.05  p>0.05  p>0.05  p>0.05  p>0.05  p>0.05  **p<0.05**  p>0.05  **p<0.05**  p>0.05  **p<0.001***  **p<0.01***  **p<0.001*** |
| Wearing hearing aids | Importance of wearing hearing aids       Age (ref. Young adults)            Middle-aged            Young-old            Old-old       Gender (ref. Male)            Female            Other       Ethnicity (ref. White)            Non-White            Prefer not to say       Deprivation (ref. Quintile 1 - Most deprived)            Quintile 2            Quintile 3            Quintile 4            Quintile 5 – Least deprived       Education (ref. Lower)            Middle            Higher            Other      Physical health (ref. Poor/fair)            Good            Very good/excellent | 3.745 (2.883, 4.864)  1.948 (0.760, 4.994)  8.537 (3.442, 21.177)  33.181 (12.906, 85.309)  2.008 (1.686, 2.392)  1.997 (0.334, 11.921)  0.523 (0.275, 0.994)  3.260 (0.766, 13.864)  0.898 (0.595, 1.355)  0.782 (0.527, 1.162)  0.831 (0.568, 1.217)  0.826 (0.567, 1.205)  1.175 (0.887, 1.557)  0.999 (0.778, 1.282)  0.971 (0.648, 1.455)  0.813 (0.647, 1.023)  0.669 (0.535, 0.835) | **p<0.001***  **p<0.001***  p>0.05  **p<0.001***  **p<0.001***  **p<0.001***  **p<0.001***  p>0.05  **p<0.05**  **p<0.05**  p>0.05  p>0.05  p>0.05  p>0.05  p>0.05  p>0.05  p>0.05  p>0.05  p>0.05  p>0.05  **p<0.01***  p>0.05  **p<0.001*** |
| Taking illegal substances | Importance of not taking illegal substances       Age (ref. Young adults)            Middle-aged            Young-old            Old-old       Gender (ref. Male)            Female            Other       Ethnicity (ref. White)            Non-White            Prefer not to say       Deprivation (ref. Quintile 1 - Most deprived)            Quintile 2            Quintile 3            Quintile 4            Quintile 5 – Least deprived       Education (ref. Lower)            Middle            Higher            Other      Physical health (ref. Poor/fair)            Good            Very good/excellent | 0.197 (0.098, 0.396)  0.699 (0.337, 1.450)  0.353 (0.171, 0.730)  0.397 (0.128, 1.231)  1.617 (1.049, 2.492)  0.000 (0.000, -)  1.696 (0.741, 3.883)  0.000 (0.000, -)  0.672 (0.330, 1.369)  0.387 (0.178, 0.842)  0.389 (0.191, 0.796)  0.441 (0.221, 0.880)  1.286 (0.578, 2.858)  1.431 (0.699, 2.933)  1.167 (0.350, 3.888)  0.831 (0.485, 1.422)  0.637 (0.374, 1.087) | **p<0.001***  **p<0.01**  p>0.05  **p<0.01**  p>0.05  p>0.05  **p<0.05**  p>0.05  p>0.05  p>0.05  p>0.05  **p<0.05**  p>0.05  **p<0.05**  **p<0.05**  **p<0.05**  p>0.05  p>0.05  p>0.05  p>0.05  p>0.05  p>0.05  p>0.05 |
| Managing mental wellbeing | Importance of managing mental wellbeing       Age (ref. Young adults)            Middle-aged            Young-old            Old-old       Gender (ref. Male)            Female            Other       Ethnicity (ref. White)            Non-White            Prefer not to say       Deprivation (ref. Quintile 1 - Most deprived)            Quintile 2            Quintile 3            Quintile 4            Quintile 5 – Least deprived       Education (ref. Lower)            Middle            Higher            Other      Physical health (ref. Poor/fair)            Good            Very good/excellent | 6.654 (3.602, 12.293)  0.547 (0.209, 1.432)  0.507 (0.199, 1.292)  0.487 (0.169, 1.403)  0.579 (0.438, 0.767)  0.276 (0.033, 2.306)  1.231 (0.492, 3.080)  0.282 (0.058, 1.375)  0.542 (0.253, 1.159)  0.648 (0.305, 1.378)  0.559 (0.270, 1.157)  0.570 (0.277, 1.172)  1.229 (0.793, 1.904)  1.414 (0.962, 2.076)  1.538 (0.748, 3.162)  0.992 (0.686, 1.434)  1.329 (0.919, 1.923) | **p<0.001***  p>0.05  p>0.05  p>0.05  p>0.05  **p<0.001***  **p<0.001***  p>0.05  p>0.05  p>0.05  p>0.05  p>0.05  p>0.05  p>0.05  p>0.05  p>0.05  p>0.05  p>0.05  p>0.05  p>0.05  p>0.05  p>0.05  p>0.05 |
| Managing weight | Importance of managing weight       Age (ref. Young adults)            Middle-aged            Young-old            Old-old       Gender (ref. Male)            Female            Other       Ethnicity (ref. White)            Non-White            Prefer not to say       Deprivation (ref. Quintile 1 - Most deprived)            Quintile 2            Quintile 3            Quintile 4            Quintile 5 – Least deprived       Education (ref. Lower)            Middle            Higher            Other      Physical health (ref. Poor/fair)            Good            Very good/excellent | 4.587 (3.278, 6.420)  1.363 (0.832, 2.233)  1.937 (1.205, 3.115)  1.382 (0.743, 2.571)  0.908 (0.709, 1.164)  1.753 (0.188, 16.384)  0.606 (0.347, 1.055)  150931771.44 (0.000, -)  0.688 (0.403, 1.173)  0.931 (0.542, 1.602)  0.834 (0.498, 1.396)  0.826 (0.496, 1.375)  1.268 (0.868, 1.853)  1.300 (0.936, 1.806)  1.098 (0.629, 1.916)  1.358 (1.021, 1.806)  2.179 (1.625, 2.923) | **p<0.001***  **p<0.01**  p>0.05  **p<0.01**  p>0.05  p>0.05  p>0.05  p>0.05  p>0.05  p>0.05  p>0.05  p>0.05  p>0.05  p>0.05  p>0.05  p>0.05  p>0.05  p>0.05  p>0.05  p>0.05  **p<0.001***  **p<0.05**  **p<0.001*** |
| Mindful Activities | Importance of mindful activities       Age (ref. Young adults)            Middle-aged            Young-old            Old-old       Gender (ref. Male)            Female            Other       Ethnicity (ref. White)            Non-White            Prefer not to say       Deprivation (ref. Quintile 1 - Most deprived)            Quintile 2            Quintile 3            Quintile 4            Quintile 5 – Least deprived       Education (ref. Lower)            Middle            Higher            Other      Physical health (ref. Poor/fair)            Good            Very good/excellent | 7.811 (6.671, 9.145)  1.183 (0.835, 1.676)  1.648 (1.179, 2.304)  2.187 (1.403, 3.408)  0.546 (0.468, 0.638)  1.152 (0.287, 4.624)  1.820 (1.182, 2.804)  1.288 (0.317, 5.225)  0.758 (0.542, 1.061)  0.914 (0.660, 1.266)  0.958 (0.700, 1.311)  0.868 (0.637, 1.184)  1.196 (0.937, 1.526)  1.616 (1.305, 2.002)  1.539 (1.079, 2.196)  1.010 (0.826, 1.234)  0.954 (0.788, 1.157) | **p<0.001***  **p<0.001***  p>0.05  **p<0.01**  **p<0.001***  **p<0.001***  **p<0.001***  p>0.05  **p<0.05**  **p<0.01**  p>0.05  p>0.05  p>0.05  p>0.05  p>0.05  p>0.05  **p<0.001***  p>0.05  **p<0.001***  **p<0.05**  p>0.05  p>0.05  p>0.05 |
| Being near water | Importance of being near water       Age (ref. Young adults)            Middle-aged            Young-old            Old-old       Gender (ref. Male)            Female            Other       Ethnicity (ref. White)            Non-White            Prefer not to say       Deprivation (ref. Quintile 1 - Most deprived)            Quintile 2            Quintile 3            Quintile 4            Quintile 5 – Least deprived       Education (ref. Lower)            Middle            Higher            Other      Physical health (ref. Poor/fair)            Good            Very good/excellent | 3.607 (3.022, 4.306)  1.173 (0.783, 1.757)  1.585 (1.073, 2.340)  1.124 (0.680, 1.857)  1.215 (1.000, 1.476)  0.570 (0.129, 2.511)  0.445 (0.290, 0.684)  1.915 (0.231, 15.912)  1.325 (0.905, 1.938)  1.480 (1.016, 2.157)  1.560 (1.088, 2.237)  1.205 (0.849, 1.709)  1.321 (0.985, 1.772)  1.321 (1.026, 1.702)  1.462 (0.934, 2.288)  1.670 (1.333, 2.092)  2.500 (1.996, 3.131) | **p<0.001***  **p<0.01**  p>0.05  **p<0.05**  p>0.05  p>0.05  p>0.05  p>0.05  **p<0.001***  **p<0.001***  p>0.05  p>0.05  p>0.05  **p<0.05**  **p<0.05**  p>0.05  p>0.05  p>0.05  **p<0.05**  p>0.05  **p<0.001***  **p<0.001***  **p<0.001*** |
| Eating nuts, seeds and legumes | Importance of eating nuts, seeds and legumes       Age (ref. Young adults)            Middle-aged            Young-old            Old-old       Gender (ref. Male)            Female            Other       Ethnicity (ref. White)            Non-White            Prefer not to say       Deprivation (ref. Quintile 1 - Most deprived)            Quintile 2            Quintile 3            Quintile 4            Quintile 5 – Least deprived       Education (ref. Lower)            Middle            Higher            Other      Physical health (ref. Poor/fair)            Good            Very good/excellent | 7.205 (4.991, 10.402)  2.837 (1.534, 5.246)  4.748 (2.637, 8.552)  15.027 (4.142, 54.525)  0.854 (0.591, 1.235)  382898971.82 (0.000, -)  1.251 (0.438, 3.578)  69185187.767 (0.000, -)  0.917 (0.486, 1.729)  1.317 (0.674, 2.572)  1.594 (0.844, 3.012)  1.187 (0.645, 2.184)  1.115 (0.703, 1.767)  2.946 (1.869, 4.642)  2.617 (1.057, 6.482)  1.652 (1.105, 2.468)  2.985 (1.939, 4.595) | **p<0.001***  **p<0.001***  **p<0.001***  **p<0.001***  **p<0.001***  p>0.05  p>0.05  p>0.05  p>0.05  p>0.05  p>0.05  p>0.05  p>0.05  p>0.05  p>0.05  p>0.05  **p<0.001***  p>0.05  **p<0.001***  **p<0.05**  **p<0.001***  **p<0.05**  **p<0.001*** |
| Eating processed foods | Importance of eating less processed foods       Age (ref. Young adults)            Middle-aged            Young-old            Old-old       Gender (ref. Male)            Female            Other       Ethnicity (ref. White)            Non-White            Prefer not to say       Deprivation (ref. Quintile 1 - Most deprived)            Quintile 2            Quintile 3            Quintile 4            Quintile 5 – Least deprived       Education (ref. Lower)            Middle            Higher            Other      Physical health (ref. Poor/fair)            Good            Very good/excellent | 0.328 (0.103, 1.044)  0.148 (0.036, 0.616)  0.102 (0.025, 0.416)  0.065 (0.015, 0.281)  1.841 (1.380, 2.456)  100553267.59 (0.000, -)  0.387 (0.220, 0.678)  0.541 (0.066, 4.422)  0.957 (0.532, 1.721)  0.942 (0.536, 1.655)  0.995 (0.575, 1.722)  1.034 (0.600, 1.780)  1.027 (0.695, 1.518)  1.198 (0.850, 1.689)  0.837 (0.496, 1.413)  1.075 (0.768, 1.505)  1.003 (0.728, 1.382) | p>0.05  **p<0.001***  **p<0.01**  **p<0.01***  **p<0.001***  **p<0.001***  **p<0.001***  p>0.05  **p<0.01**  **p<0.001***  p>0.05  p>0.05  p>0.05  p>0.05  p>0.05  p>0.05  p>0.05  p>0.05  p>0.05  p>0.05  p>0.05  p>0.05  p>0.05 |
| Protecting against hearing loss | Importance of protecting against hearing loss       Age (ref. Young adults)            Middle-aged            Young-old            Old-old       Gender (ref. Male)            Female            Other       Ethnicity (ref. White)            Non-White            Prefer not to say       Deprivation (ref. Quintile 1 - Most deprived)            Quintile 2            Quintile 3            Quintile 4            Quintile 5 – Least deprived       Education (ref. Lower)            Middle            Higher            Other      Physical health (ref. Poor/fair)            Good            Very good/excellent | 3.674 (2.979, 4.531)  0.932 (0.654, 1.327)  1.198 (0.855, 1.678)  1.399 (0.908, 2.156)  1.542 (1.333, 1.782)  1.610 (0.435, 5.961)  1.002 (0.671, 1.496)  2.187 (0.585, 8.169)  0.956 (0.689, 1.327)  1.069 (0.782, 1.462)  1.198 (0.886, 1.620)  1.006 (0.746, 1.358)  1.070 (0.848, 1.349)  1.000 (0.816, 1.227)  0.960 (0.684, 1.347)  1.056 (0.870, 1.282)  1.163 (0.966, 1.400) | **p<0.001***  **p<0.01**  p>0.05  p>0.05  p>0.05  **p<0.001***  **p<0.001***  p>0.05  p>0.05  p>0.05  p>0.05  p>0.05  p>0.05  p>0.05  p>0.05  p>0.05  p>0.05  p>0.05  p>0.05  p>0.05  p>0.05  p>0.05  p>0.05 |
| Purpose in life | Importance of having a purpose in life       Age (ref. Young adults)            Middle-aged            Young-old            Old-old       Gender (ref. Male)            Female            Other       Ethnicity (ref. White)            Non-White            Prefer not to say       Deprivation (ref. Quintile 1 - Most deprived)            Quintile 2            Quintile 3            Quintile 4            Quintile 5 – Least deprived       Education (ref. Lower)            Middle            Higher            Other      Physical health (ref. Poor/fair)            Good            Very good/excellent | 8.237 (5.003, 13.560)  0.884 (0.386, 2.027)  1.081 (0.485, 2.411)  1.355 (0.452, 4.060)  0.671 (0.466, 0.967)  112318597.23 (0.000, -)  0.824 (0.322, 2.105)  0.378 (0.046, 3.083)  0.957 (0.464, 1.977)  1.337 (0.627, 2.850)  1.243 (0.616, 2.505)  1.247 (0.627, 2.477)  1.011 (0.571, 1.789)  1.061 (0.640, 1.759)  2.486 (0.726, 8.509)  2.274 (1.514, 3.414)  4.245 (2.724, 6.615) | **p<0.001***  p>0.05  p>0.05  p>0.05  p>0.05  p>0.05  **p<0.05**  p>0.05  p>0.05  p>0.05  p>0.05  p>0.05  p>0.05  p>0.05  p>0.05  p>0.05  p>0.05  p>0.05  p>0.05  p>0.05  **p<0.001***  **p<0.001***  **p<0.001*** |
| Eating red meat | Importance of eating less red meat       Age (ref. Young adults)            Middle-aged            Young-old            Old-old       Gender (ref. Male)            Female            Other       Ethnicity (ref. White)            Non-White            Prefer not to say       Deprivation (ref. Quintile 1 - Most deprived)            Quintile 2            Quintile 3            Quintile 4            Quintile 5 – Least deprived       Education (ref. Lower)            Middle            Higher            Other      Physical health (ref. Poor/fair)            Good            Very good/excellent | 0.295 (0.226, 0.385)  0.883 (0.587, 1.327)  1.016 (0.686, 1.505)  1.192 (0.691, 2.057)  1.651 (1.355, 2.013)  1.013 (0.204, 5.029)  0.789 (0.504, 1.236)  0.764 (0.154, 3.789)  0.969 (0.664, 1.415)  1.078 (0.746, 1.556)  1.522 (1.060, 2.184)  1.421 (0.996, 2.026)  0.696 (0.504, 0.961)  0.536 (0.403, 0.712)  0.947 (0.588, 1.525)  0.976 (0.762, 1.250)  0.847 (0.670, 1.070) | **p<0.001***  p>0.05  p>0.05  p>0.05  p>0.05  **p<0.001***  **p<0.001***  p>0.05  p>0.05  p>0.05  p>0.05  **p<0.001***  p>0.05  p>0.05  **p<0.05**  p>0.05  **p<0.001***  **p<0.05**  **p<0.001***  p>0.05  p>0.05  p>0.05  p>0.05 |
| Smoking or vaping | Importance of not smoking or vaping       Age (ref. Young adults)            Middle-aged            Young-old            Old-old       Gender (ref. Male)            Female            Other       Ethnicity (ref. White)            Non-White            Prefer not to say       Deprivation (ref. Quintile 1 - Most deprived)            Quintile 2            Quintile 3            Quintile 4            Quintile 5 – Least deprived       Education (ref. Lower)            Middle            Higher            Other      Physical health (ref. Poor/fair)            Good            Very good/excellent | 0.183 (0.107, 0.312)  0.388 (0.246, 0.613)  0.136 (0.085, 0.215)  0.108 (0.045, 0.261)  1.092 (0.790, 1.508)  0.000 (0.000, -)  1.046 (0.524, 2.086)  2.131 (0.263, 17.238)  0.544 (0.332, 0.892)  0.411 (0.246, 0.686)  0.359 (0.221, 0.583)  0.361 (0.224, 0.581)  0.687 (0.443, 1.065)  0.498 (0.336, 0.736)  0.626 (0.301, 1.302)  0.535 (0.373, 0.766)  0.429 (0.300, 0.613) | **p<0.001***  **p<0.001***  **p<0.001***  **p<0.001***  **p<0.001***  p>0.05  p>0.05  p>0.05  p>0.05  p>0.05  p>0.05  **p<0.001***  **p<0.05**  **p<0.001***  **p<0.001***  **p<0.001***  **p<0.01**  p>0.05  **p<0.001***  p>0.05  **p<0.001***  **p<0.001***  **p<0.001*** |
| Socialising | Importance of socialising       Age (ref. Young adults)            Middle-aged            Young-old            Old-old       Gender (ref. Male)            Female            Other       Ethnicity (ref. White)            Non-White            Prefer not to say       Deprivation (ref. Quintile 1 - Most deprived)            Quintile 2            Quintile 3            Quintile 4            Quintile 5 – Least deprived       Education (ref. Lower)            Middle            Higher            Other      Physical health (ref. Poor/fair)            Good            Very good/excellent | 4.666 (2.966, 7.340)  0.360 (0.104, 1.248)  0.341 (0.101, 1.153)  0.482 (0.121, 1.921)  0.536 (0.380, 0.758)  0.170 (0.020, 1.467)  0.453 (0.218, 0.942)  74625271.646 (0.000, -)  1.145 (0.578, 2.267)  1.525 (0.765, 3.039)  1.708 (0.879, 3.319)  1.494 (0.784, 2.849)  1.063 (0.611, 1.849)  0.995 (0.611, 1.621)  2.703 (0.795, 9.193)  1.580 (1.058, 2.359)  2.922 (1.897, 4.499) | **p<0.001***  p>0.05  p>0.05  p>0.05  p>0.05  **p<0.001***  **p<0.001***  p>0.05  p>0.05  **p<0.05**  p>0.05  p>0.05  p>0.05  p>0.05  p>0.05  p>0.05  p>0.05  p>0.05  p>0.05  p>0.05  **p<0.001***  **p<0.05**  **p<0.001*** |
| Strength training | Importance of strength training       Age (ref. Young adults)            Middle-aged            Young-old            Old-old       Gender (ref. Male)            Female            Other       Ethnicity (ref. White)            Non-White            Prefer not to say       Deprivation (ref. Quintile 1 - Most deprived)            Quintile 2            Quintile 3            Quintile 4            Quintile 5 – Least deprived       Education (ref. Lower)            Middle            Higher            Other      Physical health (ref. Poor/fair)            Good            Very good/excellent | 7.711 (6.535, 9.099)  0.805 (0.561, 1.156)  0.877 (0.620, 1.241)  0.653 (0.413, 1.035)  1.259 (1.077, 1.473)  3.390 (0.638, 18.013)  1.252 (0.828, 1.893)  1.508 (0.379, 6.003)  1.296 (0.923, 1.820)  1.326 (0.955, 1.842)  1.469 (1.070, 2.018)  1.583 (1.156, 2.166)  1.274 (0.997, 1.628)  1.404 (1.132, 1.741)  1.546 (1.081, 2.211)  1.519 (1.242, 1.858)  2.417 (1.992, 2.933) | **p<0.001***  p>0.05  p>0.05  p>0.05  p>0.05  **p<0.01**  **p<0.01**  p>0.05  p>0.05  p>0.05  p>0.05  **p<0.05**  p>0.05  p>0.05  **p<0.05**  **p<0.01**  **p<0.05**  p>0.05  **p<0.01**  **p<0.05**  **p<0.001***  **p<0.001***  **p<0.001*** |
| Taking vitamins and supplements | Importance of taking vitamins and supplements       Age (ref. Young adults)            Middle-aged            Young-old            Old-old       Gender (ref. Male)            Female            Other       Ethnicity (ref. White)            Non-White            Prefer not to say       Deprivation (ref. Quintile 1 - Most deprived)            Quintile 2            Quintile 3            Quintile 4            Quintile 5 – Least deprived       Education (ref. Lower)            Middle            Higher            Other      Physical health (ref. Poor/fair)            Good            Very good/excellent | 6.807 (5.733, 8.083)  1.461 (0.974, 2.192)  1.731 (1.172, 2.555)  1.757 (1.068, 2.890)  0.715 (0.606, 0.843)  1.948 (0.371, 10.217)  1.017 (0.622, 1.663)  0.996 (0.183, 5.420)  0.718 (0.482, 1.070)  0.650 (0.442, 0.957)  0.780 (0.536, 1.135)  0.752 (0.518, 1.091)  1.292 (0.988, 1.690)  1.661 (1.341, 2.099)  1.521 (1.012, 2.286)  1.140 (0.907, 1.432)  0.976 (0.787, 1.210) | **p<0.001***  **p<0.05**  p>0.05  **p<0.01**  **p<0.05**  **p<0.001***  **p<0.001***  p>0.05  p>0.05  p>0.05  p>0.05  p>0.05  p>0.05  **p<0.05**  p>0.05  p>0.05  **p<0.001***  p>0.05  **p<0.001***  **p<0.05**  p>0.05  p>0.05  p>0.05 |
| Volunteering and helping others | Importance of volunteering and helping others       Age (ref. Young adults)            Middle-aged            Young-old            Old-old       Gender (ref. Male)            Female            Other       Ethnicity (ref. White)            Non-White            Prefer not to say       Deprivation (ref. Quintile 1 - Most deprived)            Quintile 2            Quintile 3            Quintile 4            Quintile 5 – Least deprived       Education (ref. Lower)            Middle            Higher            Other      Physical health (ref. Poor/fair)            Good            Very good/excellent | 3.815 (3.227, 4.510)  1.238 (0.872, 1.758)  1.983 (1.414 2.783)  2.170 (1.366, 3.447)  0.620 (0.528, 0.727)  0.385 (0.102, 1.454)  0.980 (0.636, 1.508)  1.125 (0.274, 4.610)  1.054 (0.750, 1.480)  1.255 (0.900, 1.751)  1.228 (0.892, 1.689)  1.339 (0.976, 1.836)  0.961 (0.748, 1.233)  1.349 (1.079, 1.685)  1.368 (0.925, 2.023)  1.592 (1.300, 1.951)  1.851 (1.522, 2.251) | **p<0.001***  **p<0.001***  p>0.05  **p<0.001***  **p<0.01***  **p<0.001***  **p<0.001***  p>0.05  p>0.05  p>0.05  p>0.05  p>0.05  p>0.05  p>0.05  p>0.05  p>0.05  **p<0.001***  p>0.05  **p<0.01**  p>0.05  **p<0.001***  **p<0.001***  **p<0.001*** |

*Note.* Bolded comparisons are significant at p<0.05. * denotes comparisons remaining significant after Bonferroni correction at p<0.002.

# Supplementary Table 5: Future behaviours - By familiarity

| Dependent variable | Predictor and covariates | Odds Ratio (95% CI) | P-value |
| --- | --- | --- | --- |
| Drinking 2 litres of water | Familiarity with cognitive frailty       Age (ref. Young adults)            Middle-aged            Young-old            Old-old       Gender (ref. Male)            Female            Other       Ethnicity (ref. White)            Non-White            Prefer not to say      Deprivation (ref. Quintile 1 - Most deprived)            Quintile 2            Quintile 3            Quintile 4            Quintile 5 – Least deprived       Education (ref. Lower)            Middle            Higher            Other      Physical health (ref. Poor/fair)            Good            Very good/excellent | 1.197 (0.932, 1.537)  0.428 (0.166, 1.107)  0.255 (0.101, 0.642)  0.115 (0.043, 0.302)    0.408 (0.329, 0.507)  0.213 (0.040, 1.128)    1.386 (0.660, 2.912)  1.168 (0.144, 9.495)    1.132 (0.680, 1.886)  1.145 (0.702, 1.868)  1.408 (0.872, 2.272)  1.239 (0.776, 1.978)    1.360 (0.936, 1.976)  1.022 (0.746, 1.399)  1.699 (0.931, 3.099)    1.267 (0.943, 1.703)  1.250 (0.942, 1.658) | p>0.05  **p<0.001***  p>0.05  **p<0.01**  **p<0.001***  **p<0.001***  **p<0.001***  p>0.05  p>0.05  p>0.05  p>0.05  p>0.05  p>0.05  p>0.05  p>0.05  p>0.05  p>0.05  p>0.05  p>0.05  p>0.05  p>0.05  p>0.05  p>0.05 |
| Drinking less or no alcohol | Familiarity with cognitive frailty       Age (ref. Young adults)            Middle-aged            Young-old            Old-old       Gender (ref. Male)            Female            Other       Ethnicity (ref. White)            Non-White            Prefer not to say       Deprivation (ref. Quintile 1 - Most deprived)            Quintile 2            Quintile 3            Quintile 4            Quintile 5 – Least deprived       Education (ref. Lower)            Middle            Higher            Other      Physical health (ref. Poor/fair)            Good            Very good/excellent | 1.261 (1.054, 1.508)  1.143 (0.757, 1.726)  0.895 (0.605, 1.323)  0.595 (0.370, 0.957)    0.442 (0.378, 0.517)  2.128 (0.264, 17.156)    1.478 (0.906, 2.411)  0.529 (0.145, 1.934)    0.856 (0.595, 1.231)  1.055 (0.739, 1.506)  1.060 (0.752, 1.493)  1.066 (0.760, 1.497)    1.022 (0.783, 1.335)  0.908 (0.719, 1.146)  1.143 (0.767, 1.703)    0.962 (0.778, 1.189)  1.212 (0.985, 1.491) | **p<0.05**  **p<0.01***  p>0.05  p>0.05  **p<0.05**  **p<0.001***  **p<0.001***  p>0.05  p>0.05  p>0.05  p>0.05  p>0.05  p>0.05  p>0.05  p>0.05  p>0.05  p>0.05  p>0.05  p>0.05  p>0.05  **p<0.05**  p>0.05  p>0.05 |
| Educational classes | Familiarity with cognitive frailty       Age (ref. Young adults)            Middle-aged            Young-old            Old-old       Gender (ref. Male)            Female            Other       Ethnicity (ref. White)            Non-White            Prefer not to say       Deprivation (ref. Quintile 1 - Most deprived)            Quintile 2            Quintile 3            Quintile 4            Quintile 5 – Least deprived       Education (ref. Lower)            Middle            Higher            Other      Physical health (ref. Poor/fair)            Good            Very good/excellent | 1.319 (1.116, 1.558)  1.137 (0.780, 1.657)  0.970 (0.677, 1.389)  1.017 (0.647, 1.599)    0.556 (0.479, 0.644)  3.130 (0.384, 25.504)    1.343 (0.864, 2.090)  0.349 (0.094, 1.302)    0.995 (0.715, 1.385)  1.234 (0.894, 1.703)  1.080 (0.793, 1.469)  1.001 (0.738, 1.357)    1.758 (1.403, 2.204)  2.567 (2.103, 3.132)  1.479 (1.064, 2.056)    1.400 (1.156, 1.696)  1.678 (1.394, 2.020) | **p<0.01***  p>0.05  p>0.05  p>0.05  p>0.05  **p<0.001***  **p<0.001***  p>0.05  p>0.05  p>0.05  p>0.05  p>0.05  p>0.05  p>0.05  p>0.05  p>0.05  **p<0.001***  **p<0.001***  **p<0.001***  **p<0.05**  **p<0.001***  **p<0.001***  **p<0.001*** |
| Limiting exposure to air pollution | Familiarity with cognitive frailty       Age (ref. Young adults)            Middle-aged            Young-old            Old-old       Gender (ref. Male)            Female            Other       Ethnicity (ref. White)            Non-White            Prefer not to say       Deprivation (ref. Quintile 1 - Most deprived)            Quintile 2            Quintile 3            Quintile 4            Quintile 5 – Least deprived       Education (ref. Lower)            Middle            Higher            Other      Physical health (ref. Poor/fair)            Good            Very good/excellent | 1.396 (1.158, 1.682)  1.293 (0.877, 1.905)  1.412 (0.975, 2.044)  0.938 (0.588, 1.495)  0.635 (0.536, 0.752)  1.085 (0.222, 5.292)  0.900 (0.575, 1.407)  1.091 (0.230, 5.180)  1.240 (0.867, 1.774)  1.733 (1.213, 2.476)  1.327 (0.950, 1.854)  1.358 (0.975, 1.890)  0.995 (0.750, 1.320)  0.937 (0.731, 1.200)  0.914 (0.609, 1.373)  0.994 (0.798, 1.237)  1.389 (1.117, 1.726) | **p<0.001***  **p<0.05**  p>0.05  p>0.05  p>0.05  **p<0.001***  **p<0.001***  p>0.05  p>0.05  p>0.05  p>0.05  **p<0.05**  p>0.05  **p<0.01**  p>0.05  p>0.05  p>0.05  p>0.05  p>0.05  p>0.05  **p<0.001***  p>0.05  **p<0.01** |
| Using green and blue spaces | Familiarity with cognitive frailty       Age (ref. Young adults)            Middle-aged            Young-old            Old-old       Gender (ref. Male)            Female            Other       Ethnicity (ref. White)            Non-White            Prefer not to say       Deprivation (ref. Quintile 1 - Most deprived)            Quintile 2            Quintile 3            Quintile 4            Quintile 5 – Least deprived       Education (ref. Lower)            Middle            Higher            Other      Physical health (ref. Poor/fair)            Good            Very good/excellent | 1.911 (1.349, 2.708)  1.333 (0.570, 3.117)  1.134 (0.509, 2.523)  0.626 (0.251, 1.561)  0.437 (0.313, 0.612)  0.151 (0.028, 0.806)  0.823 (0.349, 1.941)  0.433 (0.053, 3.532)  1.127 (0.577, 2.201)  1.915 (0.953, 3.850)  1.888 (0.972, 3.666)  1.368 (0.727, 2.576)  1.206 (0.673, 2.163)  0.978 (0.595, 1.608)  0.605 (0.293, 1.249)  1.700 (1.139, 2.536)  2.564 (1.696, 3.874) | **p<0.001***  p>0.05  p>0.05  p>0.05  p>0.05  **p<0.001***  **p<0.001***  **p<0.05**  p>0.05  p>0.05  p>0.05  p>0.05  p>0.05  p>0.05  p>0.05  p>0.05  p>0.05  p>0.05  p>0.05  p>0.05  **p<0.001***  **p<0.01**  **p<0.001*** |
| Healthy diet | Familiarity with cognitive frailty       Age (ref. Young adults)            Middle-aged            Young-old            Old-old       Gender (ref. Male)            Female            Other       Ethnicity (ref. White)            Non-White            Prefer not to say       Deprivation (ref. Quintile 1 - Most deprived)            Quintile 2            Quintile 3            Quintile 4            Quintile 5 – Least deprived       Education (ref. Lower)            Middle            Higher            Other      Physical health (ref. Poor/fair)            Good            Very good/excellent | 1.759 (1.163, 2.660)  1.020 (0.413, 2.522)  1.272 (0.530, 3.053)  1.596 (0.492, 5.176)  0.412 (0.277, 0.613)  0.111 (0.021, 0.604)  0.641 (0.268, 1.534)  61723494.356 (0.000, -)  1.042 (0.476, 2.282)  1.379 (0.621, 3.061)  1.380 (0.643, 2.962)  1.783 (0.822, 3.866)  0.999 (0.509, 1.958)  0.873 (0.482, 1.583)  38478627.751 (0.000, -)  1.975 (1.228, 3.174)  2.688 (1.668, 4.332) | **p<0.01**  p>0.05  p>0.05  p>0.05  p>0.05  **p<0.001***  **p<0.001***  **p<0.05**  p>0.05  p>0.05  p>0.05  p>0.05  p>0.05  p>0.05  p>0.05  p>0.05  p>0.05  p>0.05  p>0.05  p>0.05  **p<0.001***  **p<0.01**  **p<0.001*** |
| Managing mental wellbeing | Familiarity with cognitive frailty       Age (ref. Young adults)            Middle-aged            Young-old            Old-old       Gender (ref. Male)            Female            Other       Ethnicity (ref. White)            Non-White            Prefer not to say       Deprivation (ref. Quintile 1 - Most deprived)            Quintile 2            Quintile 3            Quintile 4            Quintile 5 – Least deprived       Education (ref. Lower)            Middle            Higher            Other      Physical health (ref. Poor/fair)            Good            Very good/excellent | 1.585 (1.202, 2.090)  0.835 (0.401, 1.740)  0.777 (0.383, 1.576)  0.677 (0.294, 1.558)  0.398 (0.309, 0.513)  83422200.213 (0.000, -)  0.738 (0.385, 1.415)  0.353 (0.073, 1.705)  0.973 (0.539, 1.758)  1.494 (0.823, 2.710)  1.061 (0.609, 1.847)  1.068 (0.617, 1.848)  1.291 (0.813, 2.053)  0.858 (0.583, 1.264)  1.150 (0.580, 2.281)  1.012 (0.714, 1.434)  1.171 (0.833, 1.647) | **p<0.01***  p>0.05  p>0.05  p>0.05  p>0.05  **p<0.001***  **p<0.001***  p>0.05  p>0.05  p>0.05  p>0.05  p>0.05  p>0.05  p>0.05  p>0.05  p>0.05  p>0.05  p>0.05  p>0.05  p>0.05  p>0.05  p>0.05  p>0.05 |
| Managing weight | Familiarity with cognitive frailty       Age (ref. Young adults)            Middle-aged            Young-old            Old-old       Gender (ref. Male)            Female            Other       Ethnicity (ref. White)            Non-White            Prefer not to say       Deprivation (ref. Quintile 1 - Most deprived)            Quintile 2            Quintile 3            Quintile 4            Quintile 5 – Least deprived       Education (ref. Lower)            Middle            Higher            Other      Physical health (ref. Poor/fair)            Good            Very good/excellent | 1.519 (1.139, 2.025)  1.319 (0.702, 2.475)  1.232 (0.678, 2.238)  0.877 (0.424, 1.814)  0.574 (0.440, 0.750)  0.653 (0.079, 5.423)  0.669 (0.357, 1.254)  0.767 (0.095, 6.161)  1.029 (0.584, 1.813)  1.609 (0.901, 2.876)  1.252 (0.729, 2.150)  1.259 (0.738, 2.149)  1.248 (0.775, 2.011)  0.839 (0.563, 1.251)  0.902 (0.468, 1.737)  1.442 (1.044, 1.991)  2.306 (1.653, 3.216) | **p<0.01**  p>0.05  p>0.05  p>0.05  p>0.05  **p<0.001***  **p<0.001***  p>0.05  p>0.05  p>0.05  p>0.05  p>0.05  p>0.05  p>0.05  p>0.05  p>0.05  p>0.05  p>0.05  p>0.05  p>0.05  **p<0.001***  **p<0.05**  **p<0.001*** |
| Mindful activities | Familiarity with cognitive frailty       Age (ref. Young adults)            Middle-aged            Young-old            Old-old       Gender (ref. Male)            Female            Other       Ethnicity (ref. White)            Non-White            Prefer not to say       Deprivation (ref. Quintile 1 - Most deprived)            Quintile 2            Quintile 3            Quintile 4            Quintile 5 – Least deprived       Education (ref. Lower)            Middle            Higher            Other      Physical health (ref. Poor/fair)            Good            Very good/excellent | 1.268 (1.078, 1.490)  0.888 (0.603, 1.307)  0.584 (0.404, 0.845)  0.392 (0.250, 0.614)  0.463 (0.402, 0.534)  0.822 (0.201, 3.355)  2.827 (1.701, 4.697)  0.619 (0.170, 2.251)  0.934 (0.676, 1.290)  1.175 (0.859, 1.607)  1.097 (0.812, 1.483)  1.054 (0.783, 1.420)  1.417 (1.131, 1.776)  1.626 (1.335, 1.981)  1.485 (1.064, 2.072)  0.952 (0.785, 1.155)  0.874 (0.726, 1.051) | **p<0.01**  **p<0.001***  p>0.05  **p<0.01**  **p<0.001***  **p<0.001***  **p<0.001***  p>0.05  **p<0.001***  **p<0.001***  p>0.05  p>0.05  p>0.05  p>0.05  p>0.05  p>0.05  **p<0.001***  **p<0.01**  **p<0.001***  **p<0.05**  p>0.05  p>0.05  p>0.05 |
| Physical activity | Familiarity with cognitive frailty       Age (ref. Young adults)            Middle-aged            Young-old            Old-old       Gender (ref. Male)            Female            Other       Ethnicity (ref. White)            Non-White            Prefer not to say       Deprivation (ref. Quintile 1 - Most deprived)            Quintile 2            Quintile 3            Quintile 4            Quintile 5 – Least deprived       Education (ref. Lower)            Middle            Higher            Other      Physical health (ref. Poor/fair)            Good            Very good/excellent | 1.454 (1.068, 1.979)  1.202 (0.621, 2.326)  1.321 (0.701, 2.490)  1.099 (0.499, 2.420)  0.708 (0.529, 0.947)  0.791 (0.085, 7.408)  1.094 (0.493, 2.429)  133439798.13 (0.000, -)  1.704 (1.025, 2.834)  2.933 (1.708, 5.035)  2.470 (1.510, 4.043)  2.154 (1.339, 3.467)  1.389 (0.901, 2.143)  1.490 (1.021, 2.174)  1.186 (0.625, 2.249)  2.566 (1.864, 3.533)  4.850 (3.418, 6.883) | **p<0.05**  p>0.05  p>0.05  p>0.05  p>0.05  p>0.05  **p<0.05**  p>0.05  p>0.05  p>0.05  p>0.05  **p<0.001***  **p<0.05**  **p<0.001***  **p<0.001***  **p<0.01**  p>0.05  p>0.05  **p<0.05**  p>0.05  **p<0.001***  **p<0.001***  **p<0.001*** |
| Protecting against hearing loss | Familiarity with cognitive frailty       Age (ref. Young adults)            Middle-aged            Young-old            Old-old       Gender (ref. Male)            Female            Other       Ethnicity (ref. White)            Non-White            Prefer not to say       Deprivation (ref. Quintile 1 - Most deprived)            Quintile 2            Quintile 3            Quintile 4            Quintile 5 – Least deprived       Education (ref. Lower)            Middle            Higher            Other      Physical health (ref. Poor/fair)            Good            Very good/excellent | 1.538 (1.277, 1.851)  1.307 (0.869, 1.967)  1.139 (0.773, 1.678)  0.767 (0.476, 1.238)    0.589 (0.497, 0.698)  1.020 (0.209, 4.987)  0.870 (0.555, 1.364)  1.183 (0.248, 5.655)  1.112 (0.778, 1.589)  2.003 (1.393, 2.880)  1.416 (1.010, 1.984)  1.371 (0.983, 1.911)  1.345 (1.018, 1.778)  1.122 (0.884, 1.424)  1.047 (0.704, 1.559)  1.161 (0.937, 1.440)  1.762 (1.422, 2.185) | **p<0.001***  **p<0.05**  p>0.05  p>0.05  p>0.05  **p<0.001***  **p<0.001***  p>0.05  p>0.05  p>0.05  p>0.05  **p<0.001***  p>0.05  **p<0.001***  **p<0.05**  p>0.05  p>0.05  **p<0.05**  p>0.05  p>0.05  **p<0.001***  p>0.05  **p<0.001*** |
| Purpose in life | Familiarity with cognitive frailty       Age (ref. Young adults)            Middle-aged            Young-old            Old-old       Gender (ref. Male)            Female            Other       Ethnicity (ref. White)            Non-White            Prefer not to say       Deprivation (ref. Quintile 1 - Most deprived)            Quintile 2            Quintile 3            Quintile 4            Quintile 5 – Least deprived       Education (ref. Lower)            Middle            Higher            Other      Physical health (ref. Poor/fair)            Good            Very good/excellent | 1.625 (1.237, 2.134)  1.460 (0.844, 2.525)  1.568 (0.933, 2.634)  2.202 (1.047, 4.631)  0.491 (0.381, 0.633)  0.127 (0.033, 0.481)  0.871 (0.465, 1.631)  0.866 (0.108, 6.933)  0.941 (0.564, 1.572)  2.058 (1.188, 3.566)  1.533 (0.926, 2.538)  1.384 (0.846, 2.264)  1.331 (0.830, 2.135)  0.763 (0.516, 1.127)  1.057 (0.532, 2.102)  1.114 (0.805, 1.541)  1.725 (1.242, 2.397) | **p<0.001***  p>0.05  p>0.05  p>0.05  **p<0.05**  **p<0.001***  **p<0.001***  **p<0.01**  p>0.05  p>0.05  p>0.05  **p<0.01**  p>0.05  **p<0.05**  p>0.05  p>0.05  **p<0.05**  p>0.05  p>0.05  p>0.05  **p<0.01***  p>0.05  **p<0.01*** |
| Not smoking or vaping | Familiarity with cognitive frailty       Age (ref. Young adults)            Middle-aged            Young-old            Old-old       Gender (ref. Male)            Female            Other       Ethnicity (ref. White)            Non-White            Prefer not to say       Deprivation (ref. Quintile 1 - Most deprived)            Quintile 2            Quintile 3            Quintile 4            Quintile 5 – Least deprived       Education (ref. Lower)            Middle            Higher            Other      Physical health (ref. Poor/fair)            Good            Very good/excellent | 1.386 (1.115, 1.722)  1.017 (0.639, 1.619)  1.101 (0.705, 1.720)  0.997 (0.562, 1.769)  0.796 (0.651, 0.974)  273720467.51 (0.000, -)  0.745 (0.455, 1.218)  0.386 (0.098, 1.515)  1.012 (0.670, 1.529)  1.639 (1.079, 2.490)  1.432 (0.965, 2.125)  1.338 (0.908, 1.970)  1.420 (1.010, 1.997)  0.943 (0.711, 1.251)  1.040 (0.645, 1.678)  1.282 (0.995, 1.652)  1.518 (1.185, 1.944) | **p<0.01**  p>0.05  p>0.05  p>0.05  p>0.05  p>0.05  **p<0.05**  p>0.05  p>0.05  p>0.05  p>0.05  **p<0.05**  p>0.05  **p<0.05**  p>0.05  p>0.05  **p<0.05**  **p<0.05**  p>0.05  p>0.05  **p<0.01**  p>0.05  **p<0.001*** |
| Socialising | Familiarity with cognitive frailty       Age (ref. Young adults)            Middle-aged            Young-old            Old-old       Gender (ref. Male)            Female            Other       Ethnicity (ref. White)            Non-White            Prefer not to say       Deprivation (ref. Quintile 1 - Most deprived)            Quintile 2            Quintile 3            Quintile 4            Quintile 5 – Least deprived       Education (ref. Lower)            Middle            Higher            Other      Physical health (ref. Poor/fair)            Good            Very good/excellent | 1.624 (1.254, 2.104)  1.226 (0.704, 2.135)  1.533 (0.899, 2.614)  1.635 (0.814, 3.285)  0.413 (0.325, 0.526)  0.184 (0.044, 0.768)  1.048 (0.546, 2.010)  0.280 (0.070, 1.120)  0.882 (0.519, 1.497)  1.254 (0.734, 2.143)  1.163 (0.697, 1.940)  1.071 (0.648, 1.771)  1.151 (0.754, 1.758)  0.856 (0.596, 1.231)  1.994 (0.910, 4.370)  1.473 (1.098, 1.976)  2.480 (1.830, 3.359) | **p<0.001***  p>0.05  p>0.05  p>0.05  p>0.05  **p<0.001***  **p<0.001***  **p<0.05**  p>0.05  p>0.05  p>0.05  p>0.05  p>0.05  p>0.05  p>0.05  p>0.05  **p<0.05**  p>0.05  p>0.05  p>0.05  **p<0.001***  **p<0.05**  **p<0.001*** |
| Taking vitamins and supplements | Familiarity with cognitive frailty       Age (ref. Young adults)            Middle-aged            Young-old            Old-old       Gender (ref. Male)            Female            Other       Ethnicity (ref. White)            Non-White            Prefer not to say       Deprivation (ref. Quintile 1 - Most deprived)            Quintile 2            Quintile 3            Quintile 4            Quintile 5 – Least deprived       Education (ref. Lower)            Middle            Higher            Other      Physical health (ref. Poor/fair)            Good            Very good/excellent | 1.098 (0.906, 1.331)  0.684 (0.402, 1.162)  0.466 (0.280, 0.775)  0.239 (0.135, 0.424)  0.487 (0.414, 0.574)  0.331 (0.079, 1.383)  1.362 (0.799, 2.321)  1.026 (0.212, 4.969)  0.829 (0.551, 1.248)  0.760 (0.513, 1.125)  0.860 (0.587, 1.260)  0.930 (0.636, 1.358)  1.230 (0.930, 1.626)  1.028 (0.809, 1.307)  1.086 (0.727, 1.622)  1.048 (0.830, 1.324)  0.866 (0.694, 1.080) | p>0.05  **p<0.001***  p>0.05  **p<0.01**  **p<0.001***  **p<0.001***  **p<0.001***  p>0.05  p>0.05  p>0.05  p>0.05  p>0.05  p>0.05  p>0.05  p>0.05  p>0.05  p>0.05  p>0.05  p>0.05  p>0.05  p>0.05  p>0.05  p>0.05 |
| Volunteering and helping others | Familiarity with cognitive frailty       Age (ref. Young adults)            Middle-aged            Young-old            Old-old       Gender (ref. Male)            Female            Other       Ethnicity (ref. White)            Non-White            Prefer not to say       Deprivation (ref. Quintile 1 - Most deprived)            Quintile 2            Quintile 3            Quintile 4            Quintile 5 – Least deprived       Education (ref. Lower)            Middle            Higher            Other      Physical health (ref. Poor/fair)            Good            Very good/excellent | 1.444 (1.189, 1.754)  1.438 (0.957, 2.162)  1.617 (1.096, 2.386)  1.868 (1.115, 3.132)  0.452 (0.380, 0.539)  0.846 (0.167, 4.276)  1.151 (0.696, 1.904)  1.135 (0.234, 5.505)  1.068 (0.729, 1.566)  1.561 (1.063, 2.292)  1.158 (0.809, 1.658)  1.051 (0.739, 1.497)  1.156 (0.878, 1.522)  1.440 (1.128, 1.838)  2.195 (1.348, 3.574)  1.643 (1.317, 2.051)  2.060 (1.659, 2.557) | **p<0.001***  p>0.05  p>0.05  **p<0.05**  **p<0.05**  **p<0.001***  **p<0.001***  p>0.05  p>0.05  p>0.05  p>0.05  **p<0.05**  p>0.05  **p<0.05**  p>0.05  p>0.05  **p<0.01***  p>0.05  **p<0.01**  **p<0.01**  **p<0.001***  **p<0.001***  **p<0.001*** |

*Note.* Bolded comparisons are significant at p<0.05. * denotes comparisons remaining significant after Bonferroni correction at p<0.002.

# Supplementary Table 6: Future behaviours - By preventability

| Dependent variable | Predictor and covariates | Odds Ratio (95% CI) | P-value |
| --- | --- | --- | --- |
| Drinking 2 litres of water | Preventability of cognitive frailty       Age (ref. Young adults)            Middle-aged            Young-old            Old-old       Gender (ref. Male)            Female            Other       Ethnicity (ref. White)            Non-White            Prefer not to say      Deprivation (ref. Quintile 1 - Most deprived)            Quintile 2            Quintile 3            Quintile 4            Quintile 5 – Least deprived       Education (ref. Lower)            Middle            Higher            Other      Physical health (ref. Poor/fair)            Good            Very good/excellent | 0.495 (0.365, 0.671)  0.426 (0.165, 1.101)  0.252 (0.100, 0.633)  0.116 (0.044, 0.306)  0.411 (0.331, 0.511)  0.213 (0.040, 1.122)  1.302 (0.620, 2.735)  1.023 (0.126, 8.301)  1.108 (0.663, 1.851)  1.125 (0.688, 1.839)  1.394 (0.861, 2.256)  1.227 (0.766, 1.964)  1.289 (0.885, 1.877)  0.942 (0.686, 1.296)  1.688 (0.923, 3.086)  1.206 (0.895, 1.624)  1.154 (0.866, 1.538) | **p<0.001***  **p<0.001***  p>0.05  **p<0.01**  **p<0.001***  **p<0.001***  **p<0.001***  p>0.05  p>0.05  p>0.05  p>0.05  p>0.05  p>0.05  p>0.05  p>0.05  p>0.05  **p<0.05**  p>0.05  p>0.05  p>0.05  p>0.05  p>0.05  p>0.05 |
| Drinking less or no alcohol | Preventability of cognitive frailty       Age (ref. Young adults)            Middle-aged            Young-old            Old-old       Gender (ref. Male)            Female            Other       Ethnicity (ref. White)            Non-White            Prefer not to say       Deprivation (ref. Quintile 1 - Most deprived)            Quintile 2            Quintile 3            Quintile 4            Quintile 5 – Least deprived       Education (ref. Lower)            Middle            Higher            Other      Physical health (ref. Poor/fair)            Good            Very good/excellent | 0.582 (0.459, 0.737)  1.140 (0.755, 1.724)  0.890 (0.602, 1.318)  0.602 (0.374, 0.970)  0.442 (0.378, 0.516)  2.062 (0.257, 16.542)  1.435 (0.880, 2.342)  0.482 (0.132, 1.766)  0.842 (0.585, 1.213)  1.034 (0.724, 1.477)  1.053 (0.746, 1.485)  1.059 (0.754, 1.488)  0.982 (0.751, 1.284)  0.853 (0.674, 1.079)  1.131 (0.758, 1.688)  0.931 (0.751, 1.152)  1.152 (0.934, 1.421) | **p<0.001***  **p<0.01**  p>0.05  p>0.05  **p<0.05**  **p<0.001***  **p<0.001***  p>0.05  p>0.05  p>0.05  p>0.05  p>0.05  p>0.05  p>0.05  p>0.05  p>0.05  p>0.05  p>0.05  p>0.05  p>0.05  **p<0.05**  p>0.05  p>0.05 |
| Educational classes | Preventability of cognitive frailty       Age (ref. Young adults)            Middle-aged            Young-old            Old-old       Gender (ref. Male)            Female            Other       Ethnicity (ref. White)            Non-White            Prefer not to say       Deprivation (ref. Quintile 1 - Most deprived)            Quintile 2            Quintile 3            Quintile 4            Quintile 5 – Least deprived       Education (ref. Lower)            Middle            Higher            Other      Physical health (ref. Poor/fair)            Good            Very good/excellent | 0.587 (0.470, 0.733)  1.134 (0.777, 1.654)  0.965 (0.673, 1.384)  1.030 (0.654, 1.621)  0.554 (0.478, 0.642)  2.989 (0.370, 24.159)  1.308 (0.841, 2.035)  0.320 (0.086, 1.193)  0.987 (0.709, 1.373)  1.213 (0.878, 1.675)  1.080 (0.793, 1.471)  0.999 (0.737, 1.354)  1.698 (1.353, 2.131)  2.425 (1.985, 2.964)  1.465 (1.053, 2.040)  1.367 (1.127, 1.657)  1.610 (1.335, 1.942) | **p<0.001***  p>0.05  p>0.05  p>0.05  p>0.05  **p<0.001***  **p<0.001***  p>0.05  p>0.05  p>0.05  p>0.05  p>0.05  p>0.05  p>0.05  p>0.05  p>0.05  **p<0.001***  **p<0.001***  **p<0.001***  **p<0.05**  **p<0.001***  **p<0.01***  **p<0.001*** |
| Limiting exposure to air pollution | Preventability of cognitive frailty       Age (ref. Young adults)            Middle-aged            Young-old            Old-old       Gender (ref. Male)            Female            Other       Ethnicity (ref. White)            Non-White            Prefer not to say       Deprivation (ref. Quintile 1 - Most deprived)            Quintile 2            Quintile 3            Quintile 4            Quintile 5 – Least deprived       Education (ref. Lower)            Middle            Higher            Other      Physical health (ref. Poor/fair)            Good            Very good/excellent | 0.631 (0.492, 0.809)  1.289 (0.875, 1.900)  1.410 (0.974, 2.041)  0.946 (0.593, 1.510)  0.631 (0.533, 0.747)  1.038 (0.215, 5.023)  0.883 (0.564, 1.381)  1.002 (0.211, 4.762)  1.222 (0.854, 1.749)  1.689 (1.182, 2.412)  1.320 (0.945, 1.845)  1.348 (0.968, 1.877)  0.965 (0.727, 1.282)  0.887 (0.691, 1.139)  0.908 (0.604, 1.364)  0.972 (0.779, 1.211)  1.342 (1.078, 1.672) | **p<0.001***  **p<0.05**  p>0.05  p>0.05  p>0.05  **p<0.001***  **p<0.001***  p>0.05  p>0.05  p>0.05  p>0.05  **p<0.05**  p>0.05  **p<0.01**  p>0.05  p>0.05  p>0.05  p>0.05  p>0.05  p>0.05  **p<0.001***  p>0.05  **p<0.01** |
| Using green and blue spaces | Preventability of cognitive frailty       Age (ref. Young adults)            Middle-aged            Young-old            Old-old       Gender (ref. Male)            Female            Other       Ethnicity (ref. White)            Non-White            Prefer not to say       Deprivation (ref. Quintile 1 - Most deprived)            Quintile 2            Quintile 3            Quintile 4            Quintile 5 – Least deprived       Education (ref. Lower)            Middle            Higher            Other      Physical health (ref. Poor/fair)            Good            Very good/excellent | 0.468 (0.306, 0.716)  1.321 (0.566, 3.084)  1.126 (0.507, 2.502)  0.638 (0.256, 1.591)  0.435 (0.311, 0.609)  0.136 (0.026, 0.701)  0.775 (0.329, 1.828)  0.370 (0.045, 3.014)  1.081 (0.553, 2.113)  1.777 (0.885, 3.568)  1.852 (0.954, 3.597)  1.335 (0.709, 2.512)  1.159 (0.645, 2.082)  0.890 (0.539, 1.471)  0.608 (0.294, 1.257)  1.619 (1.083, 2.421)  2.386 (1.570, 3.627) | **p<0.001***  p>0.05  p>0.05  p>0.05  p>0.05  **p<0.001***  **p<0.001***  **p<0.05**  p>0.05  p>0.05  p>0.05  p>0.05  p>0.05  p>0.05  p>0.05  p>0.05  p>0.05  p>0.05  p>0.05  p>0.05  **p<0.001***  **p<0.05**  **p<0.001*** |
| Healthy diet | Preventability of cognitive frailty       Age (ref. Young adults)            Middle-aged            Young-old            Old-old       Gender (ref. Male)            Female            Other       Ethnicity (ref. White)            Non-White            Prefer not to say       Deprivation (ref. Quintile 1 - Most deprived)            Quintile 2            Quintile 3            Quintile 4            Quintile 5 – Least deprived       Education (ref. Lower)            Middle            Higher            Other      Physical health (ref. Poor/fair)            Good            Very good/excellent | 0.289 (0.182, 0.459)  1.012 (0.410, 2.498)  1.248 (0.520, 2.995)  1.715 (0.525, 5.604)  0.416 (0.279, 0.619)  0.102 (0.019, 0.543)  0.574 (0.239, 1.382)  46749189.125 (0.000, -)  0.989 (0.451, 2.167)  1.306 (0.588, 2.900)  1.373 (0.640, 2.946)  1.777 (0.820, 3.852)  0.879 (0.445, 1.736)  0.723 (0.394, 1.325)  37756534.462 (0.000, -)  1.810 (1.121, 2.921)  2.341 (1.441, 3.805) | **p<0.001***  p>0.05  p>0.05  p>0.05  p>0.05  **p<0.001***  **p<0.001***  **p<0.01**  p>0.05  p>0.05  p>0.05  p>0.05  p>0.05  p>0.05  p>0.05  p>0.05  p>0.05  p>0.05  p>0.05  p>0.05  **p<0.01**  **p<0.05**  **p<0.001*** |
| Managing mental wellbeing | Preventability of cognitive frailty       Age (ref. Young adults)            Middle-aged            Young-old            Old-old       Gender (ref. Male)            Female            Other       Ethnicity (ref. White)            Non-White            Prefer not to say       Deprivation (ref. Quintile 1 - Most deprived)            Quintile 2            Quintile 3            Quintile 4            Quintile 5 – Least deprived       Education (ref. Lower)            Middle            Higher            Other      Physical health (ref. Poor/fair)            Good            Very good/excellent | 0.431 (0.306, 0.607)  0.815 (0.390, 1.704)  0.758 (0.372, 1.542)  0.685 (0.296, 1.582)  0.397 (0.308, 0.511)  79598531.647 (0.000, -)  0.702 (0.365, 1.349)  0.300 (0.062, 1.457)  0.923 (0.510, 1.671)  1.409 (0.775, 2.560)  1.034 (0.592, 1.804)  1.038 (0.599, 1.801)  1.217 (0.764, 1.940)  0.770 (0.520, 1.140)  1.147 (0.577, 2.279)  0.951 (0.669, 1.351)  1.068 (0.755, 1.511) | **p<0.001***  p>0.05  p>0.05  p>0.05  p>0.05  **p<0.001***  **p<0.001***  p>0.05  p>0.05  p>0.05  p>0.05  p>0.05  p>0.05  p>0.05  p>0.05  p>0.05  **p<0.05**  p>0.05  p>0.05  p>0.05  p>0.05  p>0.05  p>0.05 |
| Managing weight | Preventability of cognitive frailty       Age (ref. Young adults)            Middle-aged            Young-old            Old-old       Gender (ref. Male)            Female            Other       Ethnicity (ref. White)            Non-White            Prefer not to say       Deprivation (ref. Quintile 1 - Most deprived)            Quintile 2            Quintile 3            Quintile 4            Quintile 5 – Least deprived       Education (ref. Lower)            Middle            Higher            Other      Physical health (ref. Poor/fair)            Good            Very good/excellent | 0.475 (0.334, 0.675)  1.309 (0.697, 2.459)  1.222 (0.672, 2.222)  0.892 (0.430, 1.852)  0.577 (0.441, 0.754)  0.607 (0.074, 4.992)  0.640 (0.341, 1.200)  0.661 (0.082, 5.314)  1.001 (0.567, 1.766)  1.544 (0.863, 2.762)  1.235 (0.718, 2.123)  1.238 (0.725, 2.115)  1.180 (0.730, 1.907)  0.760 (0.507, 1.138)  0.896 (0.464, 1.730)  1.374 (0.993, 1.902)  2.151 (1.535, 3.014) | **p<0.001***  p>0.05  p>0.05  p>0.05  p>0.05  **p<0.001***  **p<0.001***  p>0.05  p>0.05  p>0.05  p>0.05  p>0.05  p>0.05  p>0.05  p>0.05  p>0.05  p>0.05  p>0.05  p>0.05  p>0.05  **p<0.001***  p>0.05  **p<0.001*** |
| Mindful activities | Preventability of cognitive frailty       Age (ref. Young adults)            Middle-aged            Young-old            Old-old       Gender (ref. Male)            Female            Other       Ethnicity (ref. White)            Non-White            Prefer not to say       Deprivation (ref. Quintile 1 - Most deprived)            Quintile 2            Quintile 3            Quintile 4            Quintile 5 – Least deprived       Education (ref. Lower)            Middle            Higher            Other      Physical health (ref. Poor/fair)            Good            Very good/excellent | 0.609 (0.488, 0.760)  0.887 (0.602, 1.306)  0.582 (0.402, 0.842)  0.395 (0.252, 0.619)  0.462 (0.401, 0.532)  0.803 (0.198, 3.256)  2.757 (1.660, 4.578)  0.571 (0.157, 2.078)  0.924 (0.668, 1.276)  1.156 (0.845, 1.582)  1.094 (0.809, 1.480)  1.049 (0.779, 1.414)  1.371 (1.093, 1.720)  1.542 (1.264, 1.882)  1.470 (1.053, 2.054)  0.928 (0.764, 1.127)  0.838 (0.695, 1.010) | **p<0.001***  **p<0.001***  p>0.05  **p<0.01**  **p<0.001***  **p<0.001***  **p<0.001***  p>0.05  **p<0.001***  **p<0.001***  p>0.05  p>0.05  p>0.05  p>0.05  p>0.05  p>0.05  **p<0.001***  **p<0.01**  **p<0.001***  **p<0.05**  p>0.05  p>0.05  p>0.05 |
| Physical activity | Preventability of cognitive frailty       Age (ref. Young adults)            Middle-aged            Young-old            Old-old       Gender (ref. Male)            Female            Other       Ethnicity (ref. White)            Non-White            Prefer not to say       Deprivation (ref. Quintile 1 - Most deprived)            Quintile 2            Quintile 3            Quintile 4            Quintile 5 – Least deprived       Education (ref. Lower)            Middle            Higher            Other      Physical health (ref. Poor/fair)            Good            Very good/excellent | 0.341 (0.244, 0.477)  1.191 (0.613, 2.314)  1.299 (0.687, 2.457)  1.149 (0.518, 2.551)  0.731 (0.545, 0.982)  0.679 (0.076, 6.030)  0.988 (0.444, 2.199)  105423549.80 (0.000, -)  1.709 (1.022, 2.856)  2.890 (1.677, 4.981)  2.515 (1.531, 4.133)  2.183 (1.351, 3.526)  1.262 (0.814, 1.958)  1.282 (0.872, 1.885)  1.187 (0.620, 2.273)  2.416 (1.749, 3.337)  4.347 (3.049, 6.197) | **p<0.001***  p>0.05  p>0.05  p>0.05  p>0.05  p>0.05  **p<0.05**  p>0.05  p>0.05  p>0.05  p>0.05  **p<0.001***  **p<0.05**  **p<0.001***  **p<0.001***  **p<0.01***  p>0.05  p>0.05  p>0.05  p>0.05  **p<0.001***  **p<0.001***  **p<0.001*** |
| Protecting against hearing loss | Preventability of cognitive frailty       Age (ref. Young adults)            Middle-aged            Young-old            Old-old       Gender (ref. Male)            Female            Other       Ethnicity (ref. White)            Non-White            Prefer not to say       Deprivation (ref. Quintile 1 - Most deprived)            Quintile 2            Quintile 3            Quintile 4            Quintile 5 – Least deprived       Education (ref. Lower)            Middle            Higher            Other      Physical health (ref. Poor/fair)            Good            Very good/excellent | 0.620 (0.484, 0.793)  1.304 (0.867, 1.961)  1.138 (0.773, 1.676)  0.775 (0.480, 1.250)  0.583 (0.492, 0.691)  0.963 (0.199, 4.653)  0.854 (0.545, 1.337)  1.077 (0.226, 5.147)  1.094 (0.765, 1.563)  1.937 (1.348, 2.785)  1.408 (1.005, 1.973)  1.360 (0.975, 1.896)  1.311 (0.991, 1.735)  1.062 (0.835, 1.351)  1.045 (0.702, 1.556)  1.138 (0.917, 1.412)  1.707 (1.375, 2.120) | **p<0.001***  **p<0.05**  p>0.05  p>0.05  p>0.05  **p<0.001***  **p<0.001***  p>0.05  p>0.05  p>0.05  p>0.05  **p<0.001***  p>0.05  **p<0.001***  **p<0.05**  p>0.05  p>0.05  p>0.05  p>0.05  p>0.05  **p<0.001***  p>0.05  **p<0.001*** |
| Purpose in life | Preventability of cognitive frailty       Age (ref. Young adults)            Middle-aged            Young-old            Old-old       Gender (ref. Male)            Female            Other       Ethnicity (ref. White)            Non-White            Prefer not to say       Deprivation (ref. Quintile 1 - Most deprived)            Quintile 2            Quintile 3            Quintile 4            Quintile 5 – Least deprived       Education (ref. Lower)            Middle            Higher            Other      Physical health (ref. Poor/fair)            Good            Very good/excellent | 0.466 (0.331, 0.658)  1.465 (0.847, 2.533)  1.567 (0.932, 2.633)  2.270 (1.077, 4.782)  0.489 (0.379, 0.631)  0.121 (0.032, 0.457)  0.846 (0.451, 1.587)  0.752 (0.094, 6.041)  0.909 (0.544, 1.520)  1.972 (1.138, 3.417)  1.512 (0.913, 2.505)  1.361 (0.832, 2.227)  1.261 (0.784, 2.026)  0.691 (0.465, 1.026)  1.054 (0.529, 2.100)  1.063 (0.767, 1.473)  1.609 (1.153, 2.246) | **p<0.001***  p>0.05  p>0.05  p>0.05  **p<0.05**  **p<0.001***  **p<0.001***  **p<0.01**  p>0.05  p>0.05  p>0.05  **p<0.01**  p>0.05  **p<0.05**  p>0.05  p>0.05  **p<0.01**  p>0.05  p>0.05  p>0.05  **p<0.01**  p>0.05  **p<0.01** |
| Not smoking or vaping | Preventability of cognitive frailty       Age (ref. Young adults)            Middle-aged            Young-old            Old-old       Gender (ref. Male)            Female            Other       Ethnicity (ref. White)            Non-White            Prefer not to say       Deprivation (ref. Quintile 1 - Most deprived)            Quintile 2            Quintile 3            Quintile 4            Quintile 5 – Least deprived       Education (ref. Lower)            Middle            Higher            Other      Physical health (ref. Poor/fair)            Good            Very good/excellent | 0.647 (0.485, 0.863)  1.013 (0.636, 1.613)  1.099 (0.703, 1.716)  1.007 (0.567, 1.789)  0.791 (0.647, 0.968)  263791773.86 (0.000, -)  0.730 (0.446, 1.195)  0.355 (0.091, 1.393)  0.997 (0.660, 1.506)  1.598 (1.052, 2.428)  1.422 (0.958, 2.110)  1.324 (0.899, 1.951)  1.382 (0.982, 1.946)  0.897 (0.675, 1.193)  1.035 (0.642, 1.670)  1.257 (0.975, 1.621)  1.472 (1.147, 1.890) | **p<0.01**  p>0.05  p>0.05  p>0.05  p>0.05  p>0.05  **p<0.05**  p>0.05  p>0.05  p>0.05  p>0.05  **p<0.05**  p>0.05  **p<0.05**  p>0.05  p>0.05  **p<0.05**  p>0.05  p>0.05  p>0.05  **p<0.05**  p>0.05  **p<0.01** |
| Socialising | Preventability of cognitive frailty       Age (ref. Young adults)            Middle-aged            Young-old            Old-old       Gender (ref. Male)            Female            Other       Ethnicity (ref. White)            Non-White            Prefer not to say       Deprivation (ref. Quintile 1 - Most deprived)            Quintile 2            Quintile 3            Quintile 4            Quintile 5 – Least deprived       Education (ref. Lower)            Middle            Higher            Other      Physical health (ref. Poor/fair)            Good            Very good/excellent | 0.582 (0.415, 0.816)  1.227 (0.705, 2.136)  1.534 (0.900, 2.613)  1.663 (0.827, 3.344)  0.409 (0.322, 0.521)  0.176 (0.042, 0.728)  1.018 (0.531, 1.949)  0.253 (0.063, 1.012)  0.859 (0.506, 1.459)  1.198 (0.702, 2.046)  1.147 (0.688, 1.911)  1.056 (0.639, 1.744)  1.122 (0.733, 1.716)  0.804 (0.558, 1.160)  2.005 (0.915, 4.396)  1.434 (1.068, 1.925)  2.379 (1.751, 3.231) | **p<0.01**  p>0.05  p>0.05  p>0.05  p>0.05  **p<0.001***  **p<0.001***  **p<0.05**  p>0.05  p>0.05  p>0.05  p>0.05  p>0.05  p>0.05  p>0.05  p>0.05  **p<0.05**  p>0.05  p>0.05  p>0.05  **p<0.001***  **p<0.05**  **p<0.001*** |
| Taking vitamins and supplements | Preventability of cognitive frailty       Age (ref. Young adults)            Middle-aged            Young-old            Old-old       Gender (ref. Male)            Female            Other       Ethnicity (ref. White)            Non-White            Prefer not to say       Deprivation (ref. Quintile 1 - Most deprived)            Quintile 2            Quintile 3            Quintile 4            Quintile 5 – Least deprived       Education (ref. Lower)            Middle            Higher            Other      Physical health (ref. Poor/fair)            Good            Very good/excellent | 0.676 (0.524, 0.872)  0.681 (0.401, 1.157)  0.463 (0.278, 0.770)  0.240 (0.135, 0.426)  0.489 (0.415, 0.577)  0.332 (0.080, 1.389)  1.330 (0.780, 2.266)  0.962 (0.199, 4.655)  0.821 (0.545, 1.236)  0.753 (0.508, 1.115)  0.856 (0.584, 1.256)  0.926 (0.633, 1.353)  1.194 (0.902, 1.580)  0.985 (0.773, 1.255)  1.076 (0.720, 1.608)  1.022 (0.808, 1.292)  0.831 (0.665, 1.039) | **p<0.01**  **p<0.001***  p>0.05  **p<0.01**  **p<0.001***  **p<0.001***  **p<0.001***  p>0.05  p>0.05  p>0.05  p>0.05  p>0.05  p>0.05  p>0.05  p>0.05  p>0.05  p>0.05  p>0.05  p>0.05  p>0.05  p>0.05  p>0.05  p>0.05 |
| Volunteering and helping others | Preventability of cognitive frailty       Age (ref. Young adults)            Middle-aged            Young-old            Old-old       Gender (ref. Male)            Female            Other       Ethnicity (ref. White)            Non-White            Prefer not to say       Deprivation (ref. Quintile 1 - Most deprived)            Quintile 2            Quintile 3            Quintile 4            Quintile 5 – Least deprived       Education (ref. Lower)            Middle            Higher            Other      Physical health (ref. Poor/fair)            Good            Very good/excellent | 0.554 (0.431, 0.711)  1.433 (0.952, 2.155)  1.608 (1.089, 2.376)  1.906 (1.134, 3.202)  0.450 (0.378, 0.536)  0.804 (0.162, 3.988)  1.118 (0.676, 1.850)  1.018 (0.210, 4.937)  1.052 (0.717, 1.544)  1.518 (1.034, 2.230)  1.156 (0.807, 1.656)  1.048 (0.736, 1.492)  1.112 (0.843, 1.466)  1.344 (1.050, 1.720)  2.198 (1.348, 3.587)  1.598 (1.279, 1.997)  1.965 (1.579, 2.444) | **p<0.001***  p>0.05  p>0.05  **p<0.05**  **p<0.05**  **p<0.001***  **p<0.001***  p>0.05  p>0.05  p>0.05  p>0.05  **p<0.05**  p>0.05  **p<0.05**  p>0.05  p>0.05  **p<0.01**  p>0.05  **p<0.05**  **p<0.01**  **p<0.001***  **p<0.001***  **p<0.001*** |

*Note.* Bolded comparisons are significant at p<0.05. * denotes comparisons remaining significant after Bonferroni correction at p<0.002.

# Supplementary Table 7: Future behaviours - By importance of factors

| Dependent variable | Predictor and covariates | Odds Ratio (95% CI) | P-value |
| --- | --- | --- | --- |
| Drinking 2 litres of water | Importance of drinking 2 litres of water       Age (ref. Young adults)            Middle-aged            Young-old            Old-old       Gender (ref. Male)            Female            Other       Ethnicity (ref. White)            Non-White            Prefer not to say      Deprivation (ref. Quintile 1 - Most deprived)            Quintile 2            Quintile 3            Quintile 4            Quintile 5 – Least deprived       Education (ref. Lower)            Middle            Higher            Other      Physical health (ref. Poor/fair)            Good            Very good/excellent | 12.232 (9.493, 15.759)  0.462 (0.173, 1.233)  0.298 (0.115, 0.773)  0.146 (0.053, 0.400)  0.471 (0.373, 0.596)  0.197 (0.029, 1.345)  1.218 (0.552, 2.688)  1.188 (0.124, 11.418)  1.190 (0.681, 2.079)  1.106 (0.649, 1.885)  1.310 (0.779, 2.202)  1.191 (0.717, 1.980)  1.454 (0.974, 2.171)  1.259 (0.897, 1.767)  1.946 (1.016, 3.728)  1.252 (0.906, 1.730)  1.190 (0.874, 1.621) | **p<0.001***  **p<0.001***  p>0.05  **p<0.05**  **p<0.001***  **p<0.001***  **p<0.001***  p>0.05  p>0.05  p>0.05  p>0.05  p>0.05  p>0.05  p>0.05  p>0.05  p>0.05  p>0.05  p>0.05  p>0.05  **p<0.05**  p>0.05  p>0.05  p>0.05 |
| Drinking less or no alcohol | Importance of drinking less or no alcohol       Age (ref. Young adults)            Middle-aged            Young-old            Old-old       Gender (ref. Male)            Female            Other       Ethnicity (ref. White)            Non-White            Prefer not to say       Deprivation (ref. Quintile 1 - Most deprived)            Quintile 2            Quintile 3            Quintile 4            Quintile 5 – Least deprived       Education (ref. Lower)            Middle            Higher            Other      Physical health (ref. Poor/fair)            Good            Very good/excellent | 5.419 (4.170, 7.043)  1.147 (0.756, 1.742)  0.929 (0.625, 1.380)  0.677 (0.416, 1.100)  0.476 (0.406, 0.559)  2.667 (0.299, 23.828)  1.395 (0.848, 2.296)  0.443 (0.121, 1.615)  0.851 (0.587, 1.235)  1.049 (0.730, 1.508)  1.071 (0.754, 1.521)  1.087 (0.769, 1.538)  1.016 (0.773, 1.335)  0.908 (0.715, 1.152)  1.093 (0.727, 1.643)  0.960 (0.772, 1.193)  1.213 (0.981, 1.501) | **p<0.001***  **p<0.05**  p>0.05  p>0.05  p>0.05  **p<0.001***  **p<0.001***  p>0.05  p>0.05  p>0.05  p>0.05  p>0.05  p>0.05  p>0.05  p>0.05  p>0.05  p>0.05  p>0.05  p>0.05  p>0.05  **p<0.05**  p>0.05  p>0.05 |
| Educational classes | Importance of educational classes       Age (ref. Young adults)            Middle-aged            Young-old            Old-old       Gender (ref. Male)            Female            Other       Ethnicity (ref. White)            Non-White            Prefer not to say       Deprivation (ref. Quintile 1 - Most deprived)            Quintile 2            Quintile 3            Quintile 4            Quintile 5 – Least deprived       Education (ref. Lower)            Middle            Higher            Other      Physical health (ref. Poor/fair)            Good            Very good/excellent | 4.417 (3.787, 5.153)  1.234 (0.833, 1.829)  0.969 (0.666, 1.410)  0.944 (0.589, 1.514)  0.587 (0.503, 0.687)  2.029 (0.250, 16.479)  1.272 (0.798, 2.027)  0.310 (0.079, 1.215)  1.064 (0.753, 1.505)  1.350 (0.963, 1.894)  1.143 (0.828, 1.580)  1.060 (0.770, 1.458)  1.669 (1.314, 2.119)  2.366 (1.916, 2.922)  1.490 (1.051, 2.111)  1.368 (1.118, 1.674)  1.569 (1.291, 1.907) | **p<0.001***  p>0.05  p>0.05  p>0.05  p>0.05  **p<0.001***  **p<0.001***  p>0.05  p>0.05  p>0.05  p>0.05  p>0.05  p>0.05  p>0.05  p>0.05  p>0.05  **p<0.001***  **p<0.001***  **p<0.001***  **p<0.05**  **p<0.001***  **p<0.01**  **p<0.001*** |
| Limiting exposure to air pollution | Importance of limiting exposure to air pollution       Age (ref. Young adults)            Middle-aged            Young-old            Old-old       Gender (ref. Male)            Female            Other       Ethnicity (ref. White)            Non-White            Prefer not to say       Deprivation (ref. Quintile 1 - Most deprived)            Quintile 2            Quintile 3            Quintile 4            Quintile 5 – Least deprived       Education (ref. Lower)            Middle            Higher            Other      Physical health (ref. Poor/fair)            Good            Very good/excellent | 6.694 (5.504, 8.142)  1.364 (0.902, 2.061)  1.375 (0.927, 2.037)  0.900 (0.548, 1.477)  0.699 (0.585, 0.837)  1.007 (0.189, 5.364)  0.846 (0.526, 1.359)  0.920 (0.180, 4.694)  1.274 (0.872, 1.861)  1.761 (1.209, 2.564)  1.422 (0.998, 2.025)  1.409 (0.994, 1.999)  0.951 (0.706, 1.282)  0.934 (0.719, 1.213)  0.863 (0.562, 1.326)  0.906 (0.717, 1.145)  1.304 (1.035, 1.642) | **p<0.001***  **p<0.05**  p>0.05  p>0.05  p>0.05  **p<0.001***  **p<0.001***  p>0.05  p>0.05  p>0.05  p>0.05  **p<0.05**  p>0.05  **p<0.01**  p>0.05  p>0.05  p>0.05  p>0.05  p>0.05  p>0.05  **p<0.001***  p>0.05  **p<0.05** |
| Using green and blue spaces | Importance of using green spaces  Importance of being near water       Age (ref. Young adults)            Middle-aged            Young-old            Old-old       Gender (ref. Male)            Female            Other       Ethnicity (ref. White)            Non-White            Prefer not to say       Deprivation (ref. Quintile 1 - Most deprived)            Quintile 2            Quintile 3            Quintile 4            Quintile 5 – Least deprived       Education (ref. Lower)            Middle            Higher            Other      Physical health (ref. Poor/fair)            Good            Very good/excellent | 4.353 (2.844, 6.665)  2.023 (1.377, 2.970)  1.229 (0.516, 2.928)  0.996 (0.440, 2.254)  0.545 (0.213, 1.392)  0.553 (0.390, 0.783)  0.105 (0.019, 0.563)  0.856 (0.353, 2.073)  0.416 (0.046, 3.749)  1.125 (0.565, 2.240)  1.701 (0.831, 3.483)  1.706 (0.863, 3.372)  1.293 (0.674, 2.478)  1.253 (0.690, 2.275)  1.026 (0.616, 1.707)  0.575 (0.275, 1.201)  1.595 (1.059, 2.404)  2.446 (1.602, 3.734) | **p<0.001***  **p<0.001***  p>0.05  p>0.05  p>0.05  p>0.05  **p<0.001***  **p<0.001***  **p<0.01**  p>0.05  p>0.05  p>0.05  p>0.05  p>0.05  p>0.05  p>0.05  p>0.05  p>0.05  p>0.05  p>0.05  p>0.05  **p<0.001***  **p<0.05**  **p<0.001*** |
| Healthy diet | Importance of eating fruits and vegetables  Importance of eating less red meat  Importance of eating nuts, seeds and legumes  Importance of eating less processed foods       Age (ref. Young adults)            Middle-aged            Young-old            Old-old       Gender (ref. Male)            Female            Other       Ethnicity (ref. White)            Non-White            Prefer not to say       Deprivation (ref. Quintile 1 - Most deprived)            Quintile 2            Quintile 3            Quintile 4            Quintile 5 – Least deprived       Education (ref. Lower)            Middle            Higher            Other      Physical health (ref. Poor/fair)            Good            Very good/excellent | 3.353 (1.591, 7.066)  1.147 (0.700, 1.879)  1.731 (0.934, 3.206)  3.283 (1.720, 6.265)  0.929 (0.361, 2.391)  1.103 (0.442, 2.751)  1.354 (0.399, 4.587)  0.498 (0.329, 0.752)  0.113 (0.019, 0.654)  0.490 (0.203, 1.184)  58593492.874 (0.000, -)  0.981 (0.436, 2.206)  1.085 (0.477, 2.468)  1.238 (0.563, 2.725)  1.647 (0.745, 3.641)  0.899 (0.447, 1.806)  0.801 (0.432, 1.486)  34272757.260 (0.000, -)  1.763 (1.078, 2.883)  2.251 (1.372, 3.693) | **p<0.01***  p>0.05  p>0.05  **p<0.001***  p>0.05  p>0.05  p>0.05  p>0.05  **p<0.001***  **p<0.001***  **p<0.05**  p>0.05  p>0.05  p>0.05  p>0.05  p>0.05  p>0.05  p>0.05  p>0.05  p>0.05  p>0.05  p>0.05  p>0.05  **p<0.01**  **p<0.05**  **p<0.01*** |
| Managing mental wellbeing | Importance of managing mental wellbeing       Age (ref. Young adults)            Middle-aged            Young-old            Old-old       Gender (ref. Male)            Female            Other       Ethnicity (ref. White)            Non-White            Prefer not to say       Deprivation (ref. Quintile 1 - Most deprived)            Quintile 2            Quintile 3            Quintile 4            Quintile 5 – Least deprived       Education (ref. Lower)            Middle            Higher            Other      Physical health (ref. Poor/fair)            Good            Very good/excellent | 11.893 (6.766, 20.905)  0.774 (0.363, 1.649)  0.733 (0.353, 1.521)  0.633 (0.269, 1.490)  0.415 (0.321, 0.536)  72080361.669 (0.000, -)  0.707 (0.366, 1.368)  0.316 (0.065, 1.534)  0.870 (0.474, 1.598)  1.282 (0.695, 2.364)  0.927 (0.523, 1.641)  0.985 (0.559, 1.734)  1.350 (0.841, 2.165)  0.846 (0.571, 1.254)  1.096 (0.550, 2.183)  1.007 (0.707, 1.435)  1.184 (0.837, 1.676) | **p<0.001***  p>0.05  p>0.05  p>0.05  p>0.05  **p<0.001***  **p<0.001***  p>0.05  p>0.05  p>0.05  p>0.05  p>0.05  p>0.05  p>0.05  p>0.05  p>0.05  p>0.05  p>0.05  p>0.05  p>0.05  p>0.05  p>0.05  p>0.05 |
| Managing weight | Importance of managing weight       Age (ref. Young adults)            Middle-aged            Young-old            Old-old       Gender (ref. Male)            Female            Other       Ethnicity (ref. White)            Non-White            Prefer not to say       Deprivation (ref. Quintile 1 - Most deprived)            Quintile 2            Quintile 3            Quintile 4            Quintile 5 – Least deprived       Education (ref. Lower)            Middle            Higher            Other      Physical health (ref. Poor/fair)            Good            Very good/excellent | 6.633 (4.681, 9.400)  1.137 (0.593, 2.180)  1.089 (0.587, 2.021)  0.772 (0.363, 1.641)  0.586 (0.446, 0.770)  0.909 (0.099, 8.352)  0.548 (0.291, 1.032)  0.598 (0.074, 4.807)  0.975 (0.545, 1.744)  1.433 (0.790, 2.598)  1.201 (0.688, 2.096)  1.216 (0.702, 2.106)  1.317 (0.809, 2.144)  0.848 (0.564, 1.274)  0.838 (0.432, 1.627)  1.402 (1.007, 1.953)  2.179 (1.550, 3.062) | **p<0.001***  p>0.05  p>0.05  p>0.05  p>0.05  **p<0.001***  **p<0.001***  p>0.05  p>0.05  p>0.05  p>0.05  p>0.05  p>0.05  p>0.05  p>0.05  p>0.05  p>0.05  p>0.05  p>0.05  p>0.05  **p<0.001***  **p<0.05**  **p<0.001*** |
| Mindful activities | Importance of mindful activities       Age (ref. Young adults)            Middle-aged            Young-old            Old-old       Gender (ref. Male)            Female            Other       Ethnicity (ref. White)            Non-White            Prefer not to say       Deprivation (ref. Quintile 1 - Most deprived)            Quintile 2            Quintile 3            Quintile 4            Quintile 5 – Least deprived       Education (ref. Lower)            Middle            Higher            Other      Physical health (ref. Poor/fair)            Good            Very good/excellent | 7.833 (6.740, 9.105)  0.925 (0.604, 1.416)  0.642 (0.427, 0.964)  0.437 (0.266, 0.718)  0.567 (0.484, 0.665)  0.400 (0.099, 1.621)  2.370 (1.368, 4.104)  0.589 (0.144, 2.406)  0.922 (0.644, 1.319)  1.206 (0.851, 1.709)  1.075 (0.769, 1.502)  1.026 (0.737, 1.429)  1.467 (1.139, 1.890)  1.607 (1.288, 2.004)  1.518 (1.044, 2.208)  1.028 (0.829, 1.276)  0.957 (0.779, 1.176) | **p<0.001***  **p<0.001***  p>0.05  **p<0.05**  **p<0.01***  **p<0.001***  **p<0.001***  p>0.05  **p<0.01**  **p<0.01**  p>0.05  p>0.05  p>0.05  p>0.05  p>0.05  p>0.05  **p<0.001***  **p<0.01**  **p<0.001***  **p<0.05**  p>0.05  p>0.05  p>0.05 |
| Physical activity | Importance of strength training  Importance of cardiovascular exercises  Importance of balance exercises       Age (ref. Young adults)            Middle-aged            Young-old            Old-old       Gender (ref. Male)            Female            Other       Ethnicity (ref. White)            Non-White            Prefer not to say       Deprivation (ref. Quintile 1 - Most deprived)            Quintile 2            Quintile 3            Quintile 4            Quintile 5 – Least deprived       Education (ref. Lower)            Middle            Higher            Other      Physical health (ref. Poor/fair)            Good            Very good/excellent | 1.930 (1.360, 2.738)  2.544 (1.809, 3.577)  2.397 (1.700, 3.379)  1.158 (0.583, 2.300)  1.532 (0.793, 2.963)  1.765 (0.773, 4.030)  0.878 (0.641, 1.201)  0.414 (0.046, 3.742)  1.054 (0.455, 2.442)  115908532.91 (0.000, -)  1.612 (0.943, 2.758)  2.650 (1.506, 4.662)  2.444 (1.455, 4.105)  2.071 (1.254, 3.421)  1.278 (0.812, 2.010)  1.167 (0.785, 1.734)  1.147 (0.590, 2.227)  2.344 (1.679, 3.271)  3.861 (2.689, 5.544) | **p<0.001***  **p<0.001***  **p<0.001***  p>0.05  p>0.05  p>0.05  p>0.05  p>0.05  p>0.05  p>0.05  p>0.05  p>0.05  p>0.05  **p<0.01**  p>0.05  **p<0.001***  **p<0.001***  **p<0.01**  p>0.05  p>0.05  p>0.05  p>0.05  **p<0.001***  **p<0.001***  **p<0.001*** |
| Protecting against hearing loss | Importance of protecting against hearing loss  Importance of wearing hearing aids       Age (ref. Young adults)            Middle-aged            Young-old            Old-old       Gender (ref. Male)            Female            Other       Ethnicity (ref. White)            Non-White            Prefer not to say       Deprivation (ref. Quintile 1 - Most deprived)            Quintile 2            Quintile 3            Quintile 4            Quintile 5 – Least deprived       Education (ref. Lower)            Middle            Higher            Other      Physical health (ref. Poor/fair)            Good            Very good/excellent | 3.512 (2.864, 4.307)  1.641 (1.332, 2.021)  1.149 (0.748, 1.766)  0.954 (0.633, 1.436)  0.737 (0.445, 1.221)  0.715 (0.597, 0.857)  0.918 (0.173, 4.885)  0.877 (0.548, 1.403)  1.406 (0.281, 7.047)  1.163 (0.799, 1.691)  1.835 (1.256, 2.680)  1.378 (0.968, 1.964)  1.369 (0.966, 1.940)  1.325 (0.990, 1.773)  1.078 (0.840, 1.383)  1.003 (0.662, 1.519)  1.128 (0.901, 1.414)  1.736 (1.387, 2.173) | **p<0.001***  **p<0.001***  p>0.05  p>0.05  p>0.05  p>0.05  **p<0.01***  **p<0.001***  p>0.05  p>0.05  p>0.05  p>0.05  **p<0.01**  p>0.05  **p<0.01**  p>0.05  p>0.05  p>0.05  p>0.05  p>0.05  p>0.05  **p<0.001***  p>0.05  **p<0.001*** |
| Purpose in life | Importance of having a purpose in life  Age (ref. Young adults)            Middle-aged            Young-old            Old-old       Gender (ref. Male)            Female            Other       Ethnicity (ref. White)            Non-White            Prefer not to say       Deprivation (ref. Quintile 1 - Most deprived)            Quintile 2            Quintile 3            Quintile 4            Quintile 5 – Least deprived       Education (ref. Lower)            Middle            Higher            Other      Physical health (ref. Poor/fair)            Good            Very good/excellent | 12.049 (8.097, 17.931)  1.076 (0.600, 1.932)  1.135 (0.651, 1.982)  1.493 (0.686, 3.253)  0.534 (0.410, 0.696)  0.113 (0.028, 0.450)  0.741 (0.392, 1.400)  0.682 (0.085, 5.478)  0.831 (0.483, 1.429)  1.577 (0.888, 2.800)  1.320 (0.777, 2.244)  1.310 (0.779, 2.202)  1.225 (0.749, 2.006)  0.679 (0.451, 1.023)  0.913 (0.451, 1.848)  1.082 (0.772, 1.518)  1.660 (1.180, 2.337) | **p<0.001***  p>0.05  p>0.05  p>0.05  p>0.05  **p<0.001***  **p<0.001***  **p<0.01**  p>0.05  p>0.05  p>0.05  **p<0.05**  p>0.05  p>0.05  p>0.05  p>0.05  **p<0.01**  p>0.05  p>0.05  p>0.05  **p<0.01**  p>0.05  **p<0.01** |
| Not smoking or vaping | Importance of not smoking or vaping       Age (ref. Young adults)            Middle-aged            Young-old            Old-old       Gender (ref. Male)            Female            Other       Ethnicity (ref. White)            Non-White            Prefer not to say       Deprivation (ref. Quintile 1 - Most deprived)            Quintile 2            Quintile 3            Quintile 4            Quintile 5 – Least deprived       Education (ref. Lower)            Middle            Higher            Other      Physical health (ref. Poor/fair)            Good            Very good/excellent | 4.184 (2.682, 6.528)  1.038 (0.649, 1.659)  1.073 (0.684, 1.683)  0.976 (0.547, 1.740)  0.814 (0.665, 0.997)  251246828.32 (0.000, -)  0.735 (0.448, 1.206)  0.356 (0.091, 1.398)  1.033 (0.681, 1.566)  1.583 (1.040, 2.410)  1.413 (0.950, 2.102)  1.315 (0.891, 1.941)  1.443 (1.024, 2.033)  0.931 (0.701, 1.236)  1.054 (0.651, 1.706)  1.316 (1.020, 1.698)  1.538 (1.199, 1.971) | **p<0.001***  p>0.05  p>0.05  p>0.05  p>0.05  p>0.05  **p<0.05**  p>0.05  p>0.05  p>0.05  p>0.05  p>0.05  p>0.05  **p<0.05**  p>0.05  p>0.05  **p<0.05**  **p<0.05**  p>0.05  p>0.05  **p<0.01**  **p<0.05**  **p<0.001*** |
| Socialising | Importance of socialising       Age (ref. Young adults)            Middle-aged            Young-old            Old-old       Gender (ref. Male)            Female            Other       Ethnicity (ref. White)            Non-White            Prefer not to say       Deprivation (ref. Quintile 1 - Most deprived)            Quintile 2            Quintile 3            Quintile 4            Quintile 5 – Least deprived       Education (ref. Lower)            Middle            Higher            Other      Physical health (ref. Poor/fair)            Good            Very good/excellent | 9.452 (6.751, 13.233)  1.182 (0.667, 2.093)  1.539 (0.889, 2.665)  1.932 (0.932, 4.005)  0.479 (0.373, 0.617)  0.144 (0.034, 0.600)  0.967 (0.496, 1.883)  0.206 (0.051, 0.822)  0.774 (0.445, 1.347)  1.092 (0.624, 1.913)  1.030 (0.604, 1.756)  0.934 (0.552, 1.579)  1.054 (0.675, 1.644)  0.716 (0.488, 1.053)  1.652 (0.740, 3.692)  1.369 (1.007, 1.861)  2.285 (1.666, 3.135) | **p<0.001***  p>0.05  p>0.05  p>0.05  p>0.05  **p<0.001***  **p<0.001***  **p<0.01**  p>0.05  p>0.05  **p<0.05**  p>0.05  p>0.05  p>0.05  p>0.05  p>0.05  **p<0.05**  p>0.05  p>0.05  p>0.05  **p<0.001***  **p<0.05**  **p<0.001*** |
| Taking vitamins and supplements | Importance of taking vitamins and supplements       Age (ref. Young adults)            Middle-aged            Young-old            Old-old       Gender (ref. Male)            Female            Other       Ethnicity (ref. White)            Non-White            Prefer not to say       Deprivation (ref. Quintile 1 - Most deprived)            Quintile 2            Quintile 3            Quintile 4            Quintile 5 – Least deprived       Education (ref. Lower)            Middle            Higher            Other      Physical health (ref. Poor/fair)            Good            Very good/excellent | 9.166 (7.436, 11.298)  0.911 (0.520, 1.597)  0.655 (0.382, 1.124)  0.304 (0.165, 0.563)  0.535 (0.448, 0.639)  0.481 (0.103, 2.251)  1.056 (0.594, 1.879)  0.630 (0.111, 3.582)  0.961 (0.617, 1.495)  0.821 (0.537, 1.255)  0.956 (0.633, 1.446)  0.991 (0.658, 1.493)  1.200 (0.885, 1.627)  1.262 (0.971, 1.640)  1.088 (0.699, 1.693)  1.152 (0.895, 1.483)  0.994 (0.784, 1.262) | **p<0.001***  **p<0.001***  p>0.05  p>0.05  **p<0.001***  **p<0.001***  **p<0.001***  p>0.05  p>0.05  p>0.05  p>0.05  p>0.05  p>0.05  p>0.05  p>0.05  p>0.05  p>0.05  p>0.05  p>0.05  p>0.05  p>0.05  p>0.05  p>0.05 |
| Volunteering and helping others | Importance of volunteering and helping others       Age (ref. Young adults)            Middle-aged            Young-old            Old-old       Gender (ref. Male)            Female            Other       Ethnicity (ref. White)            Non-White            Prefer not to say       Deprivation (ref. Quintile 1 - Most deprived)            Quintile 2            Quintile 3            Quintile 4            Quintile 5 – Least deprived       Education (ref. Lower)            Middle            Higher            Other      Physical health (ref. Poor/fair)            Good            Very good/excellent | 6.374 (5.294, 7.675)  1.260 (0.816, 1.945)  1.184 (0.782, 1.795)  1.156 (0.668, 2.000)  0.517 (0.429, 0.623)  0.675 (0.129, 3.522)  0.999 (0.586, 1.704)  1.406 (0.257, 7.692)  1.092 (0.725, 1.646)  1.405 (0.933, 2.115)  1.096 (0.746, 1.610)  0.991 (0.679, 1.447)  1.121 (0.836, 1.503)  1.363 (1.051, 1.767)  2.027 (1.214, 3.385)  1.750 (1.381, 2.218)  2.169 (1.723, 2.732) | **p<0.001***  p>0.05  p>0.05  p>0.05  p>0.05  **p<0.001***  **p<0.001***  p>0.05  p>0.05  p>0.05  p>0.05  p>0.05  p>0.05  p>0.05  p>0.05  p>0.05  **p<0.05**  p>0.05  **p<0.05**  **p<0.01**  **p<0.001***  **p<0.001***  **p<0.001*** |

*Note.* Bolded comparisons are significant at p<0.05. * denotes comparisons remaining significant after Bonferroni correction at p<0.002.
